# Patterns of human social contact and contact with animals in Shanghai, China

**DOI:** 10.1038/s41598-019-51609-8

**Published:** 2019-10-22

**Authors:** Juanjuan Zhang, Petra Klepac, Jonathan M. Read, Alicia Rosello, Xiling Wang, Shengjie Lai, Meng Li, Yujian Song, Qingzhen Wei, Hao Jiang, Juan Yang, Henry Lynn, Stefan Flasche, Mark Jit, Hongjie Yu

**Affiliations:** 1 School of Public Health, Fudan University, Key Laboratory of Public Health Safety, Ministry of Education, Shanghai, China; 2 Department of Infectious Disease Epidemiology, Faculty of Epidemiology and Public Health, London School of Hygiene and Tropical Medicine, London, UK; 3 Centre for Health Informatics, Computation and Statistics, Lancaster Medical School, Lancaster University, Lancashire, UK; 4 WorldPop, School of Geography and Environmental Science, University of Southampton, Southampton, UK; 5 Flowminder Foundation, Stockholm, Sweden; 6 Modelling and Economics Unit, Public Health England, London, UK; 7 School of Public Health, University of Hong Kong, Hong Kong, China

**Keywords:** Infectious diseases, Statistics

## Abstract

East Asia is as a principal hotspot for emerging zoonotic infections. Understanding the likely pathways for their emergence and spread requires knowledge on human-human and human-animal contacts, but such studies are rare. We used self-completed and interviewer-completed contact diaries to quantify patterns of these contacts for 965 individuals in 2017/2018 in a high-income densely-populated area of China, Shanghai City. Interviewer-completed diaries recorded more social contacts (19.3 vs. 18.0) and longer social contact duration (35.0 vs. 29.1 hours) than self-reporting. Strong age-assortativity was observed in all age groups especially among young participants (aged 7–20) and middle aged participants (25–55 years). 17.7% of participants reported touching animals (15.3% (pets), 0.0% (poultry) and 0.1% (livestock)). Human-human contact was very frequent but contact with animals (especially poultry) was rare although associated with frequent human-human contact. Hence, this densely populated area is more likely to act as an accelerator for human-human spread but less likely to be at the source of a zoonosis outbreak. We also propose that telephone interview at the end of reporting day is a potential improvement of the design of future contact surveys.

## Introduction

Most of the major global infectious disease threats of the last decade (including epidemics of pandemic influenza A/H1N1, Middle East respiratory syndrome coronavirus and Ebola) have emerged from pathogens crossing the species barrier from animals to humans. A principal hotspot for such disease emergence is the megacities of East Asia^1^. For these diseases, successful establishment in human populations is dictated by both animal-to-human (most notably with domesticated animals raised in agricultural settings such as poultry, swine and cattle) and human-to-human contact^2^. However, studies measuring and characterizing contacts on human-animal interface and subsequent transmission among humans are scarce. For emerging pathogens with established human-human transmission, the spread or even outbreak in humans will be driven by patterns of human encounters, which can also determine the effectiveness of interventions against them (e.g. vaccination, contact tracing and social distancing)^3^. Patterns of human encounters are location specific, therefore improving local intervention strategies relies on understanding local patterns of social contact^4–8^. There are still, however, relatively few empirical studies of age-specific social contacts which integrate location, animal contact data, and the interaction between human-human and human-animal contact. Furthermore, an evaluation of public health strategies that is based on social contact information derived from a different country that might not be representative of local mixing and may result in erroneous conclusions^9^.

China is a large, diverse country with rapid economic development, high urbanization, and frequent interaction between humans and animals. The richest and most populous city is Shanghai with a population of 24 million. We used two types of questionnaire delivery (self-completed and telephone interview) to quantify social contact patterns in Shanghai City, which is a hub for spreading infectious diseases due to its high population density and connectivity of the air transportation network.

Large population-based surveys of age-specific social contacts exists for several European countries^10–14^ and are increasingly conducted in low- and middle-income Asian (Thailand, Vietnam and Indonesia)^15,16^ and African countries (Zimbabwe and South Africa^17–19^, Kenya^20^, Uganda^21^). There have also been similar surveys in Russia^22^, Australia^23,24^, Peru^25^, Japan^26^, Taiwan^27^, southern China (Guangzhou^28^, Hong Kong^29–31^). Data from such contact studies are increasingly made freely available for download^32^. These surveys have their limitations, so recently there have been proposals for improved designs that would measure social contact patterns with less recall bias. Recent technological developments allow the measurement of human proximity and social interactions using wearable sensors, but such surveys are limited to specific settings, such as households^33^ or schools^34^ and require high levels of participation within such settings to succeed. Some studies encouraged participants to record each contact as it occurred^11,17,20,29^, but such prospectively recording has been found difficult to do (e.g. less than 5% participants completed the questionnaire prospectively in a study by Leung *et al*.^29^). Two most common modes of data collection are self-reporting (in form of diary, for example) and interviewer-led (the interviewer may record information in a diary for the subject), typically collecting information retrospectively.

A possible way to minimize recall bias is to perform interviews on the day of reporting. Comparing results from such an interview to purely self-reported data allows assessment of the bias in studies that rely entirely on self-reporting without any memory aids. Such studies are rare and to our knowledge none assess consistency between self-reporting studies and telephone interviews. Another gap we identified involves characterizing and measuring human-animal contact. We identified a single study that considers human-human and human-animal contact patterns in Belgium^35^. However, detailed contact characteristics such as human-human contact duration, number of contact settings, human-to-animal touching duration, and contact matrix were not reported.

Our current study has three aims: (i) quantify local human-human (H-H) and human-animal (H-A) contacts in an urban setting in China, (ii) explore the interaction between H-H and H-A contact, and (iii) assess consistency between two different modes of data-collection, self-reporting and telephone interview-led studies.

## Methods

### Study site

The survey was carried out between December 2017 and May 2018 in Shanghai, a city in the southeast of China (Supplementary Fig. S1). The central urban districts of Shanghai were selected as our study sites, representing typical urban areas with high income and high population density (Supplementary Fig. S2). The 7 central urban districts are defined as areas within the outer ring road of Shanghai^36^, comprising a total population size of 7.3 million and an area of 289 square kilometers.

### Sampling

We sampled forty neighborhoods using multi-stage stratified sampling proportional to population size. A convenience sample of 25 households per neighborhood was selected so as to be broadly representative of the whole population of the neighborhood in terms of geographical distribution (Supplementary Fig. S3). One person per household was invited to participate in our study until we met our predefined target sizes equally balanced by age group and gender (Supplementary Text S1 for sampling details).

Our target sample size was 1000. Individuals of all ages who had been living in the selected neighborhoods for longer than two weeks and did not intend to move from Shanghai in the following two weeks were considered eligible for inclusion in the study (Supplementary Text S1 for population details).

### Data collection

Participants were asked to complete a questionnaire on their contact behavior for a randomly assigned day of the week. The questionnaire (see Supplementary Text S13) consisted of three sections, including general information, human-human and human-animal contact on the assigned day. General information comprised respondent demographics, household composition, daily travel, and daily contact. In the H-H contact section, in line with POLYMOD^10^, H-H contact was defined as either, (1) a two-way conversation with three or more words in the physical presence of another person (conversational contact), or (2) physical skin-to-skin contact (e.g. a handshake, hug, kiss or contact sports). Participants were requested to record each contact they made on the assigned day (during the 24 hours before going to bed at the participants’ normal bedtime), including the age and sex of the contact, whether contact was conversational or physical, the contact duration, the contact setting (e.g. home, work, etc.) and the frequency with which the respondent usually contacts the individual (e.g. daily, weekly, etc.).

In the H-A contact section, participants were first requested to record the species and the number of animals they owned. Animal ownership was defined as having an animal in the household in which they spend the majority of their time (i.e. living together in the house) as an alternative to touching, given that both are proxies for events that could lead to animal-human transmission. We only considered physical contact with animals, where contact was defined as touching at least one living animal. Participants were asked to record every type of animal they contacted on the assigned day, including the species, the number of animals, the contact duration, the contact setting, and the frequency with which they usually contacted the animal. At the end of the questionnaire, participants were also asked to report whether or not their assigned day was a typical one, as the factor may have affected their social contact patterns.

Large numbers of contacts are difficult to record individually, which can lead to under-reporting. We define “group contact” as contact with a group at least 20 individuals (e.g. as may be experienced by students, doctors, or those participating in a social event where one meets a lot of people). Contacts reported as individuals (i.e., not group contact) were termed “individual contact”. The questionnaire allowed participants to report a maximum number of 40 individual contacts.

Regarding the mode of data collection, there were two options for participants to choose freely: self-reporting and telephone interview. Participants were encouraged to select the telephone interview by answering a phone call from our trained investigator before their going to bed and recounting their contacts on the assigned day (whereby the investigator would complete the diary on their behalf). Alternatively, they could personally fill in a paper-based questionnaire (with attached instruction sheet). Participants who chose to self-report were encouraged to record their contacts prospectively (i.e. as they happened), and to complete the rest of the questionnaire before going to bed on the assigned day (Supplementary Text S2 for data collection details). Here we assumed that going to bed at night is sufficiently close to the end of the day and the end of the day’s social activities. Accordingly, the time period recorded was the 24 hours before going to bed.

### Statistical analysis

#### Demographic, travel and temporal factors

Four explanatory factors were considered when describing human social contact, animal ownership and contact. (1) Demographic and socioeconomic characteristics of the participant, specifically gender, age, work type, education level, individual income, household size, and number of years of living in Shanghai. (2) Whether participants travelled daily out of the subdistrict, or only occasionally. (3) Whether the reported contact day was a weekday or at a weekend, and whether the day was considered a typical day by the participant (had a similar life pattern to that of at least four days per week). Finally, (4) other survey-related factors included the mode of data collection (self-reporting or telephone interview), and population density level of the subdistrict (low: under 20,000 people per km^2^, moderate: 20,001–50,000 people per km^2^, high: over 50,001 people per km^2^).

To test for a difference in contact patterns under two different modes of data collection, we first applied propensity score matching to reduce confounding. The propensity scores were estimated for each participant using logistic regression, with the mode of data collection as response variable and other factors mentioned above as independent variables (“MatchIt” package in R 3.5.0).

#### Characteristics of human-human and human-animal contact patterns

Given the matched sample, we used weighted generalized additive mixed models (GAMMs) to assess the effect of the mode of data collection on the number of contacts, the number of individual contacts, the probability of reporting group contact, the contact duration in hours and the number of contact settings, whilst controlling for the animal ownership and other covariates mentioned above. Of all the models, we fitted penalized splines to explore potential nonlinear relationships of continuous participant age to the response variables, as it could be significant as found in  a study by Kwok *et al*.^31^. The sampling structure of our study allowed us to include the random intercept effect in our models to consider the proportion of variance in response variables attributable to intra- and inter-district variation. Estimating of the contact duration and the number of settings is provided in Supplementary Text S3. Similarly, we used the generalized additive mixed models to model the probability of reporting animal ownership and animal contact. Penalized splines were used to explore potential nonlinear relationships of continuous participant age and specially the number of H-H contacts to the response variables. Model details and variables selection are provided in Supplementary Text S4.

#### Human-human and human-animal contact matrix

We established 17 age classes (0–35 m, 3–6 y, 7–9 y, 10–14 y, 15–19 y, 20–24 y, 25–29 y, 30–34 y, 35–39 y, 40–44 y, 45–49 y, 50–54 y, 55–59 y, 60–64 y, 65–69 y, 70–74 y, 75 y and over) to build our age-specific H-H contact matrix, aiming to estimate the age-specific contact rate per person per day (Supplementary Text S3 for the estimating of the age of contacts). An original, symmetric and population age-weighted contact matrix was built using the “socialmixr” package in R 3.5.0. We used the *q* indices, representing departures from proportionate mixing and ranging from zero (proportionate) to one (fully assortative), and the bootstrapped 95% confidence intervals to assess the degree of age assortativity^37^ (Supplementary Text S5). H-A contact matrices were built in a similar manner. The bivariate smoothing approach was used to further estimate the social contact matrix^10,38,39^ (Supplementary Text S6).

#### Hypothesis testing for mode of data collection

We used bootstrap method to test the null hypothesis that telephone interviewed respondents would report more contacts by computing the difference between human contact matrices from self-reporting and telephone interviewed respondents (Supplementary Text S7).

#### Ethical approval

The study was approved by the ethical review committee of the School of Public Health at Fudan University, Shanghai, China (Ref: 2018-01-0659S). All methods were performed in accordance with relevant guidelines and regulations. Informed consent was obtained from all subjects (from a parent/guardian if participant was below 18 years of age).

## Results

### Demographic characteristics of participants

Overall, 1000 individuals from 1000 households were recruited, of whom 965 individuals were included in the final analysis (Supplementary Fig. S4). There was no statistical difference in gender between our sample and the central urban areas of Shanghai population (Shanghai census in 2017), but a statistical difference was found in terms of age (Supplementary Table S1). We found no difference in the age distribution and sex of participants between the participants recruited and the effective participants (Supplementary Table S2).

### Characteristics of human-human and human-animal contact patterns

**Figure 1:**
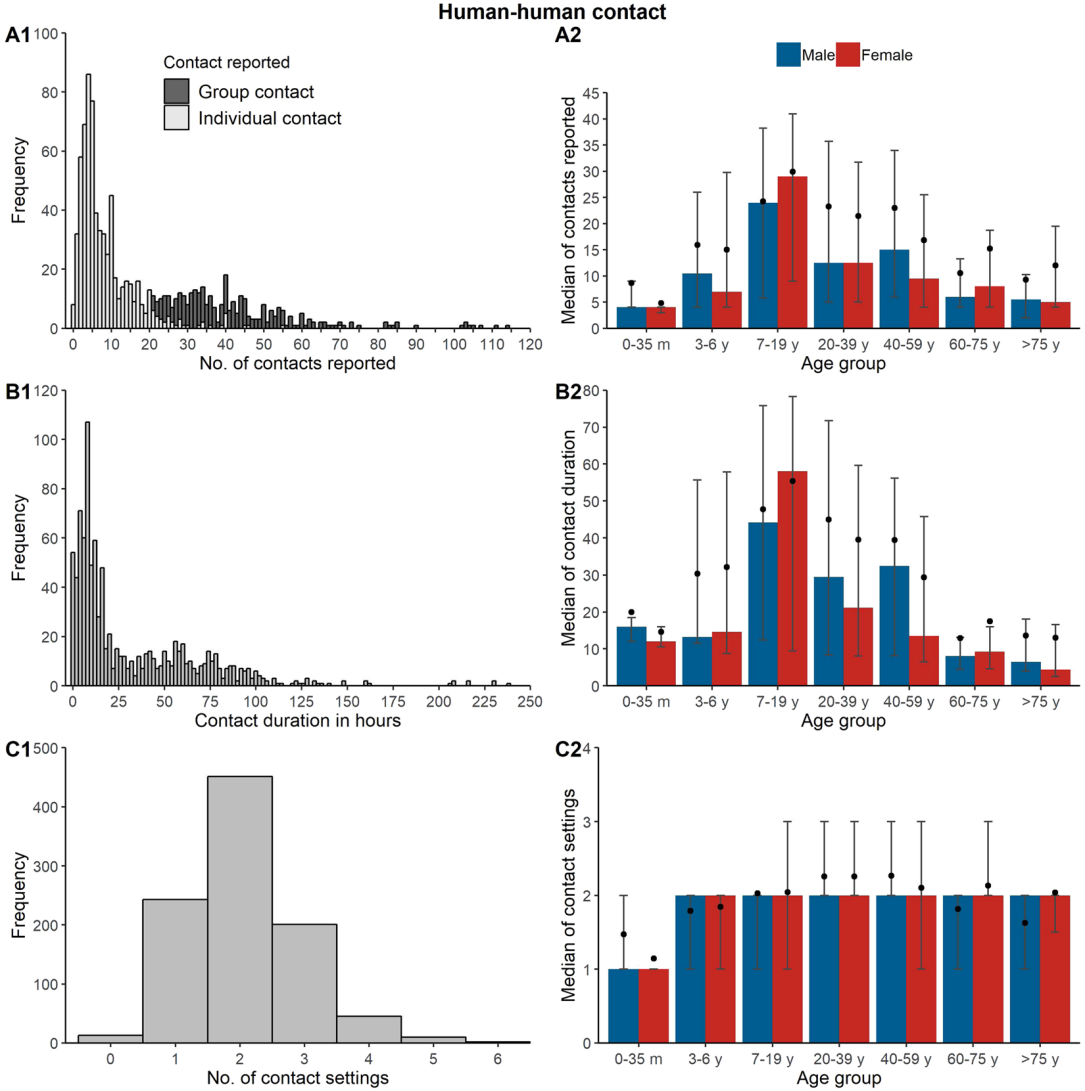
Distribution of human-human contact patterns (including the number of contacts, contact duration, and number of settings). The error bars in the right panels correspond to 25% and 75% quantiles, and the solid points correspond to the means.

We recorded 18,116 contacts in total from 474 male respondents and 491 female respondents, including 6,953 (38.4%) individual contacts, 10,240 (56.5%) contacts reported as group contacts, and 923 (5.1%) contacts that people estimate they had left out (Supplementary Text S13; questions 17–18.). The distribution of the number of contacts was highly right-skewed with a median of 10 contacts (Fig. 1 and Supplementary Table S3). Participants aged 7–19 years reported the greatest number of contacts. Similar distribution was found for the contact duration with a median of 15.7 hours (Supplementary Fig. S5 for the estimating of the duration of group contact). The number of contact settings was also right-skewed with a median of two settings, and participants aged younger than 3 years had the smallest number of settings. There was little difference in the number of contacts, duration, and the number of settings reported in terms of participant gender. More results about individual and group contacts are provided in Supplementary Text S8, Supplementary Fig. S6, and Supplementary Table S3. Physical contacts and other contact characteristics are described in Supplementary Figs S7 and S8.

**Figure 2:**
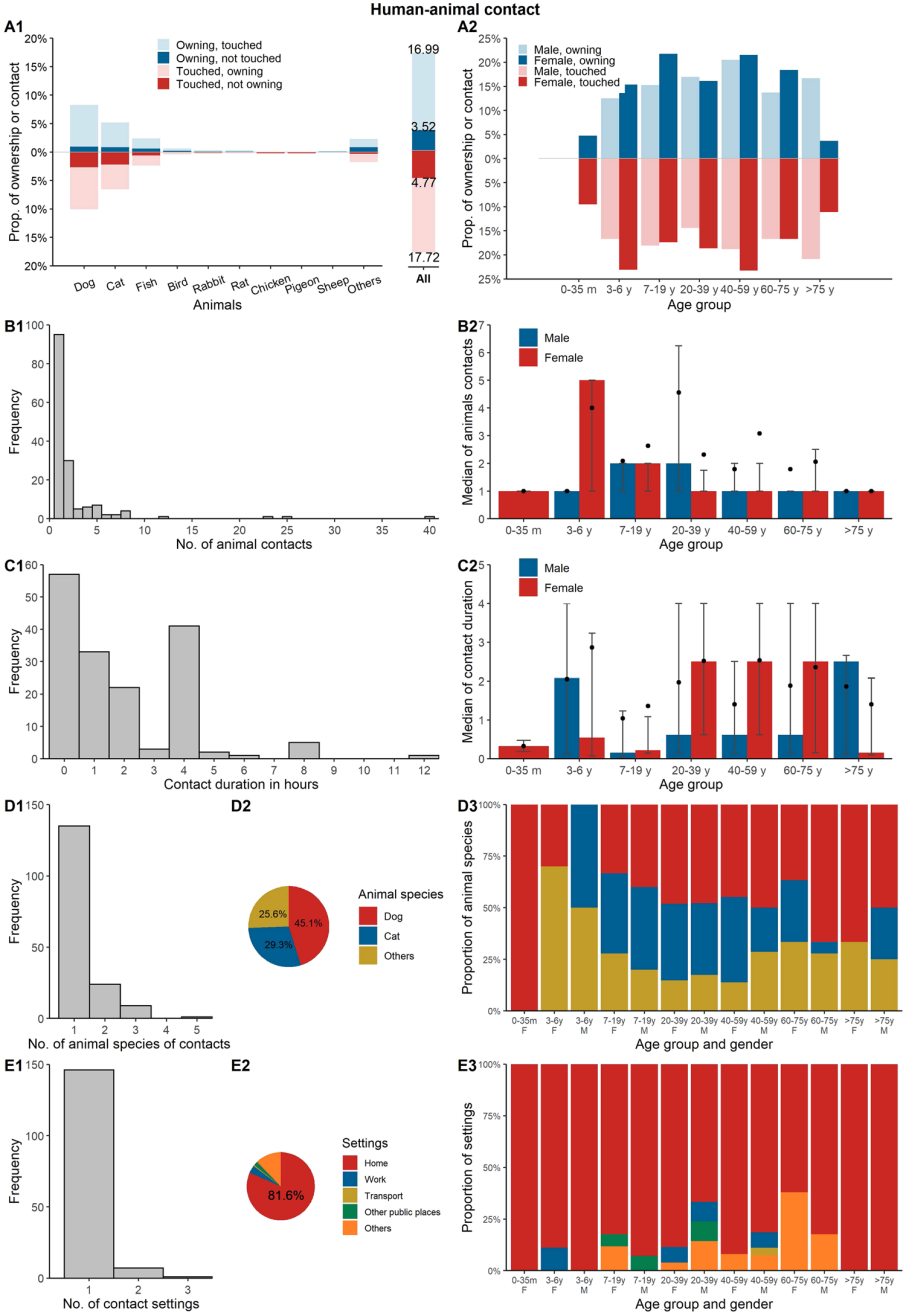
Distribution of human-animal contact patterns (including the probability of reporting animal ownership or animal contact, number of animal contacts, contact duration, number of species, and number of settings). (**A1**) Show the proportion of owners touching their own animals, the proportion of owned animals not touched, the proportion of touched animals that are their own and the proportion of touched animals not owned, respectively. All of the age groups and gender in the right panels refer to the humans. The error bars correspond to 25% and 75% quantiles, and the solid points correspond to the means.

Of the 965 respondents, 164 (17.0%) and 171 (17.7%), respectively, owned and had contact with at least one animal. Most of the reported animals were companion animals: 148 (15.3%) and 159 (16.5%) participants owned and had contact with pets (not including poultry or livestock), respectively, and most of which were dogs and cats. None of the participants reported owning poultry and only 0.2% of them had a contact with poultry. Only 0.1% of participants reported owning livestock, and none of them had contact with livestock (Supplementary Table S4). Only 3.52% of respondents owned animals but had no contact with animals on the assigned day, while 4.77% respondents had contact with animals that were not their own (Fig. 2A1). Respondents aged 0–35 months and those aged over 75 were least likely to own or have contact with animals, especially if they were male (Fig. 2A2). More results about H-A contact patterns are described in Supplementary Text S9, Supplementary Tables S4 and S5 and Supplementary Figs S9 and S10.

Of the 965 respondents, 194 (20.1%) reported owning or having contact with animal(s). The characteristics of such participants interacting with animals and their H-H contact patterns are shown in Supplementary Table S6 and Supplementary Fig. S11. Such participants had more social contacts (median: 16.5) and longer contact duration (median: 17.4), especially for children aged 3–6 and males aged 20–59, which motivated us to explore the interaction of H-H and H-A contact patterns.

### Factors associated with human-human and human-animal contact patterns

Participants were matched for potential explanatory factors associated with number of contacts using propensity score matching (Supplementary Table S7, Supplementary Fig. S12), resulting in 772 matched respondents (386 for each mode of data collection). Using matched data, the median of total contacts, contact duration and settings in H-H contact were 10, 15.2, and 2, respectively (Supplementary Table S8). The distribution of contact characteristics showed similar patterns as those before matching (Supplementary Fig. S13).

**Figure 3:**
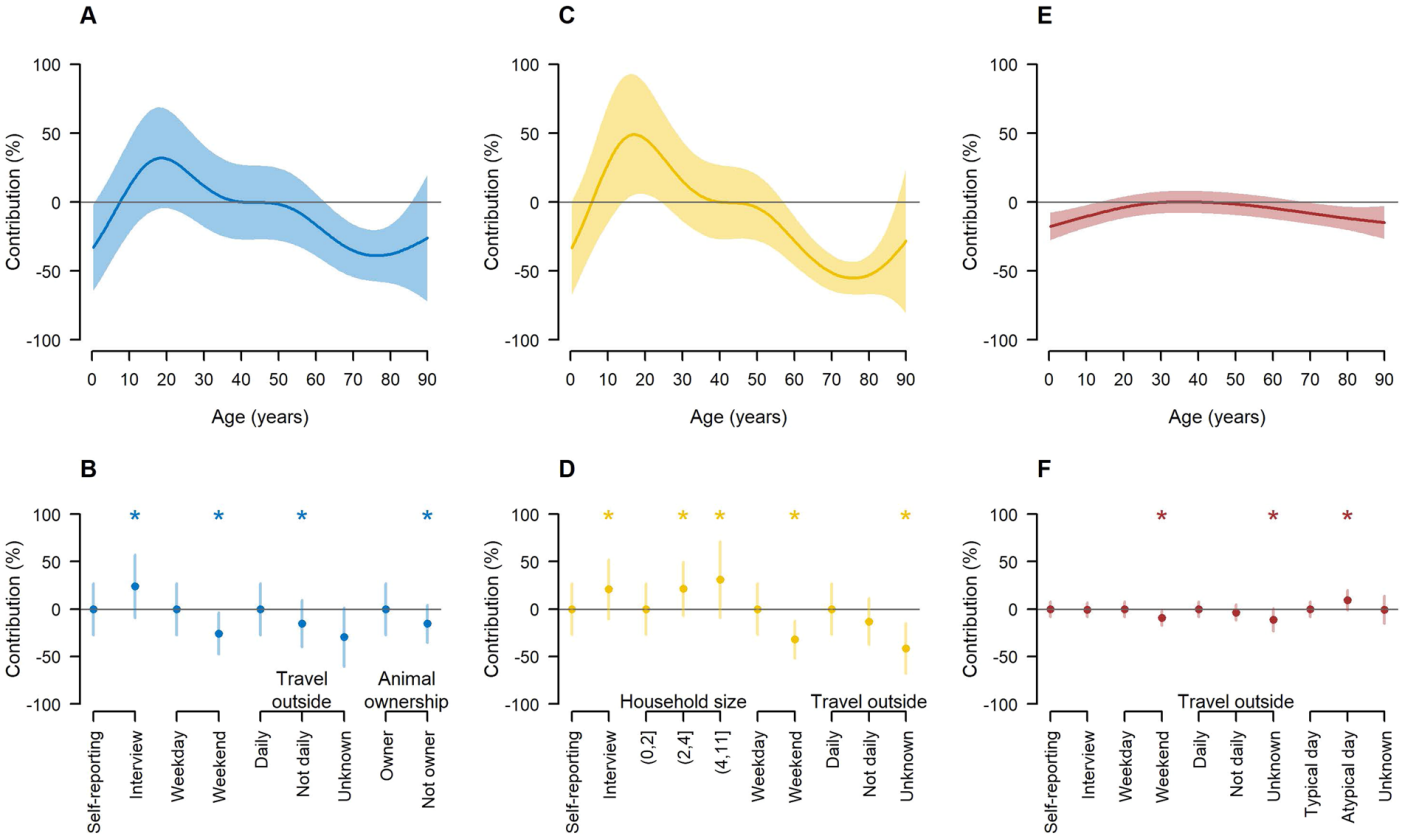
Estimates of percentage contribution of variables in human-human regression models. (**A**,**B**) Correspond to the predicted number of contacts; (**C**,**D**) correspond to the contact duration; (**E**,**F**) correspond to the number of contact settings. The predicted values are relative to a 40 years old person with other characteristics referring to the first level of each factor. 95% Confidence intervals are denoted by a shaded region as (**A**,**C**,**E**), or error bars as (**B**,**D**,**F**). Significant covariates of the regression models (at the 5% level) are denoted by stars.

For the H-H contacts, we used matched data to fit GAMMs (Fig. 3, Supplementary Table S9). In the final selected models, a significantly greater number of contacts was associated with telephone interview. Workdays, daily out-of-subdistrict travel, and animal owners were associated with a significantly greater number of contacts than weekends, not daily out-of-subdistrict travel, and not animal owners. We found a significant nonlinear association between the number of contacts and the participant age, with the largest number of contacts in those aged 18. Telephone interview was associated with a significantly longer contact duration. Living in a household with more than 2 people and workdays were associated with a significantly longer contact duration. A similar nonlinear relationship was found between contact duration and participant age. We found no significant association between the number of settings and modes of data collection. Workdays and atypical days were associated with a significantly greater number of contact settings. The number of contact settings was also significantly associated nonlinearly with participant age, with a greatest number associated with ages 30–50 and a decline below or above that. More results about individual and group contact are given in Supplementary Fig. S14, Supplementary Table S9, and Supplementary Text S8.

**Figure 4:**
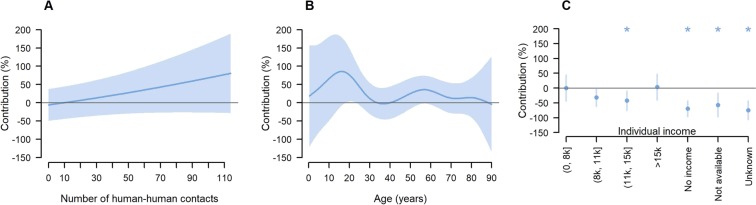
Estimates of percentage contribution of variables in the human-animal contact regression model. The predicted values are relative to a 40 years old person reporting the annual income of (0, 8000], and making contacts with 10 people. 95% confidence intervals are denoted by a shaded region as (**A**,**B**), or error bars as (**C**). Significant covariates of the regression models (at the 5% level) are denoted by stars.

For the H-A contacts, the number of social contacts was positively associated with the probability of having contact with animal(s), when controlling for the age and annual income of participants, with the largest probability associated with around 15 years and 60 years (Fig. 4, Supplementary Table S9). Participants reporting having no income was significantly associated with the lowest probability of animal contact, while participants reporting an annual income of over $15k had the highest probability of animal contact. Similar results were found for animal ownership (Supplementary Fig. S15).

### Human-human and human-animal contact matrix

**Figure 5:**
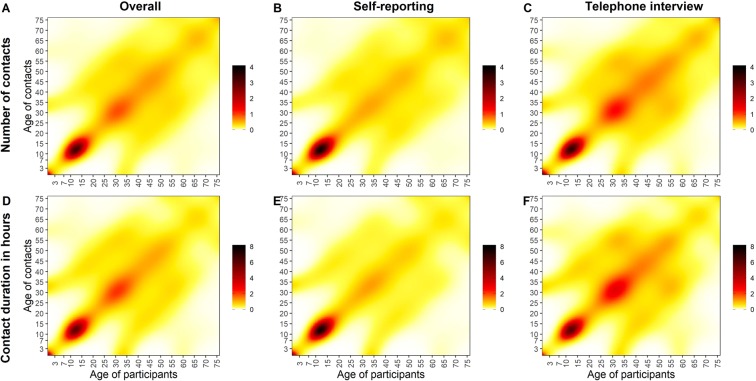
Smoothed human-human contact matrix. (**A**–**C**) Show the predicted average number of total contacts per day per participant and that stratified by different modes of data collection; (**D**–**F**) show the predicted average contact duration per day per participant and that stratified by different modes of data collection.

For the H-H contact (Supplementary Fig. S16), the degree of assortative mixing for the contact matrix of the number of contacts was calculated as *q* = 0.716 (bootstrapped 95%CI: (0.613, 0.779), self-reporting: 0.749 (0.611, 0.832), telephone interview: 0.568 (0.393, 0.744)), showing that the matrix was strongly assortative. With the smoothed matrices (Fig. 5), the dominant feature was the strong diagonal element, indicating that participants in all age groups were inclined to mix assortatively by age, which was most pronounced in those aged 7–20, and least pronounced in those aged 25–55. Two parallel secondary diagonals starting around 30–40 for both contacts and participants were offset from the central diagonal likely capturing contacts that parents make with their children and vice versa. Compared to self-reporting, telephone interviewing was associated with a greater number of contacts for participants aged 20–60, but smaller for participants aged 3–20. The contact matrix of contact duration had a similar pattern to that of number of contacts. The estimating of the age of group contact is provided in Supplementary Fig. S5. Contact matrices stratified by different characteristics are shown in Supplementary Figs S17 and S18.

For the H-A contact (Supplementary Fig. S16), the number of animals owned or contacted with did not vary substantially across age groups, but participants aged 3–7, 15–35, and above 50 years had longer animal contact duration compared to other age groups. H-H and H-A contact matrices of reported contacts are showed in Supplementary Tables S10 and S11.

For the interaction between H-H and H-A contact, Supplementary Figs 19 and 20 showed the H-H contact matrices stratified by whether had animal ownership or animal contact. For the number of contacts, the contact matrix for participants not interacting with animals was more strongly assortative (*q* = 0.73) than that interacting with animals (*q* = 0.65), especially for those aged 7–20. But animal ownership or contact was associated with a greater number of contacts in those aged 25–55. The contact matrix of contact duration had a similar pattern to that of number of contacts. H-H contact matrices for such participants interacting with animals are shown in Supplementary Table S12.

### Hypothesis testing for mode of data collection

We compared the age-specific contact matrices of observed contacts via self-reporting and telephone interview. No significant difference was found for most of the elements (Supplementary Figs S21 and S22). For participants aged 20–60, telephone interviewing was associated with greater number of contacts, which was consistent with what we found in the smoothed contact matrices (Fig. 5).

In addition to comparing the similarity of contact matrices for the two modes of data collection, we evaluated differences in respondent self-ratings about the quality of data collection. We found that the telephone interview participants rated the quality of their responses across a number of dimensions higher than self-reporting participants (Supplementary Text S10, Supplementary Table S13).

## Discussion

We have conducted the contact study of H-H and H-A contacts in Shanghai City, a high-income densely populated megacity in Eastern China. We recorded a mean of 18.7 contacts, 32.1 contact hours, and 2.1 contact settings per participant, where the average number of contacts by telephone interview (19.3) was significantly higher than that recorded by self-reporting (18.0). The H-A contact (especially poultry) was rare, although the probability of owning or having contact with animal(s) was positively associated with the number of social contacts.

Our study is the first contact study in any part of mainland China apart from Guangdong. Nevertheless, it is in broad agreement with other studies in China as well as in the wider region. The average number of contacts per person in our study was in broad agreement with studies from Guangdong^28^ (18.56) and Hong Kong^40^ (18.0–18.6), while it was larger than those from Taiwan^27^ (12.5) and Japan^26^ (15.3) (Supplementary Table S14). We identified three studies in Hong Kong presented different results (18.0–18.6 in a study by Kucharski *et al*.^40^, 12.5 in a study by Kwok *et al*.^31^, and 8.1 in a study by Leung *et al*.^29^). Kucharski *et al*. included group contacts to capture large numbers of contacts. Kwok *et al*. also permitted the reporting of group contacts, but it was a retrospective design, where the interview day was assigned within 4 days after the reporting day. Leung *et al*. did not define group contacts, possibly contributing to underreporting. The number of contact settings in our study was slightly lower than the 2.9 contact locations in the Kwok *et al*. study, probably because the Kwok *et al*. study measured the number of geographical locations but we measured the number of contact settings that could not accurately determine the total number of different locations the participant encountered people at. We observe similar age-assortativity to many studies in other countries or regions, but with higher number of contacts made among 20–60 years (Supplementary Fig. S23). Similar to a large number of other studies^41^, we also identified age, day type (e.g., weekday/weekend), and household size as determinants of H-H contact patterns. In addition, we identified other factors having a significant influence on H-H contact pattern, such as whether the day was considered a typical day by the participant, animal ownership, and whether participants travelled daily out of the subdistrict, or only occasionally. The link between travel and contact patterns would provide additional insights on whether or not infection could be imported.

Our study collected details about important H-A contact patterns, including animal species, number of animals, contact duration, contact setting and contact frequency of contacts with different types of animals, explored the association between H-H and H-A contact patterns, and quantified the H-H contact patterns for such participants owning or touching animals. This is particularly important because only one other study^35^ has examined both H-H and H-A contacts. This study sampled respondents in Belgium, and the pattern of animal contacts differed substantially from ours. In particular, both animal ownership and animal contact were much higher in Belgium (ownership: 51% pets, 15% poultry and 5% livestock; touch: 46% pets, 2% poultry and 2% livestock) compared to Shanghai (15.3% pets, 0% poultry and 0.1% livestock) and 17.7% (16.5% pets, 0.2% poultry and 0% livestock). Although both studies were based in high-income urban settings, the lower animal contact in Shanghai (especially poultry), may stem from the closure of live poultry markets due to the spread of influenza A(H7N9) in Shanghai since 2013, and also from the differences in both cultural norms and population density in Europe compared to Asia. For instance, population density is 25,260 people per km^2^ in central urban areas of Shanghai compared to 6,790 people per km^2^ in Brussels. Both studies found that animal owners were likely to have more social contacts, and we still identified linear relationship between the number of social contacts with the probability of owning or touching animals. 18.4% of the animal contact in our study occurred out of the home setting, which may explain why more animal contacts were associated with more social contacts. From a socioeconomic perspective, participants reporting highest annual incomes (i.e. over $15k) had the highest probability of owning animals or touching with animals, after adjusting for participant age. We also quantified the H-H contact patterns, including the H-H contact matrices, for such participants interacting with animals, which provided important parameters to evaluate the potential for crossing the animal human barrier and the potential of outbreak in human populations.

Such information is important since Eastern China has been identified as hotspot for emergence of zoonotic infectious diseases^1^ and Shanghai has been one of the potential epidemic centers in China as the first case of influenza A(H7N9) was found in Shanghai^42^. Our study results suggest that data on animal contacts from other settings cannot be extrapolated to Asia. In particular, we find that there is low contact between humans and poultry in Shanghai. However, we also found that people with more animal contacts are likely to have more social contacts. The potential for crossing the animal human barrier is limited but once that is done, the potential for transmission is enormous. In addition, our work helps understand the transmission dynamics of diseases transmitted by dogs and cats, such as rabies. In China, 92% human deaths of rabies were caused by animal bites, where wounds from domesticated animals (dogs and cats) were responsible for more than half of the rabies cases^43^, and the east and south regions of China (including Shanghai) were more seriously affected compared with other regions^44^. Hence the human-animal contact patterns observed in this study would be very important evidence for further research on epidemic dynamics at the human-animal interface, animal control and vaccination programs, which could greatly impact public health.

Another important feature of our study is the use of multiple modes of data collection and questionnaire types. A few other studies have also used multiple modes and these have found important differences in responses when using different modes^10,14,45^. Several different modes of data collection used in previous studies included self-completed paper contact diaries^10,14,29,45^, electronic contact diaries^24^, online contact diaries^11,14,29^, and interview-led contact diaries^28,31^ (Supplementary Table S14). Perfect recall of all encounters is impossible, especially for short-duration or large-size encounters. Some studies have found electronic self-completed contact results to perform similarly to paper diaries^14^, while others have found more contacts are recorded using paper diaries^24,29^. Web-based questionnaires are very helpful in large-scale surveys, but are probably associated with lower response rates compared to paper-based questionnaires^41^. A recent systematic review^41^ has shown that interviews are typically better at capturing all contacts compared to self-reporting. In our study, we found that more contacts are recorded via telephone interview at the end of the reporting day compared to self-reporting, which is likely to result from less recall bias. However, there has not been any evidence to answer whether interviews conducted face-to-face or via telephone yield different results. Based on our experience in this survey, we believe face-to-face interview will provide more accurate measurements, though at the expense of greater human and material resources than telephone interview. We considered this kind of telephone interview-led method as a potential improvement of the design of future contact surveys, despite of the following limitations. First, participants got called at a pre-agreed time period after dinner and before going to bed (i.e. close to the end of the survey day rather than the real end of the day); different calling times may possibly interfere with the recording of social contacts. While we balanced the two modes of data collection via propensity score matching, there may still be other factors influencing a participants’ choice of interview mode that we didn’t measure. For example, individuals who are busy and have many social activities may be more likely to want an interview by telephone rather than self-reporting, in the belief that there would be less time burden for participation. Finally, similarly to other forms of interviews, telephone interviews could introduce interviewer bias (due to different interviewers), despite standardized training of interviewers. We identified heterogeneity in reported rates between different interviewers, though sensitivity analysis suggests individual interviewers have little influence on our primary contact rate estimates (Supplementary Text S12).

Besides collection of detailed data about animal contacts, our study also contains a number of other important strengths. Firstly, we used a spatial sampling design, sampling from the Chinese administration units (district-subdistrict-neighborhood) so as to represent the population of the central urban of Shanghai. This study also proposed a novel data collection method and evaluated the ability to record contact behavior via self-reporting compared to interview-led methods (and thus, was able to compare these methods). A telephone interview at the end of the reporting day by a trained interviewer elicited a greater number of contacts than self-reporting; therefore, we believe this methodology can substantially help to reduce recall bias.

There are some limitations to this study. We asked respondents about the contact setting rather than the actual geographical locations where the contact occurred. Similar to many other contact studies^10,12,13,18,21,28,31,35,40^, we allowed respondents to group large numbers of contacts found in the same location in a single “group contact”, but we only asked about the size of the group and the age range of the group contacts without other contact details, such as gender, age, physicality, setting, and duration of each contact. Thus, we used statistical models to estimate the age and duration of each group contact. The number of group contacts that could be reported was limited to one; similarly, the questionnaire limited the reporting of individual contacts to 40. This may have led to some censoring and under-reporting of contacts, though the majority of participants reported fewer than 40 contacts (Supplementary Fig. S6).

The human-human contact in this megacity is very frequent but that contact with animals, and poultry in particular, is surprisingly low. This would imply that the most likely source for a zoonosis lies outside in the more rural areas. We propose that a separate study with a similar design should look at this.

## Supplementary information

Supplementary figures

Supplementary tables

Supplementary text

## Data Availability

The data collected as part of this survey will be made available to the scientific community via the zenodo data repository as part of a social contact data collection initiative www.socialcontactdata.org.
