# Supplementary material for: Patterns of human social contact and contact with animals in Shanghai, China
Source: Sci Rep. 2019 Oct 22;9:15141. doi: 10.1038/s41598-019-51609-8 (PMC6805924; doi:10.1038/s41598-019-51609-8)
Supplement: Supplementary file 1 — Supplementary figures [file 41598_2019_51609_MOESM1_ESM.pdf]

Patterns of human social contact and contact with animals in Shanghai, China

Juanjuan Zhang[1], Petra Klepac[2], Jonathan M. Read[3], Alicia Rosello[2], Xiling Wang[1], Shengjie Lai[1,4,5], Meng Li[1], Yujian Song[1], Qingzhen Wei[1], Hao Jiang[1], Juan Yang[1], Henry Lynn[1], Stefan Flasche[2], Mark Jit[2,6,7], Hongjie Yu[1*]

[1]*School of Public Health, Fudan University, Key Laboratory of Public Health Safety, Ministry of Education, Shanghai, China*

[2]*Department of Infectious Disease Epidemiology, Faculty of Epidemiology and Public Health, London School of Hygiene and Tropical Medicine, London, UK.*

[3]*Centre for Health Informatics, Computation and Statistics, Lancaster Medical School, Lancaster University, Lancashire, UK.*

[4]*WorldPop, School of Geography and Environmental Science, University of Southampton, Southampton, UK*

[5]*Flowminder Foundation, Stockholm, Sweden*

[6]*Modelling and Economics Unit, Public Health England, London, UK*

[7]*School of Public Health, University of Hong Kong, Hong Kong, China*

*Corresponding author:

Professor Hongjie Yu

School of Public Health

Fudan University

Email: yhj@fudan.edu.cn

Supplementary Figure S1-S24

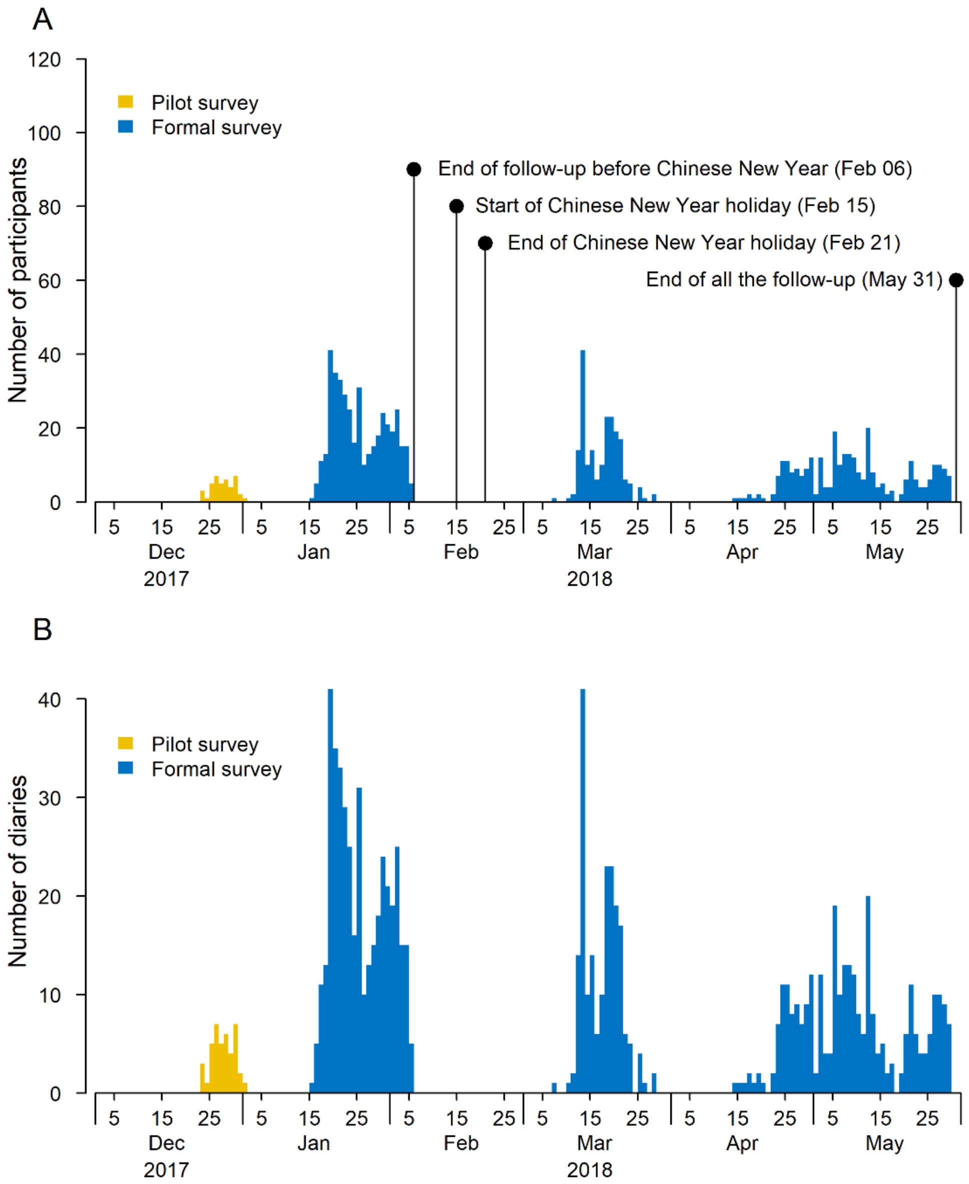

Figure S1. Timeline of the survey. (A) The recruitment date (B) and the date of contact diaries for participants, from Dec 23, 2017 to May 31, 2018. We excluded some days before and after the Chinese new year holidays so that we could measure the routine contact patterns.

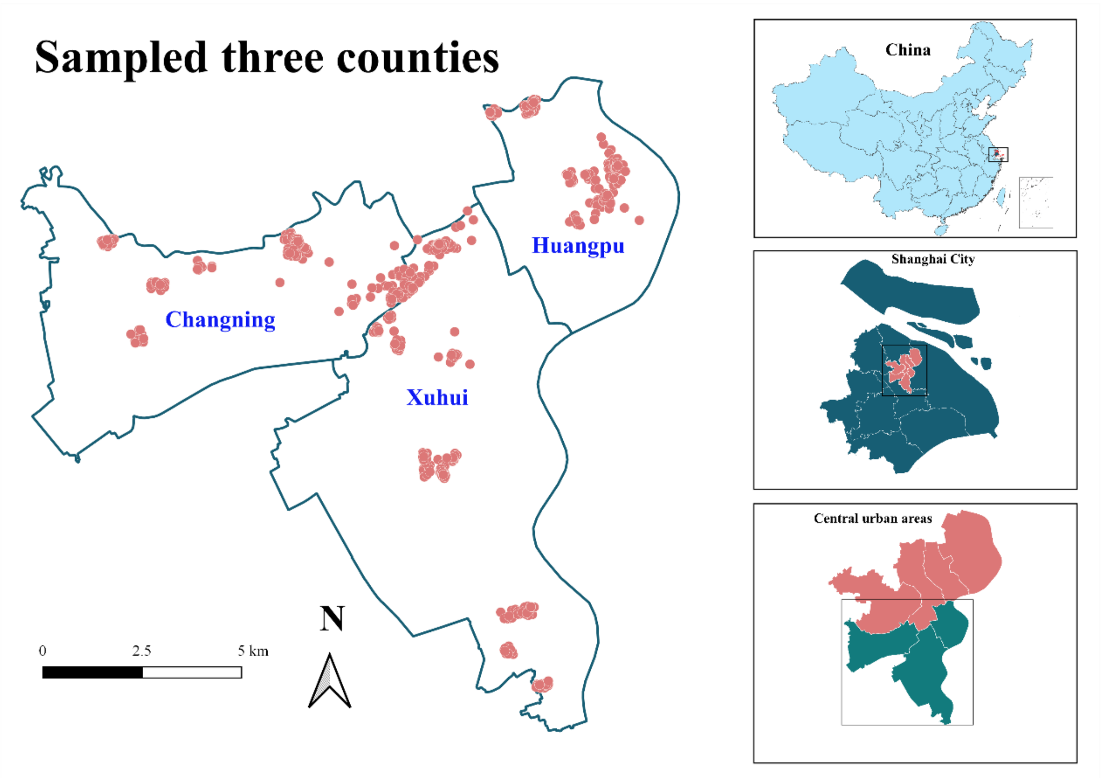

Figure S2. Three districts (counties) sampled and participants home locations in Shanghai.

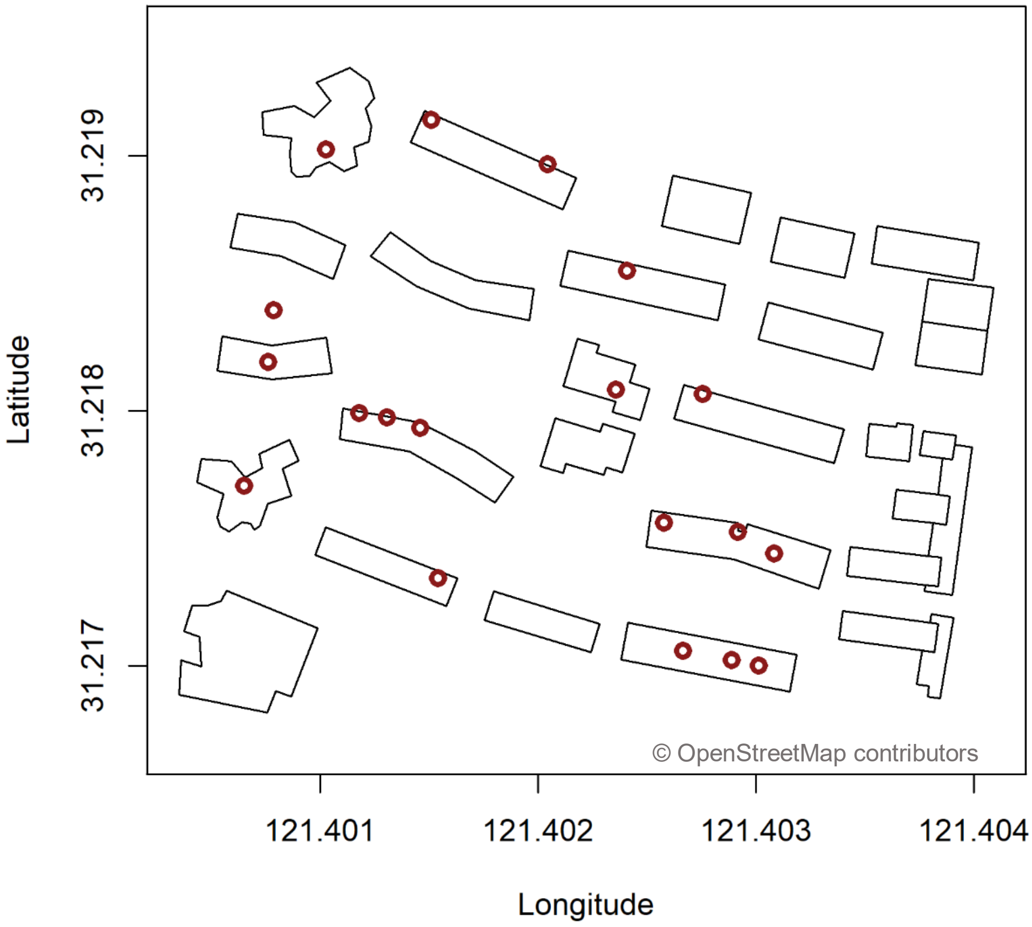

Figure S3. Home locations of participants sampled from one neighborhood for instance (neighborhood ID: 30304). Polygons and open circles represent buildings and home locations, respectively. This figure was built on OpenStreetMap. Map data is copyrighted "© OpenStreetMap contributors" and available under the Open Database License, and the cartography is licensed as CC BY-SA (https://www.openstreetmap.org/copyright).

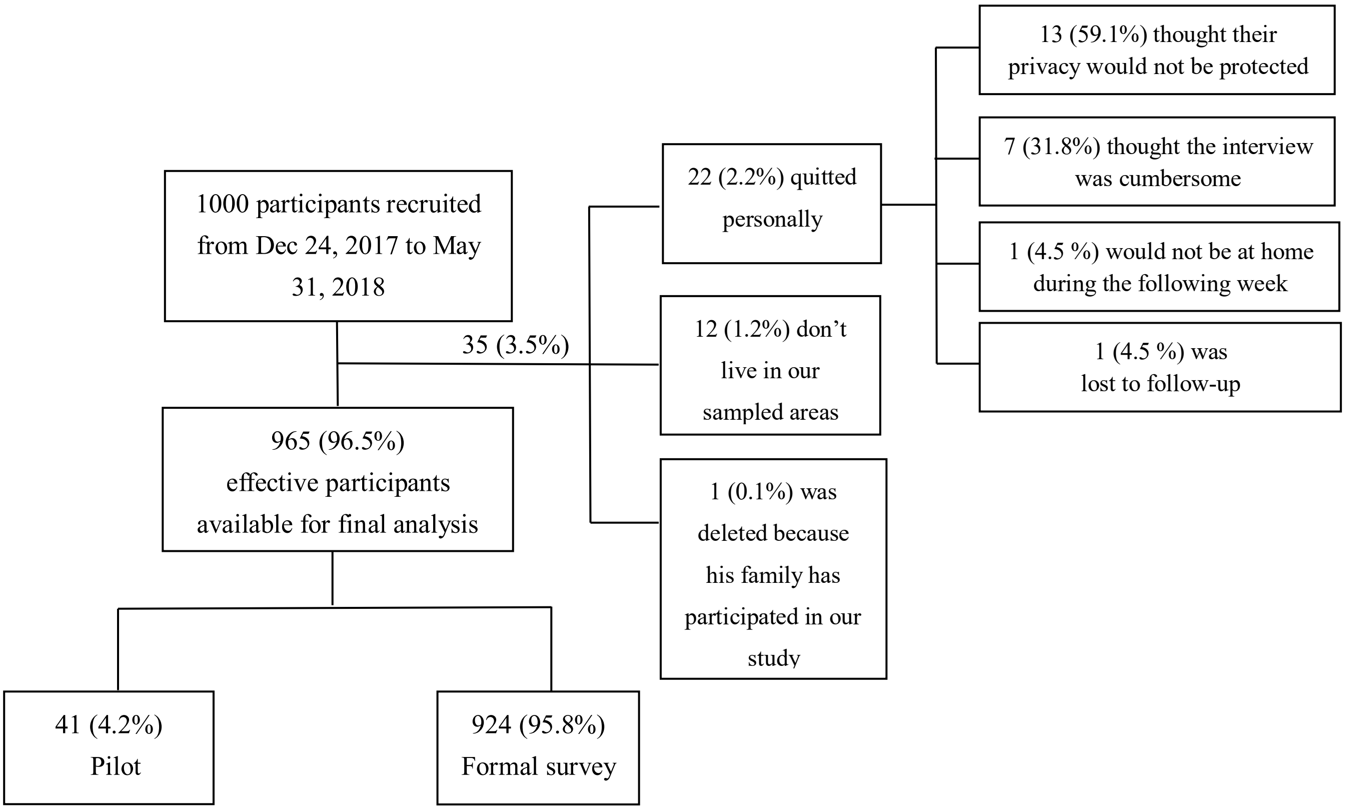

Figure S4. Flow chart from enrollment to analysis for the 1000 participants.

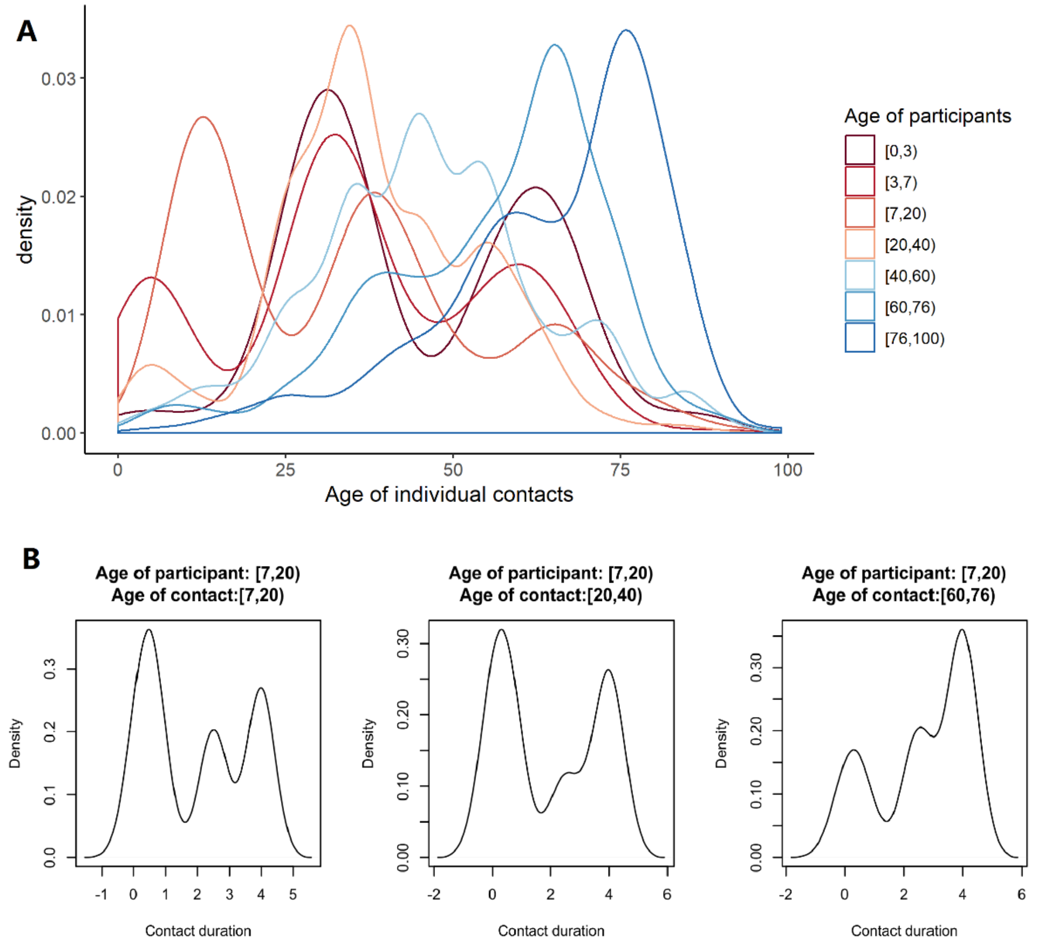

Figure S5. Model fitting for estimating the age and duration of each group contact. (A) the age of individual contacts, and (B) the individual contact durations between different age groups of participants and contacts. For example, three plots at the bottom panel showed the distributions of contact duration crossing age group [7, 20), [20, 40) and [60, 76). With classified seven age groups ([0, 3), [3, 7), [7, 20), [20, 40), [40, 60), [60, 76), and 76+), we had 49 fitted kernel density functions so that every group duration could be sampled from the corresponding kernel density functions.

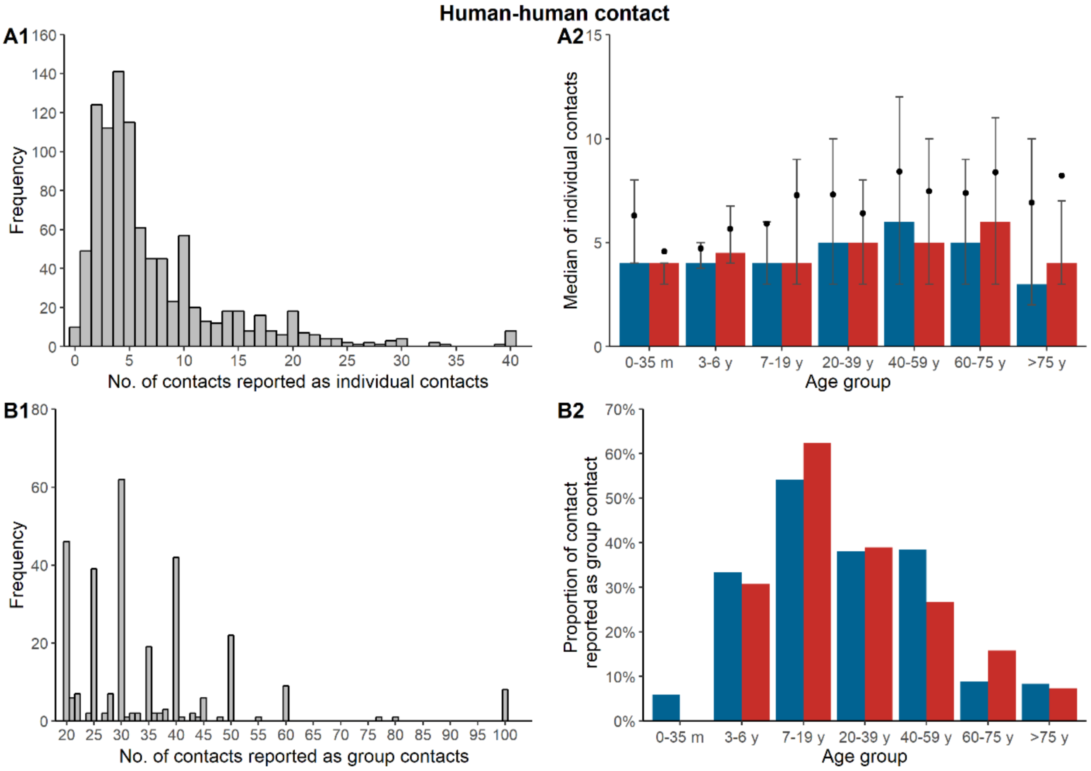

Figure S6. Distribution of H-H contact patterns. (A1) the number of individual contacts, (B1) the number of group contacts, and (A2-B2) corresponding distributions across different age groups and gender. The error bars in the right panels correspond to 25% and 75% quantiles, and the solid points correspond to the mean.

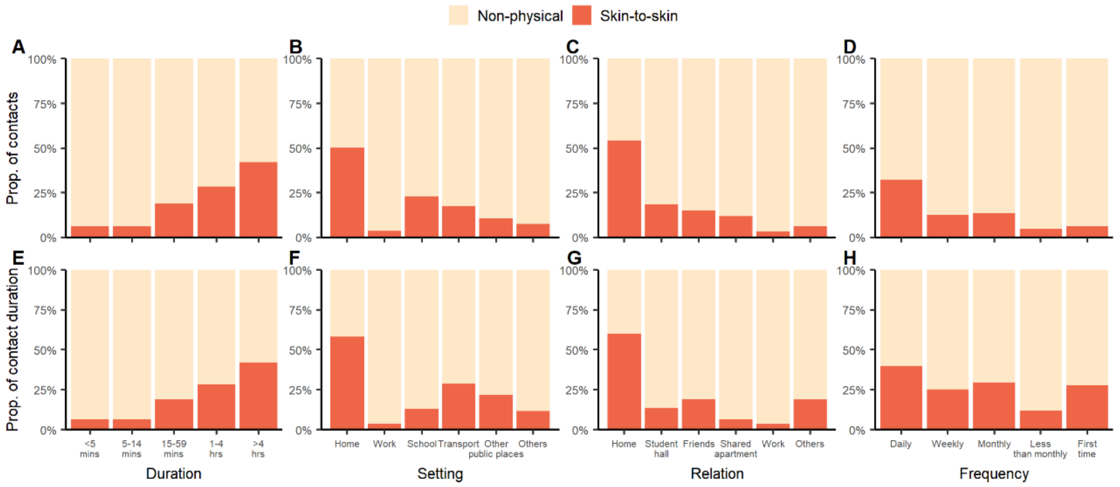

Figure S7. The proportion of reporting physical social contacts.

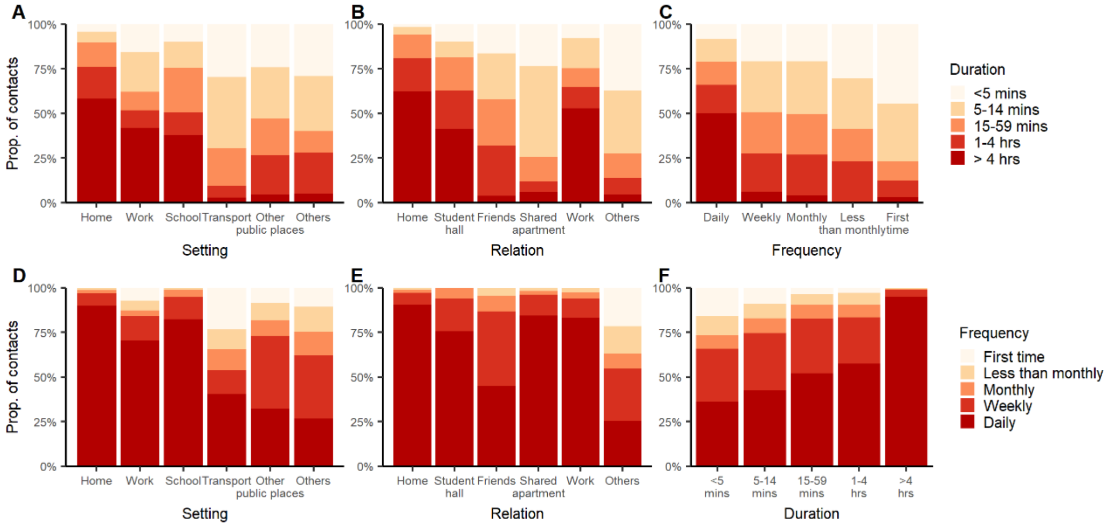

Figure S8. The relationship among the human contact setting, relation, frequency and the duration.

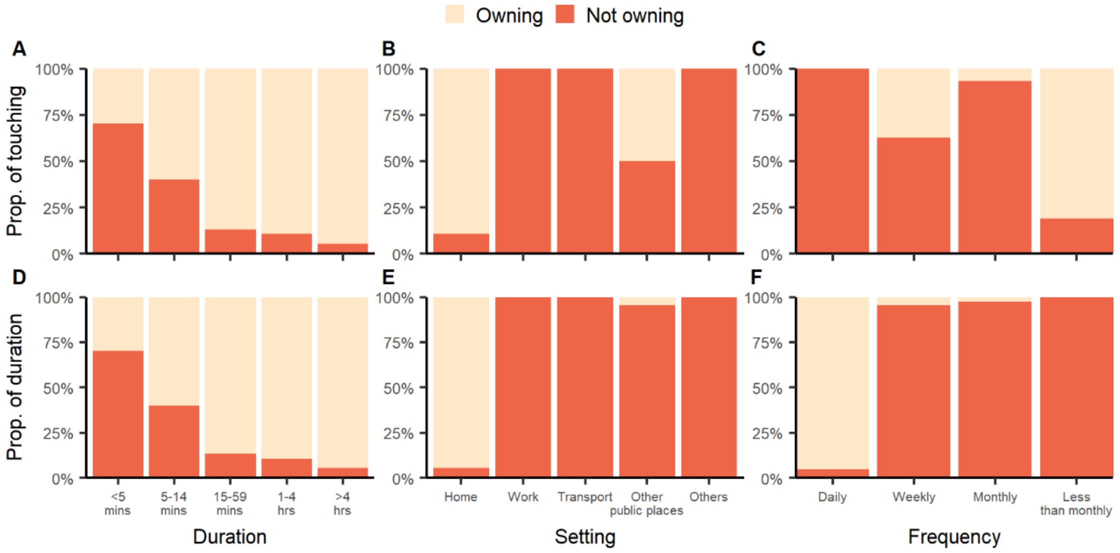

Figure S9. The proportion of having a contact with animals that don't belong to him/her.

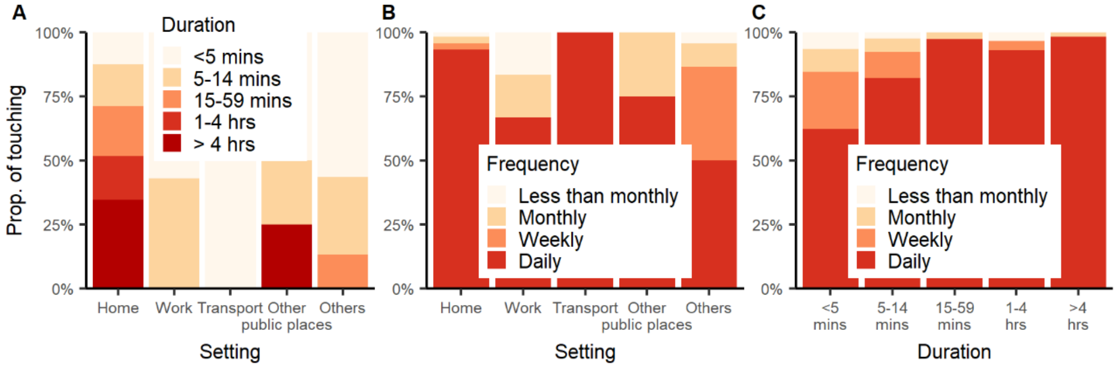

Figure S10. The relationship among the animal contact setting, frequency and the duration.

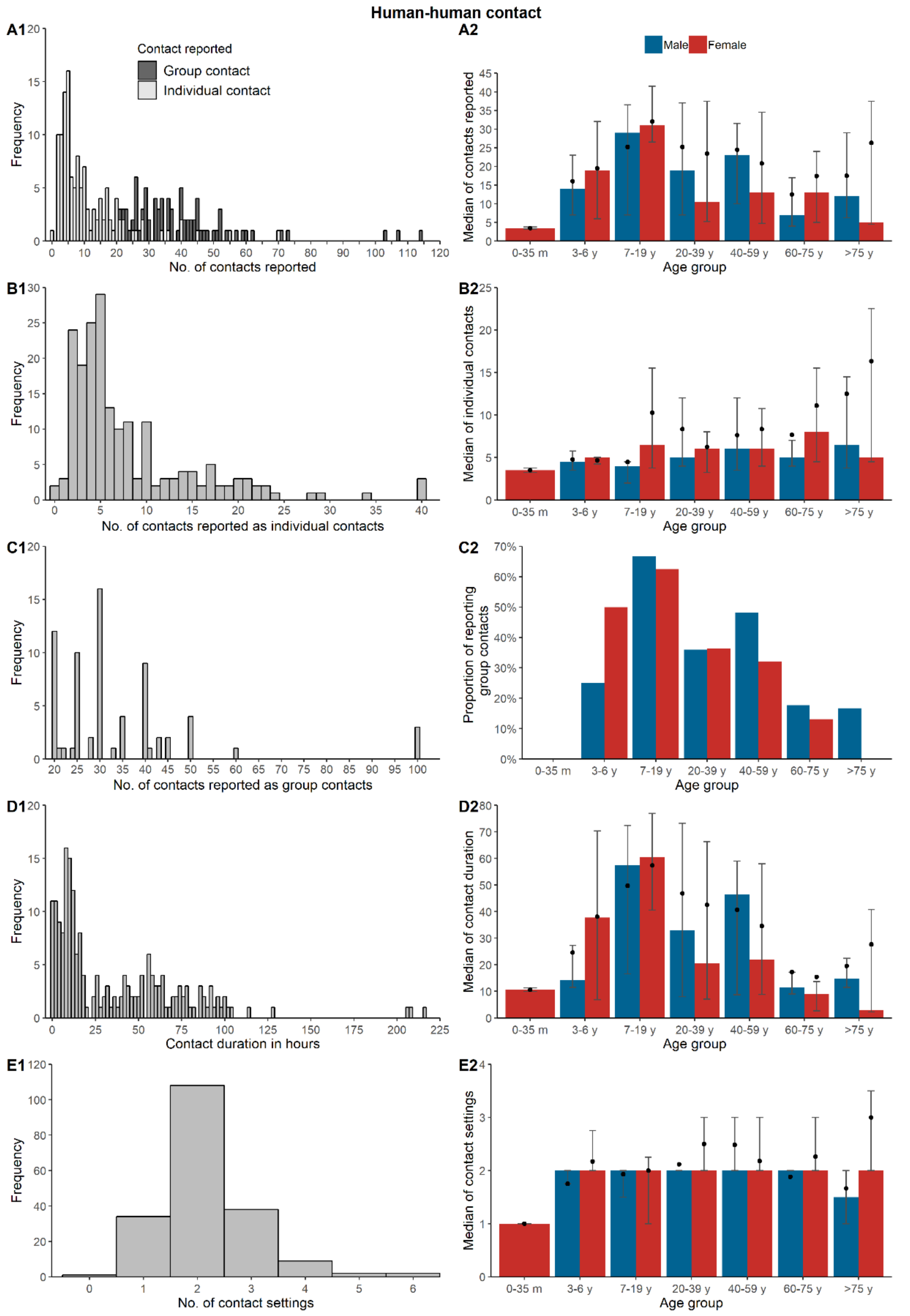

Figure S11. Distribution of human-human contact patterns of the participants owning animal or touching animals. (A1-E1) show the distributions of the number of total contacts, the number of individual contacts, the probability of reporting group contact, contact duration in hours, and the number of contact settings per person per day; (A2-E2) show the corresponding distributions across age groups and gender. The error bars in the right panels correspond to 25% and 75% quantiles, and the solid points correspond to the mean.

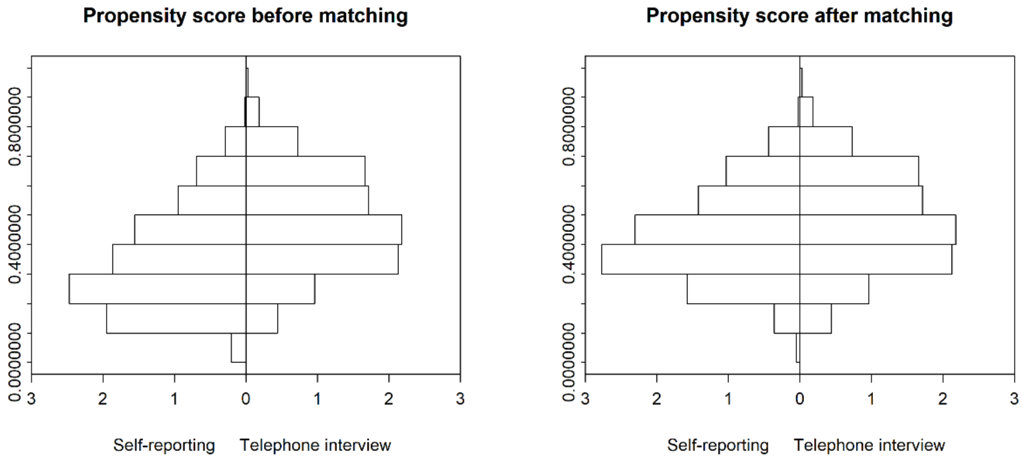

Figure S12. Propensity scores before and after matching.

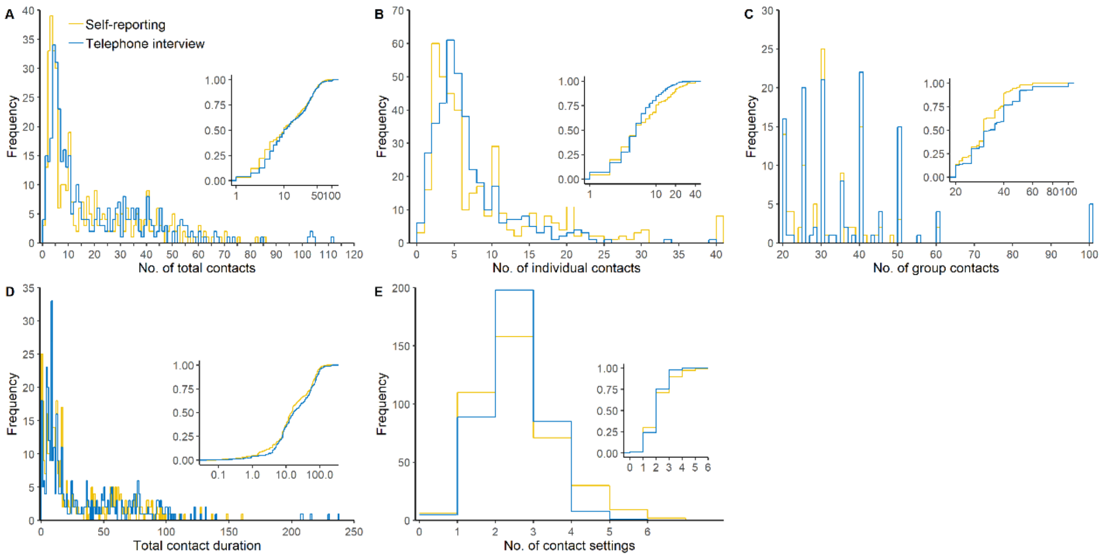

Figure S13. Distributions of (A) the total number of contacts, (B) the number of individual contacts, (C) the number of group contacts, (D) the total contact duration, and (E) the number of contact settings using propensity score matched data. Different modes of data collection are denoted by unique colors as shown in A-E. Insert plots show the corresponding cumulative probability distributions for each mode of data collection, color coded as for the main plots.

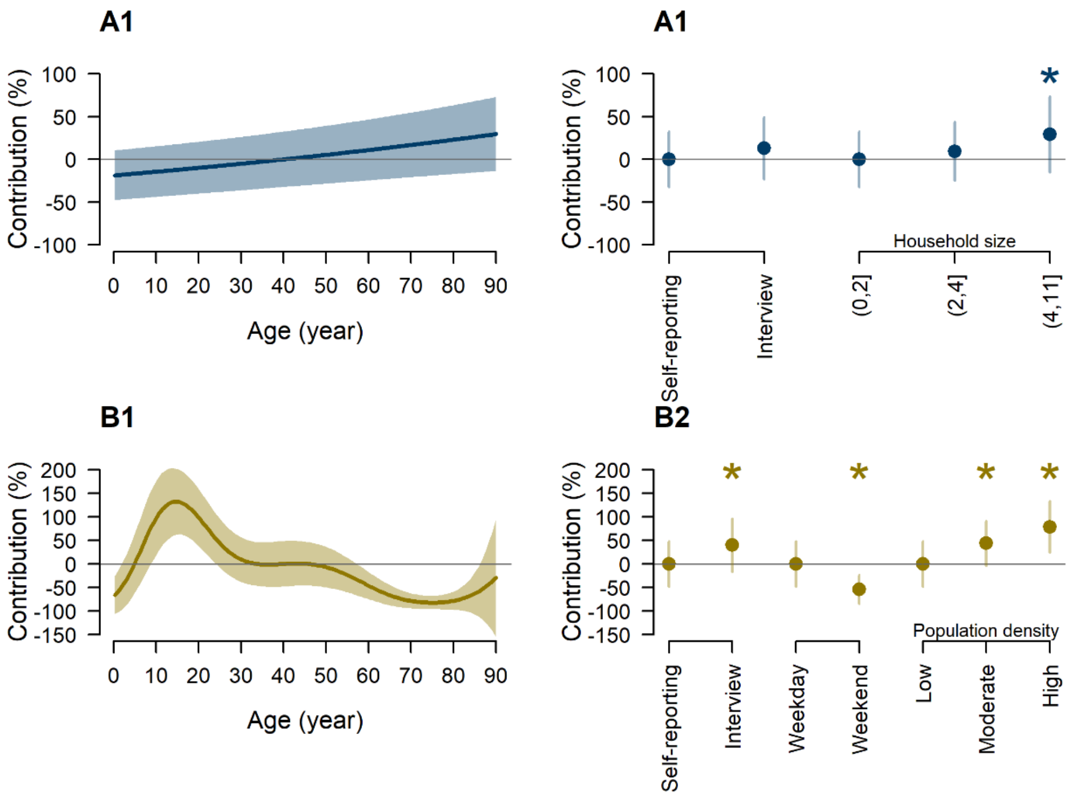

Figure S14. Estimates of percentage contribution of factors in human-human contact regression models. (A1 and A2) The predicted number of individual contacts, (B1 and B2) the predicted probability of reporting group contact.

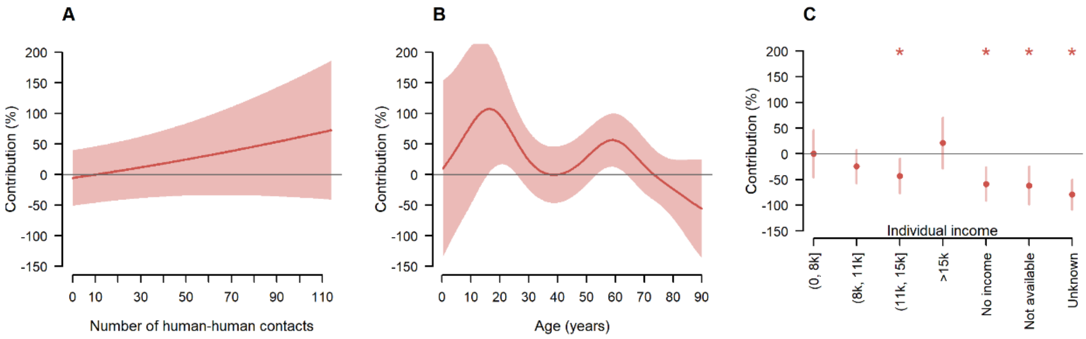

Figure S15. Estimates of percentage contribution of variables in the animal ownership regression model. The predicted values are relative to a 40 years old person reporting the annual income of (0, 8000], and making contacts with 10 people. 95% confidence intervals are denoted by a shaded region as A and B, or error bars as C. Significant covariates of the regression models (at the 5% level) are denoted by stars.

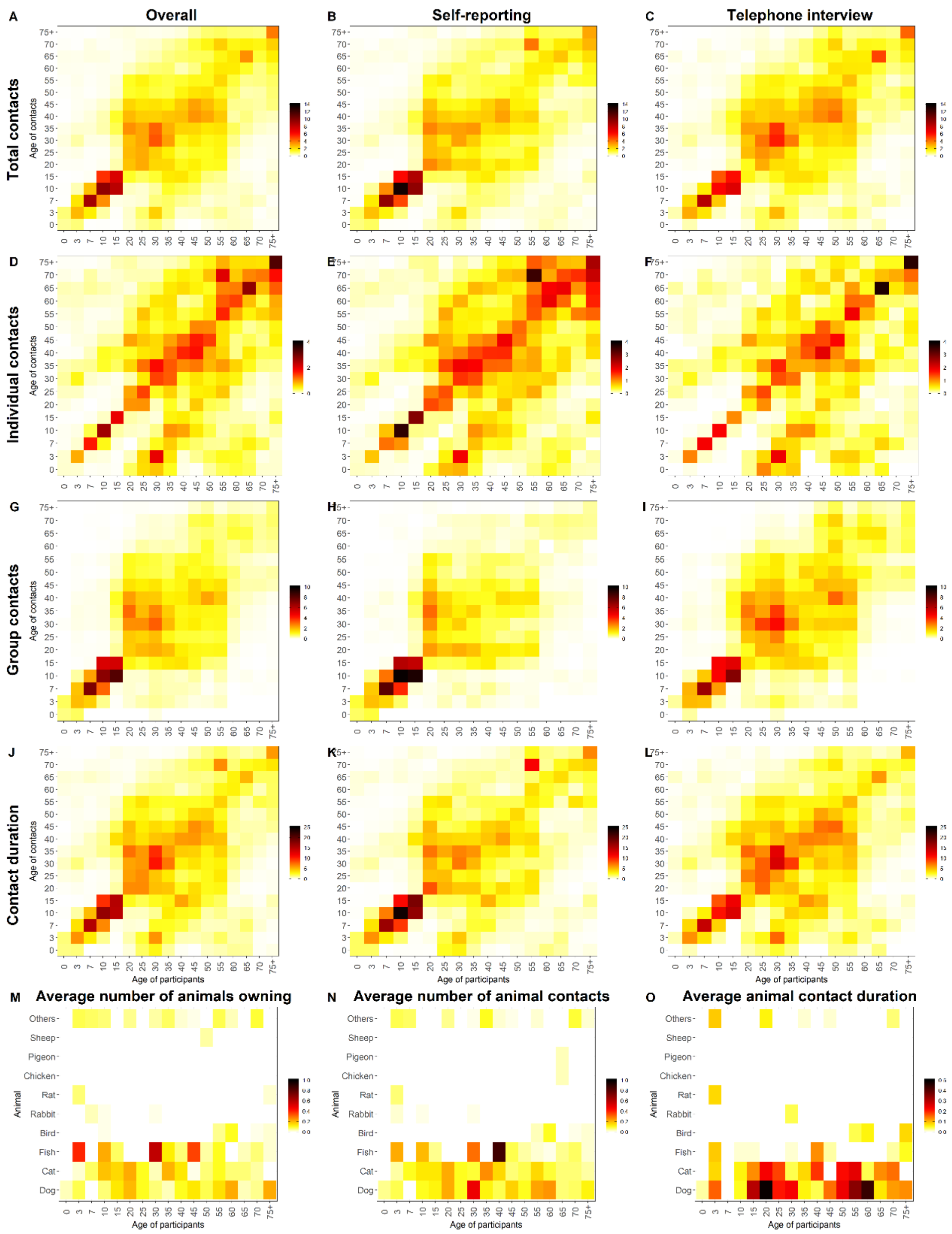

Figure S16. Weighted human-human contact matrices consisting of the average number of contacts, the number of individual contacts, the number of group contacts, and the contact duration per day per participant (overall: A/D/G/J, self-reporting: B/E/H/K, telephone interview: C/F/I/L), and human-animal contact matrix consisting of (M) the average number of animals owing per participant, (N) the average number of animal contacts per day per participant, and (O) the average contact duration in hours per day per participant.

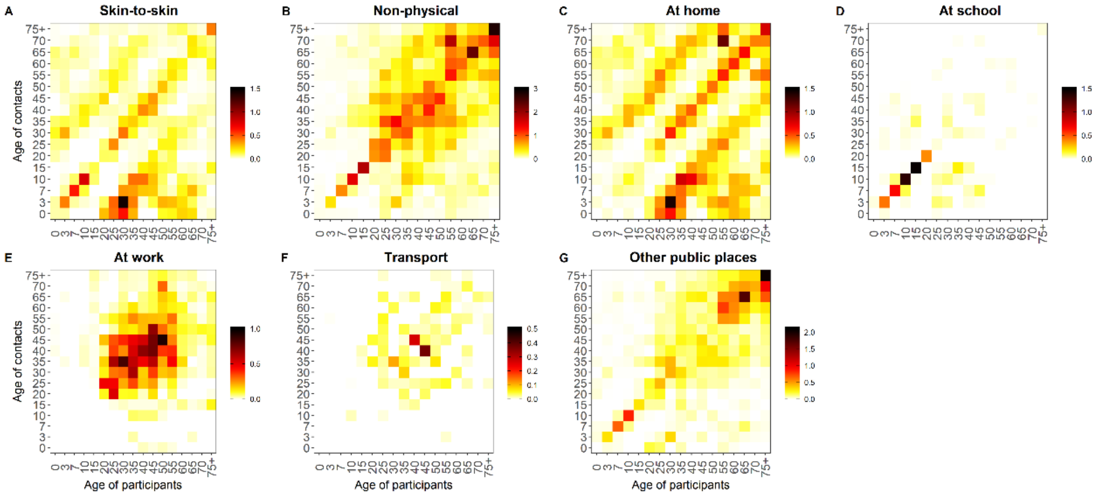

Figure S17. Human-human contact matrices consisting of the average number of contacts per day per participant in different conditions.

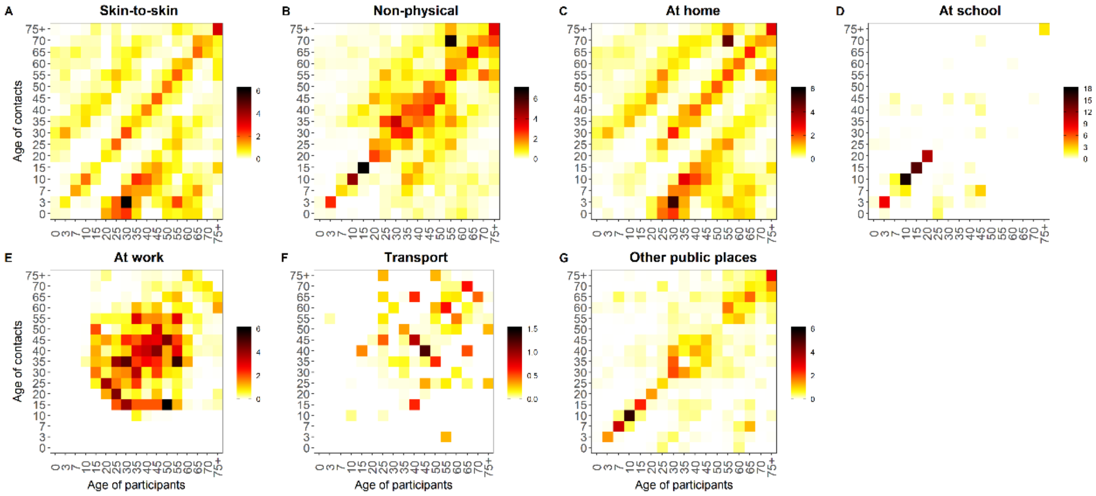

Figure S18. Human-human contact matrices consisting of the average contact duration in hours per day per participant in different conditions.

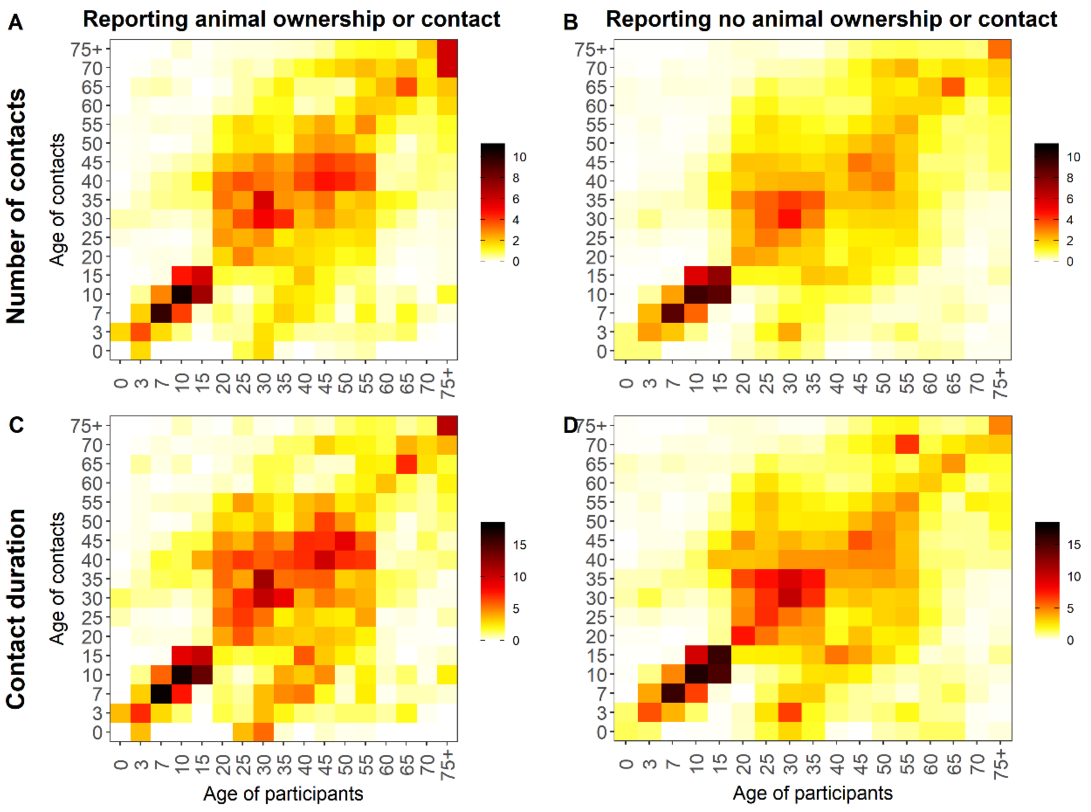

Figure S19. Weighted human-human contact matrix for participants whether or not reporting animal ownership or contact. (A and C) show the average number of contacts per day per participant; (B and D) show the average contact duration per day per participant.

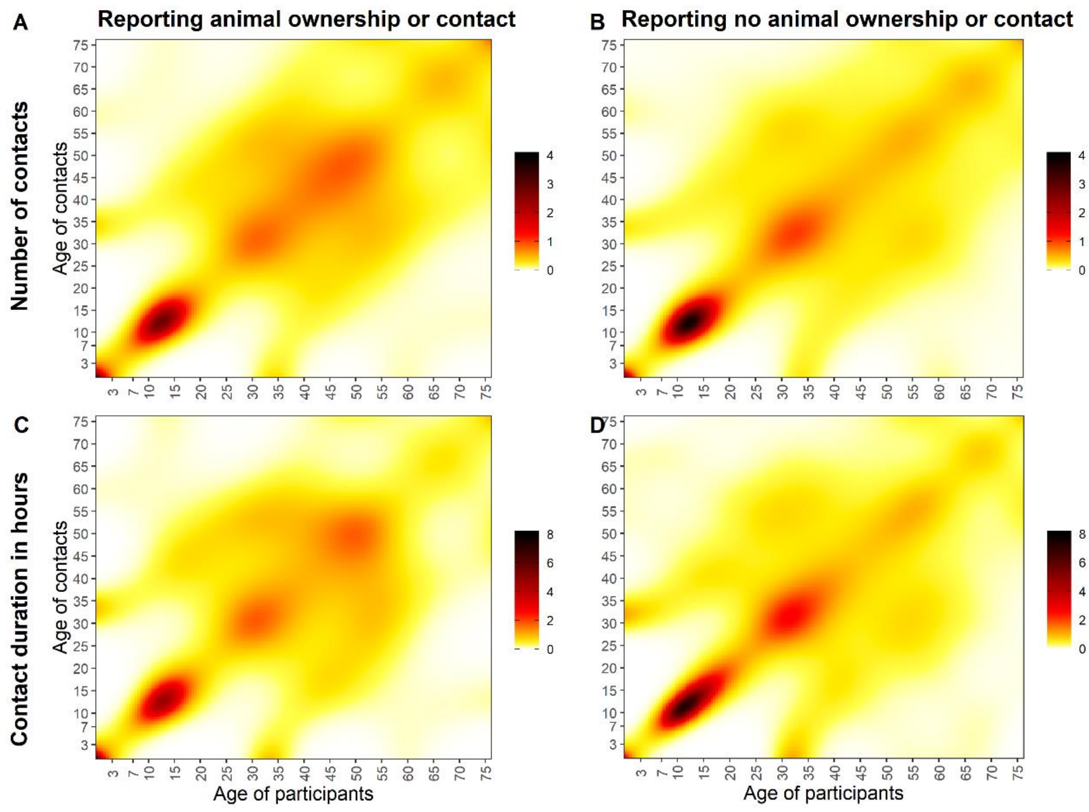

Figure S20. Smoothed human-human contact matrix for participants whether or not reporting animal ownership or contact. (A and C) show the predicted average number of contacts per day per participant; (B and D) show the predicted average contact duration per day per participant.

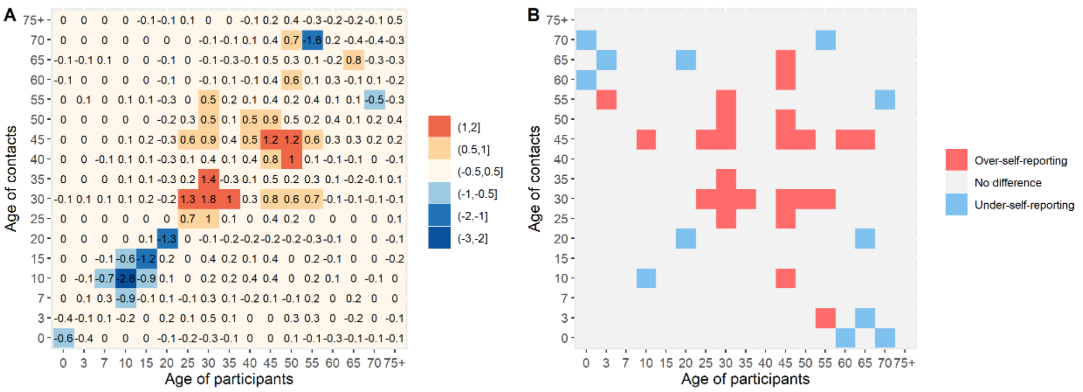

Figure S21. The bootstrapped hypothesis test for the two modes of data collection (self-reporting vs. telephone interview). (A) the bootstrapped median difference of the age-specific contact matrix derived from self-reporting and telephone interview, (B) the corresponding hypothesis test result (where participants significantly over-reported the number of age-specific contacts by telephone-interview instead of self-reporting).

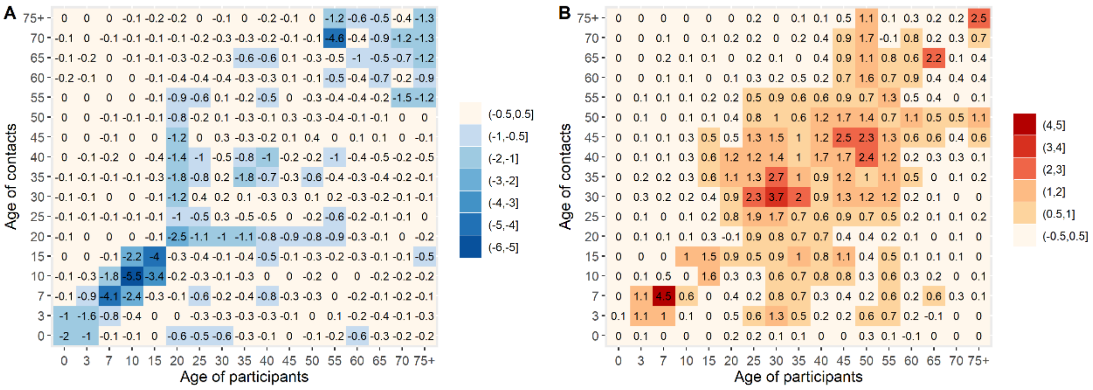

Figure S22. The difference of the observed contact matrices via self-reporting and telephone interview (A: lower 95% CI, B: upper 95%CI).

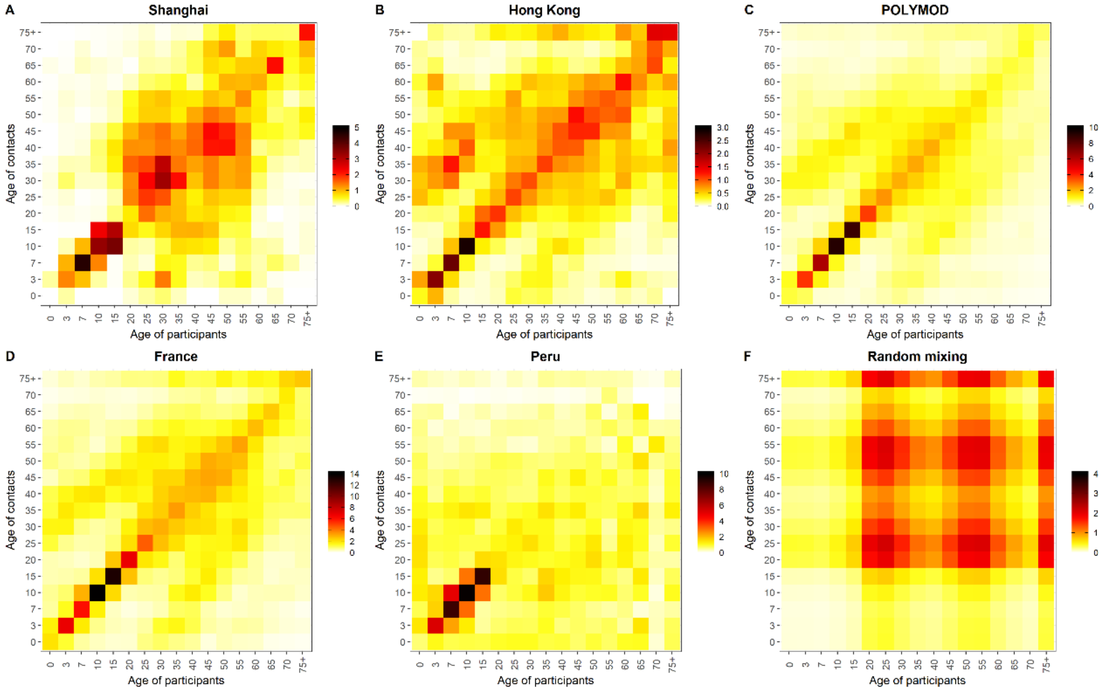

Figure S23. Contact matrixes derived from different studies to be used in epidemic models.

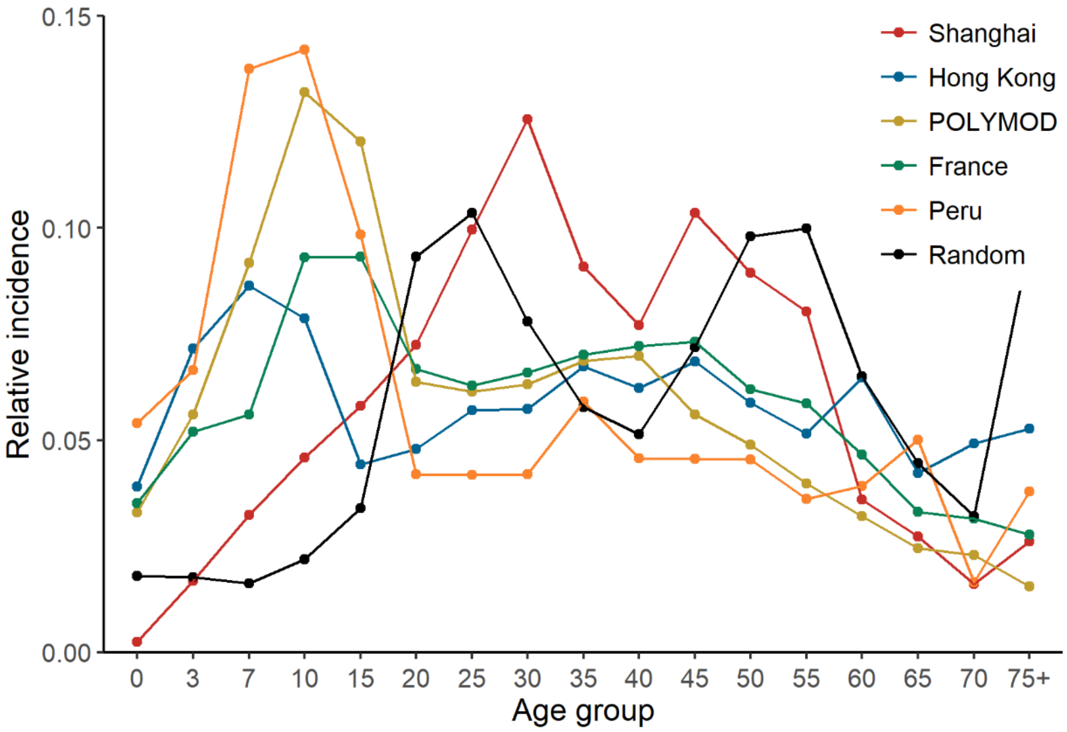

Figure S24. Relative incidence of a new emerging infection when the infection is spread between and within age groups by the contacts as observed in Fig. S24.
