# Supplementary material for: Patterns of human social contact and contact with animals in Shanghai, China
Source: Sci Rep. 2019 Oct 22;9:15141. doi: 10.1038/s41598-019-51609-8 (PMC6805924; doi:10.1038/s41598-019-51609-8)
Supplement: Supplementary file 2 — Supplementary tables [file 41598_2019_51609_MOESM2_ESM.pdf]

**Patterns of human social contact and contact with animals in Shanghai, China**

Juanjuan Zhang[1], Petra Klepac[2], Jonathan M. Read[3], Alicia Rosello[2], Xiling Wang[1], Shengjie Lai[1,4,5], Meng Li[1], Yujian Song[1], Qingzhen Wei[1], Hao Jiang[1], Juan Yang[1], Henry Lynn[1], Stefan Flasche[2], Mark Jit[2,6,7], Hongjie Yu[1*]

[1]*School of Public Health, Fudan University, Key Laboratory of Public Health Safety, Ministry of Education, Shanghai, China*

[2]*Department of Infectious Disease Epidemiology, Faculty of Epidemiology and Public Health, London School of Hygiene and Tropical Medicine, London, UK.*

[3]*Centre for Health Informatics, Computation and Statistics, Lancaster Medical School, Lancaster University, Lancashire, UK.*

[4]*WorldPop, School of Geography and Environmental Science, University of Southampton, Southampton, UK*

[5]*Flowminder Foundation, Stockholm, Sweden*

[6]*Modelling and Economics Unit, Public Health England, London, UK*

[7]*School of Public Health, University of Hong Kong, Hong Kong, China*

*Corresponding author:

Professor Hongjie Yu

School of Public Health

Fudan University

Email: yhj@fudan.edu.cn

## Supplementary Table S1-S14

**Table S1.** Comparison between all of the participants and the population of central urban districts in Shanghai.

| Characteristics | Population of central urban districts (%) (n=7,345,916) | Participants (%) (n=1000) | $P^*$ |
|---|---|---|---|
| Male sex | 3,699,171 (50.36) | 480 (49.08) | 0.424 |
| Age group | | | |
| [0,10) | 382,830 (5.21) | 121 (12.1) | <0.001 |
| [10,20) | 410,835 (5.59) | 117 (11.7) | |
| [20,30) | 1,444,886 (19.67) | 107 (10.7) | |
| [30,40) | 998,125 (13.59) | 141 (14.1) | |
| [40,50) | 905,633 (12.33) | 125 (12.5) | |
| [50,60) | 1,454,575 (19.8) | 116 (11.6) | |
| [60,70) | 805,517 (10.97) | 137 (13.7) | |
| 70+ | 943,515 (12.84) | 136 (13.6) | |

*indicates the results of chi-square test.

**Table S2.** Comparison between effective participants and all participants for contact survey.

| Characteristics | All participants (%) (n=1000) | Effective participants (%) (n=965) | $P^*$ |
|---|---|---|---|
| Male sex | 480 (49.08) | 474 (49.12) | 0.980 |
| Age group | | | |
| [0,10) | 121 (12.1) | 117 (12.12) | >0.1 |
| [10,20) | 117 (11.7) | 112 (11.61) | |
| [20,30) | 107 (10.7) | 101 (10.47) | |
| [30,40) | 141 (14.1) | 135 (13.99) | |
| [40,50) | 125 (12.5) | 122 (12.64) | |
| [50,60) | 116 (11.6) | 111 (11.5) | |
| [60,70) | 137 (13.7) | 131 (13.58) | |
| 70+ | 136 (13.6) | 136 (14.09) | |

*indicates the result of chi-square test.

**Table S3.** Median number of reported total contacts, the median number of individual contacts, the proportion of participants reporting the group contact, the median number of group contacts, the median contact duration (hour) and the median number of contact settings by different characteristics, using original data (N=965).

| Characteristics | N (%)[a] | Median of total contacts (IQR) | Median of individual contacts (IQR) | Prop. of reporting group contact (%) | Median of group contacts[b] (IQR) | Median of contact duration (IQR) | Median of contact settings (IQR) |
|---|---|---|---|---|---|---|---|
| Overall | 965 | 10 (4, 30) | 5 (3, 9) | 30.8 | 30 (25, 40) | 15.7 (7.5, 53.6) | 2 (1, 3) |
| Gender | | | | | | | |
| Male | 474 (49.1) | 10 (4.2, 31) | 5 (3, 10) | 31.4 | 30 (25, 40) | 16.8 (8, 55) | 2 (1, 2) |
| Female | 491 (50.9) | 10 (4, 29.5) | 5 (3, 9) | 30.1 | 30 (25, 40) | 14 (6.5, 52.6) | 2 (1, 3) |
| Age group | | | | | | | |
| 0-35 m | 38 (3.9) | 4 (3, 6) | 4 (3, 5.8) | 2.6 | 30 (30, 30) | 16 (12, 17.1) | 1 (1, 1) |
| 3-6 y | 50 (5.2) | 7.5 (4, 29) | 4 (4, 5.8) | 32 | 27.5 (24.8, 30.8) | 13.3 (10.3, 57.6) | 2 (1, 2) |
| 7-19 y | 141 (14.6) | 28 (6, 40) | 4 (3, 8) | 58.2 | 30 (25, 40) | 55.8 (12, 78) | 2 (1, 2) |
| 20-39 y | 236 (24.5) | 12.5 (5, 34) | 5 (3, 9) | 38.6 | 30 (25, 40) | 25 (8.1, 64.4) | 2 (2, 3) |
| 40-59 y | 233 (24.1) | 12 (5, 31) | 5 (3, 10) | 32.6 | 30 (24.8, 40) | 19.2 (7.3, 55.5) | 2 (2, 3) |
| 60-75 y | 216 (22.4) | 7 (4, 16) | 5 (3, 10) | 12.5 | 30 (26.5, 40) | 8.7 (4.6, 15.1) | 2 (1, 2) |
| >75 y | 51 (5.3) | 5 (3, 14.5) | 4 (2, 9) | 7.8 | 25.5 (20.8, 30) | 5.5 (3, 18.2) | 2 (1, 2) |
| Education | | | | | | | |
| None | 144 (14.9) | 7 (4, 28) | 4 (3, 7) | 30.6 | 26 (25, 33.5) | 16 (10.4, 51.8) | 2 (1, 2) |
| Primary school | 51 (5.3) | 13 (4.5, 41) | 5 (3, 7) | 45.1 | 31 (30, 43) | 17.2 (8, 67.6) | 2 (1.5, 3) |
| Middle school | 182 (18.9) | 10 (5, 32) | 5 (3, 9.8) | 26.9 | 35 (30, 40) | 10.6 (4.9, 52) | 2 (2, 2) |
| High school | 285 (29.5) | 10 (4, 29) | 5 (3, 10) | 26.7 | 30 (25, 40) | 12 (6.5, 48) | 2 (2, 3) |
| College and above | 294 (30.5) | 10.5 (5, 31) | 5 (3, 10) | 35.4 | 30 (22, 40) | 23.9 (8.2, 57.4) | 2 (2, 3) |
| Unknown | 9 (0.9) | 3 (2, 4) | 3 (2, 3) | 11.1 | 30 (30, 30) | 5.6 (1.4, 9) | 1 (1, 2) |
| Work type | | | | | | | |
| Pre-school | 79 (8.2) | 5 (4, 11) | 4 (3, 6) | 13.9 | 30 (30, 34.5) | 16 (12, 20.9) | 1 (1, 2) |
| Students | 173 (17.9) | 28 (6, 39) | 4 (3, 7) | 56.6 | 30 (25, 40) | 50.9 (12, 77.2) | 2 (2, 3) |
| Service workers | 192 (19.9) | 22.5 (6, 40) | 5 (3, 10) | 49 | 30 (25, 40) | 37.8 (8.2, 71.6) | 2 (2, 3) |
| Professionals | 171 (17.7) | 11 (6, 25.5) | 6 (3, 10) | 30.4 | 30 (20.8, 41.2) | 27.7 (10.3, 52.3) | 2 (2, 3) |
| Unemployed | 29 (3) | 6 (3, 13) | 4 (3, 10) | 3.4 | 100 (100, 100) | 7.1 (4.6, 11.1) | 2 (1, 2) |
| Retired | 278 (28.8) | 6 (3, 15) | 5 (3, 10) | 9.7 | 30 (25, 32.5) | 8 (4, 15.1) | 2 (1, 2) |
| Others[c] | 37 (3.8) | 11 (5, 33) | 6 (3, 8) | 35.1 | 35 (30, 50) | 16 (8, 56.1) | 2 (2, 3) |
| Unknown | 6 (0.6) | 3 (3, 9) | 3 (3, 6) | 16.7 | 50 (50, 50) | 9.2 (2.7, 30.6) | 1.5 (1, 2.8) |
| Individual annual income ($) | | | | | | | |
| 1-8,000 | 223 (23.1) | 9 (4, 20) | 5 (3, 10) | 17 | 30 (22.8, 40) | 9.6 (4, 22.3) | 2 (1, 3) |
| 8,001-11,000 | 216 (22.4) | 10 (4.8, 34) | 5 (3, 10) | 33.8 | 30 (25, 40) | 17 (8, 56.6) | 2 (2, 3) |
| 11,001-15,000 | 89 (9.2) | 10 (5, 32) | 6 (4, 10) | 32.6 | 32 (25, 40) | 20.3 (8.1, 59.5) | 2 (2, 3) |
| >15,000 | 90 (9.3) | 14 (7, 27) | 6 (2, 10) | 36.7 | 30 (22, 50) | 38.6 (12.6, 58.2) | 2 (2, 3) |
| No income | 74 (7.7) | 7 (4, 19.5) | 5 (3, 8) | 20.3 | 28 (20, 37.5) | 8.2 (5.2, 27.8) | 2 (1, 2) |
| Not available[d] | 236 (24.5) | 15 (4, 34) | 4 (3, 6) | 43.6 | 30 (25, 40) | 24.2 (12, 69.7) | 2 (1, 2) |
| Unknown | 37 (3.8) | 5 (4, 17) | 5 (2, 8) | 16.2 | 25 (21.2, 51.2) | 8 (5.1, 19.2) | 2 (1, 2) |
| Years of living in Shanghai | | | | | | | |
| 0-10 years | 90 (9.3) | 10.5 (4, 35.5) | 4.5 (2, 10) | 35.6 | 35 (26.5, 40) | 16 (8, 61.9) | 2 (2, 2) |
| >10 years | 748 (77.5) | 10 (5, 30) | 5 (3, 10) | 30.6 | 30 (25, 40) | 15.1 (6.9, 53.1) | 2 (2, 3) |
| Not available[e] | 123 (12.7) | 7 (4, 26) | 4 (3, 7) | 28.5 | 30 (25, 35) | 16 (12, 50.6) | 2 (1, 2) |
| Unknown | 4 (0.4) | 9.5 (3.8, 19.5) | 4.5 (3.8, 5.8) | 25 | 25 (25, 25) | 4.5 (1.5, 21.9) | 2 (1.8, 2.2) |

| | | | | | | | |
|---|---|---|---|---|---|---|---|
| Household size | | | | | | | |
| 1-2 | 279 (28.9) | 7 (3.5, 19) | 5 (3, 10) | 17.2 | 30 (27.2, 40) | 8.5 (4, 25.3) | 2 (1, 2) |
| 3-4 | 544 (56.4) | 12 (5, 33) | 5 (3, 9) | 36.9 | 30 (25, 40) | 20 (8.2, 59.4) | 2 (2, 3) |
| >4 | 142 (14.7) | 11 (5, 31) | 5 (4, 10) | 33.8 | 30 (25, 40) | 17.8 (11.2, 64.9) | 2 (1, 2) |
| Mode of data collection | | | | | | | |
| Self-reporting | 386 (40) | 10 (4, 30) | 5 (3, 10) | 27.2 | 30 (25, 38) | 13.5 (7.2, 52.5) | 2 (1, 3) |
| Telephone interview | 579 (60) | 10 (5, 30.5) | 5 (3, 8) | 33.2 | 30 (25, 40) | 16.5 (8, 54.2) | 2 (2, 3) |
| Population density | | | | | | | |
| Low | 294 (30.5) | 8 (4, 25.8) | 4 (3, 6) | 29.6 | 30 (22, 40) | 12.5 (7, 47.7) | 2 (1, 2) |
| Moderate | 384 (39.8) | 10 (5, 29) | 5 (3, 10) | 31 | 30 (25, 40) | 16.9 (8, 52) | 2 (2, 3) |
| High | 287 (29.7) | 12 (5, 33.5) | 5 (3, 10.5) | 31.7 | 30 (25, 40) | 16 (7, 58.2) | 2 (1, 3) |
| Day type | | | | | | | |
| Weekday | 705 (73.1) | 12 (5, 32) | 5 (3, 10) | 34.8 | 30 (25, 40) | 17.2 (8, 57.6) | 2 (2, 3) |
| Weekend | 260 (26.9) | 7 (4, 19.2) | 5 (3, 8) | 20 | 30 (26.5, 40) | 11.4 (6.1, 27.2) | 2 (1, 2) |
| Typical day | | | | | | | |
| Yes | 812 (84.1) | 10 (4, 30) | 5 (3, 9) | 31.2 | 30 (25, 40) | 14.9 (7.5, 54.8) | 2 (1, 2.2) |
| No | 126 (13.1) | 14 (6, 31) | 7 (4, 12) | 31 | 30 (22.5, 40) | 23.5 (8.7, 52.5) | 2 (2, 3) |
| Unknown | 27 (2.8) | 6 (4, 26) | 5 (3.5, 8.5) | 18.5 | 25 (25, 30) | 9 (3.5, 37.3) | 2 (1, 3) |
| Travel outside the subdistrict | | | | | | | |
| Almost daily | 597 (61.9) | 12 (5, 32) | 5 (3, 9) | 36.7 | 30 (25, 40) | 22 (8.1, 59.3) | 2 (2, 3) |
| Not daily | 341 (35.3) | 8 (4, 20) | 5 (3, 10) | 20.8 | 30 (25, 40) | 11.5 (5.3, 30) | 2 (1, 2) |
| Unknown | 27 (2.8) | 7 (4, 19) | 5 (3, 6) | 25.9 | 25 (20.5, 32.5) | 5.2 (1.6, 27) | 2 (1, 2) |
| Animal ownership | | | | | | | |
| Owner | 164 (17) | 16 (5, 34.5) | 5 (3, 10) | 38.4 | 30 (25, 40) | 17.4 (8, 61) | 2 (2, 3) |
| Not owner | 801 (83) | 10 (4, 29) | 5 (3, 9) | 29.2 | 30 (25, 40) | 15.1 (7.3, 51.3) | 2 (1, 3) |
| Animal contact | | | | | | | |
| Yes | 171 (17.7) | 16 (5, 33.5) | 5 (3, 10) | 35.7 | 30 (25, 40) | 17.1 (9, 57.8) | 2 (2, 3) |
| No | 794 (82.3) | 10 (4, 29) | 5 (3, 9) | 29.7 | 30 (25, 40) | 15.2 (7.1, 52.2) | 2 (1, 3) |

[a]Data presented as no. (%) of respondents; percentages may not total 100 because of rounding.

[b]Median of group contacts for participants reporting a group contact.

[c]Most are freelances.

[d]Students or pre-school children who don’t have individual income.

[e]Indicates children younger than 10 years old, as their years of living in Shanghai cannot exceed 10 years.

**Table S4.** Number of participants who reported owning at least one animal or having contact with at least one animal on the assigned day.

| **Animals** | **Number of participants owning an animal (n=965)** | **Number of participants who had contact with an animal (n=965)** |
|---|---|---|
| **Overall** | 164 (16.99%) | 171 (17.72%) |
| **Pets** | 148 (15.34%) | 159 (16.48%) |
| Dog | 80 (8.29%) | 97 (10.05%) |
| Cat | 50 (5.18%) | 63 (6.53%) |
| Fish | 23 (2.38%) | 23 (2.38%) |
| Bird | 6 (0.62%) | 4 (0.41%) |
| Rabbit | 3 (0.31%) | 3 (0.31%) |
| Rat | 3 (0.31%) | 2 (0.21%) |
| **Poultry** | 0 (0.00%) | 2 (0.21%) |
| Chicken | 0 (0.00%) | 2 (0.21%) |
| Pigeon | 0 (0.00%) | 2 (0.21%) |
| **Livestock** | 1 (0.1%) | 0 (0.00%) |
| Sheep | 1 (0.1%) | 0 (0.00%) |
| **Others**[#] | 22 (2.28%) | 17 (1.76%) |

[#]Most are pets, such as hedgehog, turtle, lizard and squirrel.

**Table S5.** Proportion of participants owning or having contact with at least one animal, median number of animal contacts, and contact duration by different characteristics (N=965).

| Characteristics | N (%)[a] | Proportion of animal ownership (%) | Proportion of animal contact (%) | Median of animal contacts (IQR) | Median of contact duration (IQR) |
|---|---|---|---|---|---|
| Overall | 965 | 16.99 | 17.72 | 1 (1, 2) | 0.62 (0.16, 4) |
| Gender | | | | | |
| Male | 474 (49.1) | 16.46 | 16.46 | 1 (1, 2) | 0.62 (0.13, 2.66) |
| Female | 491 (50.9) | 17.52 | 18.94 | 1 (1, 2) | 2.5 (0.16, 4) |
| Age group | | | | | |
| 0-35 m | 38 (3.9) | 2.63 | 5.26 | 1 (1, 1) | 0.33 (0.19, 0.47) |
| 3-6 y | 50 (5.2) | 14 | 20 | 1 (1, 5) | 0.55 (0.07, 4) |
| 7-19 y | 141 (14.6) | 18.44 | 18.44 | 2 (1, 2) | 0.2 (0.08, 1.23) |
| 20-39 y | 236 (24.5) | 16.95 | 16.53 | 1 (1, 2) | 2.5 (0.16, 4) |
| 40-59 y | 233 (24.1) | 21.03 | 21.03 | 1 (1, 2) | 2.5 (0.32, 4) |
| 60-75 y | 216 (22.4) | 16.2 | 16.67 | 1 (1, 2) | 0.62 (0.16, 4) |
| >75 y | 51 (5.3) | 11.76 | 17.65 | 1 (1, 1) | 1.33 (0.1, 2.99) |
| Education | | | | | |
| None | 144 (14.9) | 10.42 | 13.89 | 1.5 (1, 3.5) | 0.16 (0.04, 0.78) |
| Primary school | 51 (5.3) | 19.61* | 21.57 | 2 (1, 2) | 0.18 (0.1, 0.62) |
| Middle school | 182 (18.9) | 18.68 | 17.03 | 1 (1, 1.5) | 1.23 (0.39, 3.33) |
| High school | 285 (29.5) | 18.6* | 20.35* | 1 (1, 2) | 2.5 (0.16, 4) |
| College and above | 294 (30.5) | 16.67* | 16.33 | 1 (1, 2) | 1.64 (0.16, 4) |
| Unknown | 9 (0.9) | 33.33 | 33.33 | 1 (1, 1) | 1.56 (1.09, 2.03) |
| Work type | | | | | |
| Pre-school | 79 (8.2) | 8.86 | 13.92 | 1 (1, 1) | 0.16 (0.04, 2.47) |
| Students | 173 (17.9) | 18.5 | 19.08 | 2 (1, 2) | 0.28 (0.14, 2.5) |
| Service workers | 192 (19.9) | 18.75 | 17.19 | 1 (1, 2.5) | 0.62 (0.16, 2.54) |
| Professionals | 171 (17.7) | 19.88* | 19.88 | 1 (1, 2) | 2.5 (0.62, 4) |
| Unemployed | 29 (3) | 13.79 | 13.79 | 2 (1, 3.5) | 0.62 (0.48, 0.77) |
| Retired | 278 (28.8) | 16.19 | 16.91 | 1 (1, 2) | 2.5 (0.16, 4) |
| Others[b] | 37 (3.8) | 16.22 | 24.32 | 1 (1, 2.75) | 0.62 (0.16, 2.5) |
| Unknown | 6 (0.6) | 0 | 0 | - | - |
| Individual annual income ($) | | | | | |
| 1-8,000 | 223 (23.1) | 21.08 | 23.32 | 1 (1, 2) | 0.62 (0.16, 2.58) |
| 8,001-11,000 | 216 (22.4) | 17.59 | 17.13* | 1 (1, 2) | 2.5 (0.62, 4) |
| 11,001-15,000 | 89 (9.2) | 13.48* | 13.48* | 1.5 (1, 3) | 2.5 (0.12, 4) |
| >15,000 | 90 (9.3) | 23.33 | 21.11 | 1 (1, 1) | 4 (0.62, 4) |
| No income | 74 (7.7) | 10.81* | 9.46* | 1 (1, 1.25) | 0.62 (0.08, 2.5) |
| Not available[c] | 236 (24.5) | 14.83* | 16.95* | 1 (1, 2) | 0.24 (0.08, 2.5) |
| Unknown | 37 (3.8) | 8.11* | 10.81* | 1 (1, 3) | 0.12 (0.07, 0.27) |
| Years of living in Shanghai | | | | | |
| 0-10 years | 90 (9.3) | 5.56 | 10 | 2 (1, 4.25) | 0.18 (0.14, 0.9) |
| >10 years | 748 (77.5) | 19.39* | 19.52* | 1 (1, 2) | 0.7 (0.16, 4) |

| | | | | | |
|---|---|---|---|---|---|
| Not available[d] | 123 (12.7) | 10.57 | 13.01 | 1 (1, 5) | 0.16 (0.04, 2.47) |
| Unknown | 4 (0.4) | 25 | 0 | - | - |
| Household size | | | | | |
| 1-2 | 279 (28.9) | 16.13 | 17.92 | 1 (1, 2) | 2.5 (0.24, 4) |
| 3-4 | 544 (56.4) | 18.2 | 18.57 | 1 (1, 2) | 0.62 (0.16, 4) |
| >4 | 142 (14.7) | 14.08 | 14.08 | 2 (1, 2) | 0.62 (0.14, 4) |
| Mode of data collection | | | | | |
| Self-reporting | 386 (40) | 18.39 | 20.47 | 1 (1, 3) | 0.78 (0.16, 4) |
| Telephone interview | 579 (60) | 16.06 | 15.89 | 1 (1, 2) | 0.62 (0.16, 4) |
| Population density | | | | | |
| Low | 294 (30.5) | 15.65 | 15.99 | 1 (1, 1) | 2.5 (0.16, 4) |
| Moderate | 384 (39.8) | 15.36 | 16.15 | 1 (1, 2) | 0.62 (0.16, 4) |
| High | 287 (29.7) | 20.56 | 21.6 | 2 (1, 2.25) | 0.7 (0.16, 4) |
| Day type | | | | | |
| Weekday | 705 (73.1) | - | 17.16 | 1 (1, 2) | 0.62 (0.16, 4) |
| Weekend | 260 (26.9) | - | 19.23 | 1 (1, 2) | 0.62 (0.16, 2.58) |
| Typical day | | | | | |
| Yes | 812 (84.1) | - | 17.73 | 1 (1, 2) | 0.62 (0.16, 4) |
| No | 126 (13.1) | - | 19.84 | 1 (1, 2) | 0.62 (0.16, 4) |
| Unknown | 27 (2.8) | - | 7.41 | 1 (1, 1) | 0.62 (0.62, 0.62) |
| Travel outside the subdistrict | | | | | |
| Almost daily | 597 (61.9) | 17.92 | 17.25 | 1 (1, 2) | 0.62 (0.16, 4) |
| Not daily | 341 (35.3) | 15.25 | 18.48 | 1 (1, 4) | 0.62 (0.14, 4) |
| Unknown | 27 (2.8) | 18.52 | 18.52 | 1 (1, 2) | 4 (0.04, 4) |

[a]Data presented as no. (%) of respondents; percentages may not total 100 because of rounding.

[b]Most are freelances.

[c]Students or pre-school children who don't have individual income.

[d]Indicates children younger than 10 years old, as their years of living in Shanghai cannot exceed 10 years.

[*]Indicates statistically significant difference from the first level of each factor in the univariate regression model (at the 5% level).

**Table S6.** Median number of reported total contacts, the median number of individual contacts, the proportion of participants reporting the group contact, the median number of group contacts, the median contact duration (hour) and the median number of contact settings by different characteristics, for participants owning or touching animals (N=194).

| Characteristics | N (%)[a] | Median of total contacts (IQR) | Median of individual contacts (IQR) | Prop. of reporting group contact (%) | Median of group contacts[b] (IQR) | Median of contact duration (IQR) | Median of contact settings (IQR) |
|---|---|---|---|---|---|---|---|
| Overall | 194 | 16.5 (6, 33.75) | 5 (4, 10) | 36.1 | 30 (25, 40) | 17.4 (8, 58.4) | 2 (2, 3) |
| Gender | | | | | | | |
| Male | 94 (48.5) | 17 (6, 32.8) | 5 (3, 9.8) | 39.4 | 30 (22, 35) | 22.3 (9.3, 56.2) | 2 (2, 2) |
| Female | 100 (51.5) | 15.5 (5, 34.5) | 5.5 (4, 10) | 33 | 30 (28, 40) | 15 (6.6, 60.1) | 2 (2, 3) |
| Age group | | | | | | | |
| 0-35 m | 2 (1) | 3.5 (3.2, 3.8) | 3.5 (3.2, 3.8) | 0 | NA | 10.6 (9.9, 11.3) | 1 (1, 1) |
| 3-6 y | 10 (5.2) | 14.5 (5.8, 31.2) | 5 (4, 5) | 40 | 30 (28.8, 30) | 14.2 (9.7, 62.6) | 2 (2, 2) |
| 7-19 y | 31 (16) | 30 (20, 40.5) | 4 (3, 11) | 64.5 | 30 (25, 40) | 58.5 (22.4, 76.1) | 2 (1, 2) |
| 20-39 y | 47 (24.2) | 12 (5.5, 38.5) | 6 (3.5, 9) | 36.2 | 40 (30, 45) | 29.2 (8, 71.4) | 2 (2, 3) |
| 40-59 y | 55 (28.4) | 17 (7.5, 34) | 6 (4, 11.5) | 40 | 30 (24.2, 38.8) | 26 (8.6, 58.5) | 2 (2, 3) |
| 60-75 y | 40 (20.6) | 9 (4.8, 23) | 6 (4, 11) | 15 | 27.5 (22.8, 30) | 10.3 (5.4, 15.8) | 2 (2, 2) |
| >75 y | 9 (4.6) | 7 (5, 33) | 6 (4, 17) | 11.1 | 30 (30, 30) | 11.8 (2.9, 24) | 2 (1, 2) |
| Education | | | | | | | |
| None | 22 (11.3) | 25.5 (5.8, 31.2) | 4.5 (3.2, 7.5) | 45.5 | 27.5 (25, 30) | 43.9 (12.1, 62.6) | 2 (1.2, 2) |
| Primary school | 12 (6.2) | 41 (21.8, 49) | 6.5 (5, 8.2) | 75 | 30 (28, 43) | 55.6 (16.5, 81.1) | 2.5 (2, 3) |
| Middle school | 37 (19.1) | 10 (5, 30) | 5 (4, 8) | 24.3 | 30 (30, 40) | 10.7 (5.5, 25.2) | 2 (2, 2) |
| High school | 62 (32) | 14 (5, 29) | 6.5 (4, 14) | 25.8 | 30 (21.5, 31.2) | 12.1 (7.7, 44) | 2 (2, 3) |
| College and above | 58 (29.9) | 19.5 (6, 39.2) | 5 (2.2, 9.8) | 43.1 | 35 (25, 45) | 36.6 (12.1, 72.3) | 2 (2, 2) |
| Unknown | 3 (1.5) | 4 (3.5, 18.5) | 3 (3, 3.5) | 33.3 | 30 (30, 30) | 9 (7.3, 30.5) | 2 (1.5, 2) |
| Work type | | | | | | | |
| Pre-school | 11 (5.7) | 9 (4.5, 30.5) | 4 (3.5, 5) | 36.4 | 30 (28.8, 30) | 12.5 (10.6, 62.1) | 2 (1, 2) |
| Students | 38 (19.6) | 28.5 (10.5, 40.8) | 5 (3, 10.2) | 57.9 | 30 (25, 40) | 57.8 (15.5, 77.7) | 2 (2, 2) |
| Service workers | 39 (20.1) | 21 (5, 36.5) | 5 (4, 14.5) | 46.2 | 30 (22.8, 38.8) | 41.5 (8.5, 65.7) | 2 (2, 3) |
| Professionals | 38 (19.6) | 14.5 (9, 28.5) | 6.5 (4, 10) | 39.5 | 35 (24.5, 47.5) | 32.5 (12.3, 62.4) | 2 (2, 3) |
| Unemployed | 6 (3.1) | 15 (6.5, 35.5) | 9 (6.5, 19) | 0 | NA | 6 (4.2, 8.5) | 2 (2, 2) |
| Retired | 53 (27.3) | 7 (4, 20) | 6 (4, 10) | 13.2 | 30 (22.5, 30) | 10.6 (5.1, 17.1) | 2 (2, 2) |
| Others[c] | 9 (4.6) | 11 (7, 37) | 5 (3, 7) | 44.4 | 35.5 (28.8, 55.8) | 20.5 (8, 56.1) | 2 (2, 3) |
| Unknown | 55 (28.4) | 11 (6, 27.5) | 7 (4, 10) | 21.8 | 32.5 (23.8, 50) | 11.4 (5.3, 29.3) | 2 (2, 3) |
| Individual annual income ($) | | | | | | | |
| 1-8,000 | 55 (28.4) | 11 (6, 27.5) | 7 (4, 10) | 21.8 | 32.5 (23.8, 50) | 11.4 (5.3, 29.3) | 2 (2, 3) |
| 8,001-11,000 | 42 (21.6) | 15.5 (5, 33.8) | 5 (4, 10) | 35.7 | 30 (25, 34) | 17.4 (9, 55.9) | 2 (2, 3) |
| 11,001-15,000 | 15 (7.7) | 15 (10, 37) | 9 (5, 14) | 33.3 | 30 (25, 40) | 36 (8.9, 57.2) | 2 (2, 3) |
| >15,000 | 23 (11.9) | 14 (5, 31.5) | 4 (2, 8.5) | 39.1 | 35 (25, 45) | 33 (10, 66.8) | 2 (2, 2) |
| No income | 9 (4.6) | 8 (6, 52) | 8 (5, 10) | 33.3 | 28 (24, 64) | 9 (7.1, 52.7) | 2 (2, 3) |
| Not available[d] | 45 (23.2) | 28 (8, 36) | 5 (3, 7) | 53.3 | 30 (25, 40) | 55.8 (12.5, 72.7) | 2 (1, 2) |

| | | | | | | | |
|---|---|---|---|---|---|---|---|
| Unknown | 5 (2.6) | 26 (5, 26) | 5 (4, 6) | 40 | 22.5 (21.2, 23.8) | 5.6 (5.1, 44.2) | 2 (2, 2) |
| Years of living in Shanghai | | | | | | | |
| 0-10 years | 9 (4.6) | 11 (5, 41) | 5 (5, 7) | 33.3 | 40 (34, 40.5) | 16.6 (7.9, 67.5) | 2 (2, 2) |
| >10 years | 166 (85.6) | 17 (5.2, 34) | 6 (4, 10) | 35.5 | 30 (25, 40) | 17.9 (8, 57.1) | 2 (2, 3) |
| Not available[e] | 18 (9.3) | 21.5 (5, 32) | 4 (3, 5) | 44.4 | 30 (25, 30) | 21.2 (9.9, 62.6) | 2 (1, 2) |
| Unknown | 1 (0.5) | 15 (15, 15) | 5 (5, 5) | 0 | NA | 7.4 (7.4, 7.4) | 2 (2, 2) |
| Household size | | | | | | | |
| 1-2 | 55 (28.4) | 8 (4.5, 27.5) | 5 (3, 9) | 23.6 | 30 (30, 35) | 11.4 (4.9, 43.8) | 2 (2, 2) |
| 3-4 | 115 (59.3) | 19 (8, 33) | 5 (3, 10) | 41.7 | 30 (21.8, 40) | 29.2 (10, 61.2) | 2 (2, 3) |
| >4 | 24 (12.4) | 13.5 (4.8, 40.5) | 5 (4, 10) | 37.5 | 40 (30, 40) | 15.4 (5.5, 77.5) | 2 (1, 2) |
| Mode of data collection | | | | | | | |
| Self-reporting | 87 (44.8) | 19 (5.5, 32.5) | 6 (3, 13.5) | 34.5 | 29 (24.2, 33.8) | 16 (7.8, 58.9) | 2 (2, 3) |
| Telephone interview | 107 (55.2) | 14 (5, 34) | 5 (4, 8) | 37.4 | 30 (25, 40) | 18.5 (8.4, 57.4) | 2 (2, 2) |
| Population density | | | | | | | |
| Low | 55 (28.4) | 9 (5, 26.5) | 5 (3.5, 7) | 29.1 | 30 (23.8, 33.5) | 13.1 (7.8, 49.6) | 2 (2, 2) |
| Moderate | 69 (35.6) | 14 (7, 34) | 6 (3, 8) | 37.7 | 30 (25, 40) | 17.1 (9, 56) | 2 (2, 3) |
| High | 70 (36.1) | 22.5 (8, 40.8) | 6 (4, 15) | 40 | 30 (25, 40) | 32.8 (9, 66.8) | 2 (2, 3) |
| Day type | | | | | | | |
| Weekday | 139 (71.6) | 19 (6.5, 34) | 5 (3, 10) | 40.3 | 30 (25, 40) | 28.3 (9.4, 60.4) | 2 (2, 3) |
| Weekend | 55 (28.4) | 10 (4.5, 30.5) | 6 (4, 9) | 25.5 | 30 (30, 40) | 10.5 (5.5, 48.6) | 2 (1, 2) |
| Typical day | | | | | | | |
| Yes | 159 (82) | 15 (5, 33.5) | 5 (3, 9.5) | 37.1 | 30 (25, 40) | 17 (8, 59.7) | 2 (2, 3) |
| No | 31 (16) | 20 (9, 35) | 6 (4.5, 15) | 32.3 | 31.5 (25, 38.8) | 18.1 (10.3, 56.9) | 2 (2, 2.5) |
| Unknown | 4 (2.1) | 18 (7.5, 29.2) | 7 (5.2, 11.8) | 25 | 30 (30, 30) | 16.7 (6.6, 32.8) | 2 (2, 2.2) |
| Travel outside the subdistrict | | | | | | | |
| Almost daily | 118 (60.8) | 17.5 (6, 34) | 6 (4, 10.8) | 37.3 | 31.5 (25, 40) | 25.9 (9.3, 59.7) | 2 (2, 3) |
| Not daily | 69 (35.6) | 15 (5, 32) | 5 (3, 10) | 36.2 | 30 (22, 30) | 13.3 (7.4, 57.5) | 2 (2, 3) |
| Unknown | 7 (3.6) | 9 (5, 11.5) | 5 (5, 6.5) | 14.3 | 30 (30, 30) | 5 (1.3, 9.1) | 2 (1.5, 2) |

[a]Data presented as no. (%) of respondents; percentages may not total 100 because of rounding.

[b]Median of group contacts for participants reporting a group contact.

[c]Most are freelances.

[d]Students or pre-school children who don’t have individual income.

[e]Indicates children younger than 10 years old, as their years of living in Shanghai cannot exceed 10 years.

**Table S7.** Absolute standardized difference before and after propensity score matching.

| Items | Before matching | After matching |
|---|---|---|
| Gender | 16.11* | 4.16 |
| Age group | 11.72 | 8.10 |
| Work type | 6.11 | 2.90 |
| Education level | 28.94* | 6.71 |
| Individual annual income | 10.12 | 4.08 |
| Years of living in Shanghai | 4.82 | 1.54 |
| Household size | 3.48 | 1.19 |
| Day type | 5.85 | 2.39 |
| Population density level | 58.35* | 20.60 |
| Typical day | 6.48 | 10.22 |
| Travel outside of subdistrict | 24.81* | 12.00 |
| Interviewer# | 4.78 | 0.86 |

*indicates statistically significant difference.

#includes four interviewers, two males and two females, who provided help for participants who chose self-reporting or completed the diary by telephone interview on the behalf of the participants.

**Table S8.** Median number of reported total contacts, the median number of individual contacts, the proportion of participants reporting the group contact, the median number of group contacts, the median contact duration (in hours) and the median number of contact settings by different characteristics, using propensity score matched data (N=772).

| Characteristics | N (%)[a] | Median of total contacts (IQR) | Median of individual contacts (IQR) | Prop. of reporting group contact (%) | Median of group contacts[b] (IQR) | Median of contact duration (IQR) | Median of contact settings (IQR) |
|---|---|---|---|---|---|---|---|
| Overall | 772 | 10 (4, 30.2) | 5 (3, 7.4) | 30.1 | 30 (25, 40) | 15.2 (7.5, 55.1) | 2 (1, 3) |
| Gender | | | | | | | |
| Male | 350 (45.3) | 10 (5, 31) | 5 (3, 9) | 30.3 | 30 (25, 40) | 16.4 (8, 55.3) | 2 (1, 2) |
| Female | 422 (54.7) | 10 (4, 30) | 5 (3, 10) | 29.9 | 30 (25, 40) | 13.7 (6.5, 53.1) | 2 (1, 3) |
| Age group | | | | | | | |
| 0-35 m | 36 (4.7) | 4 (3, 6.2) | 4 (3, 5.2) | 2.8 | 30 (30, 30) | 15.5 (11.6, 17.8) | 1 (1, 1) |
| 3-6 y | 48 (6.2) | 7.5 (4, 29)* | 4 (3.8, 6) | 31.2 | 30 (25, 31.5) | 13.2 (10.1, 56.3) | 2 (1, 2) |
| 7-19 y | 131 (17) | 29 (7.5, 40)* | 4 (3, 8) | 58.8* | 31 (25, 40) | 57.5 (12.2, 79.2)* | 2 (1, 2) |
| 20-39 y | 160 (20.7) | 13 (5, 35)* | 5 (3, 9) | 37.5* | 35 (26.5, 40.2) | 23.4 (8.1, 64.7)* | 2 (2, 3)* |
| 40-59 y | 175 (22.7) | 12 (5, 28)* | 6 (3, 10)* | 30.9 | 30 (22.5, 40) | 16.1 (7, 54.2)* | 2 (2, 3)* |
| 60-75 y | 177 (22.9) | 7 (4, 16)* | 5 (3, 10) | 12.4 | 30 (28.5, 40) | 8.6 (5, 15.6) | 2 (1, 3) |
| >75 y | 45 (5.8) | 5 (3, 17)* | 4 (2, 10)* | 6.7 | 21 (20.5, 25.5) | 5.5 (3.1, 18.6) | 2 (1, 2) |
| Education | | | | | | | |
| None | 134 (17.4) | 7.5 (4, 28) | 4 (3, 7) | 29.9 | 27.5 (25, 35) | 16 (9.8, 51.2) | 2 (1, 2) |
| Primary school | 49 (6.3) | 10 (4, 41) | 5 (3, 7) | 42.9 | 35 (30, 43) | 15.7 (8, 67.6) | 2 (1, 3) |
| Middle school | 168 (21.8) | 10 (5, 33) | 5 (3, 9.2) | 28.6 | 35 (30, 40) | 11.1 (5.1, 55.8) | 2 (2, 2) |
| High school | 231 (29.9) | 10 (4, 29.5)* | 5 (3, 10)* | 25.5 | 30 (25, 40) | 12.1 (6.5, 48.3) | 2 (2, 3)* |
| College and above | 184 (23.8) | 11 (5, 29.2)* | 5 (3, 10) | 34.8 | 30 (20, 40) | 21.4 (8.3, 56) | 2 (2, 3) |
| Unknown | 6 (0.8) | 2.5 (1.2, 3)* | 2.5 (1.2, 3)* | 0 | - | 6.8 (4.4, 8.8)* | 1 (1, 1) |
| Work type | | | | | | | |
| Pre-school | 76 (9.8) | 5 (3.8, 12.2) | 4 (3, 6) | 14.5 | 30 (30, 34.5) | 14.2 (12, 21.8) | 1 (1, 2) |
| Students | 152 (19.7) | 29 (6.8, 40)* | 4 (3, 7.2) | 59.2* | 30 (25, 40) | 56.5 (11.6, 79.2)* | 2 (2, 3) |
| Service workers | 139 (18) | 20 (6, 39)* | 5 (3, 10)* | 46* | 30 (24.2, 40) | 37.2 (8.6, 68.6)* | 2 (2, 3)* |
| Professionals | 114 (14.8) | 12 (7, 26)* | 6 (4, 10)* | 31. 6* | 31 (21.5, 40) | 27.1 (10.9, 55)* | 2 (2, 3)* |
| Unemployed | 26 (3.4) | 7 (3, 12.5) | 4 (3, 10) | 3.8 | 100 (100, 100) | 7.6 (5, 11.8)* | 2 (1, 2) |
| Retired | 230 (29.8) | 6 (3, 14.8) | 5 (3, 10)* | 8.7 | 30 (25, 31.2) | 8 (4, 15.5)* | 2 (1, 2) |
| Others[c] | 30 (3.9) | 10 (5, 35)* | 6 (3, 9.8)* | 30 | 40 (30, 60) | 12.2 (8, 55)* | 2 (2, 3)* |
| Unknown | 5 (0.6) | 3 (3, 11) | 3 (3, 7) | 20 | 50 (50, 50) | 12 (6.5, 36.8) | 1 (1, 3) |
| Individual annual income ($) | | | | | | | |
| 1-8,000 | 182 (23.6) | 9 (4, 20) | 6 (3, 10) | 15.4 | 32.5 (29.5, 43.2) | 9.1 (4, 17.8) | 2 (1, 2) |
| 8,001-11,000 | 165 (21.4) | 12 (5, 35)* | 5 (3, 10) | 34.5* | 30 (25, 40) | 20.2 (8, 57.2)* | 2 (2, 3)* |
| 11,001-15,000 | 69 (8.9) | 9 (4, 25) | 5 (4, 10) | 27.5* | 32 (23.5, 40) | 20 (7.7, 53.6)* | 2 (2, 3) |

| | | | | | | | |
|---|---|---|---|---|---|---|---|
| >15,000 | 49 (6.3) | 15 (7, 30)* | 6 (2, 10) | 36.7* | 30 (22.5, 48.8) | 40 (15.5, 57.6)* | 2 (2, 3) |
| No income | 61 (7.9) | 7 (4, 17) | 5 (3, 8)* | 16.4 | 24 (20, 33.8) | 8.1 (5.2, 26.5) | 2 (1, 2) |
| Not available[d] | 217 (28.1) | 15 (4, 35)* | 4 (3, 7)* | 43.8* | 30 (25, 40) | 24.7 (12, 73.5)* | 2 (1, 2) |
| Unknown | 29 (3.8) | 5 (2, 15) | 5 (2, 8)* | 17.2 | 25 (20, 25) | 8 (5.1, 16) | 2 (1, 2) |
| Years of living in Shanghai | | | | | | | |
| 0-10 years | 77 (10) | 11 (5, 37) | 5 (2, 10) | 37.7 | 35 (27, 38) | 16 (8, 62.9) | 2 (2, 2) |
| >10 years | 579 (75) | 10 (5, 30.5) | 5 (3, 10) | 29.7 | 30 (25, 40) | 14.8 (6.5, 53.6) | 2 (2, 3) |
| Not available[e] | 114 (14.8) | 7 (4, 27.8) | 4 (3, 7.8) | 27.2 | 30 (25, 35.5) | 16 (10.5, 48.8) | 2 (1, 2) |
| Unknown | 2 (0.3) | 9.5 (6.8, 12.2) | 4.5 (4.2, 4.8) | 0 | - | 4.5 (3, 6) | 1.5 (1.2, 1.8) |
| Household size | | | | | | | |
| 1-2 | 231 (29.9) | 8 (3, 20) | 5 (3, 10) | 17.7 | 30 (30, 40) | 8.9 (4, 26) | 2 (1, 2) |
| 3-4 | 427 (55.3) | 12 (4.5, 33)* | 5 (3, 8) | 36.5* | 30 (25, 40) | 16.9 (8.1, 60.8)* | 2 (1, 3) |
| >4 | 114 (14.8) | 10.5 (5, 31)* | 6 (4, 10) | 30.7* | 33 (25, 40) | 17.4 (11.2, 62.5)* | 2 (1, 2.8) |
| Mode of data collection | | | | | | | |
| Self-reporting | 386 (50) | 10 (4, 30) | 5 (3, 10) | 27.2 | 30 (25, 38) | 13.5 (7.2, 52.5) | 2 (1, 3) |
| Telephone interview | 386 (50) | 10 (5, 31) | 5 (3, 8)* | 32.9* | 33 (25, 40) | 16.3 (8, 55.4)* | 2 (2, 2) |
| Population density | | | | | | | |
| Low | 151 (19.6) | 6 (3.5, 26.5) | 4 (3, 6) | 26.5 | 35 (27.8, 42.5) | 12 (8, 48.3) | 2 (1, 2) |
| Moderate | 334 (43.3) | 10 (5, 29) | 5 (3, 10)* | 30.2 | 30 (25, 40) | 16 (8, 49.1) | 2 (1, 3)* |
| High | 287 (37.2) | 12 (5, 33.5)* | 5 (3, 10.5)* | 31.7* | 30 (25, 40) | 16 (7, 58.2)* | 2 (1, 3)* |
| Day type | | | | | | | |
| Weekday | 580 (75.1) | 12.5 (5, 32) | 5 (3, 10) | 34 | 30 (25, 40) | 16.1 (8, 57.6) | 2 (2, 3) |
| Weekend | 192 (24.9) | 7 (4, 19)* | 5 (3, 9.2) | 18.2* | 30 (28.5, 40) | 10.8 (5.6, 26.1)* | 2 (1, 2) |
| Typical day | | | | | | | |
| Yes | 655 (84.8) | 10 (4, 31) | 5 (3, 9) | 30.8 | 30 (25, 40) | 14 (7.4, 55.2) | 2 (1, 2.5) |
| No | 94 (12.2) | 14.5 (6, 28.8) | 7 (4, 12) | 28.7 | 30 (22.5, 40) | 22 (9.6, 52.5) | 2 (2, 3)* |
| Unknown | 23 (3) | 6 (4, 21.5) | 5 (4, 11.5)* | 13* | 25 (25, 36.5) | 9 (3.9, 22.4) | 2 (1, 3) |
| Travel outside the subdistrict | | | | | | | |
| Almost daily | 446 (57.8) | 13 (5, 33) | 5 (3, 9.8) | 36.5 | 30 (25, 40) | 21.5 (8.1, 59.9) | 2 (2, 3) |
| Not daily | 300 (38.9) | 8 (4, 20.2)* | 5 (3, 10) | 20.7* | 30 (25, 40) | 11.7 (5.9, 31.9)* | 2 (1, 2) |
| Unknown | 26 (3.4) | 7.5 (4.2, 21)* | 5 (3.2, 6)* | 26.9 | 25 (20.5, 32.5) | 5.7 (2.1, 30)* | 2 (1, 2) |
| Animal ownership | | | | | | | |
| Owner | 131 (17) | 17 (5, 36.5) | 5 (3, 10) | 38.9 | 30 (25, 40) | 17.7 (8.5, 61.5) | 2 (2, 3) |
| Not owner | 641 (83) | 10 (4, 29)* | 5 (3, 10) | 28.2* | 30 (25, 40) | 14.2 (7.3, 52.5) | 2 (1, 3) |

[a]Data presented as no. (%) of respondents; percentages may not total 100 because of rounding.

[b]Median of group contacts for participants reporting a group contact.

[c]Most are freelances.

[d]Students or pre-school children who don't have individual income.

[e]Indicates children younger than 10 years old, as their years of living in Shanghai cannot exceed 10 years.

*Indicates statistically significant difference from the first level of each factor in the univariate regression model (at the 5% level).

**Table S9.** Estimates contribution percentage in the predicted number of total contacts, individual contacts, probability of reporting group contact, contact duration, number of contact settings, probability of animal ownership and animal contact.

| Characteristics | Number of total contacts | Number of individual contacts | Probability of reporting group contact | Contact duration | Number of settings | Probability of animal ownership | Probability of animal contact |
|---|---|---|---|---|---|---|---|
| Mode of data collection | | | | | | | |
| Self-reporting | 0 (-27.1, 27.1) | 0 (-32.5, 32.5) | 0 (-48.4, 48.4) | 0 (-27, 27) | 0 (-8, 8) | - | - |
| Telephone interview | 23.9 (-9.2, 57.1)* | 13.2 (-23, 49.5) | 39.8 (-16.5, 96.1)* | 20.8 (-10.7, 52.3)* | -0.7 (-8.4, 7) | - | - |
| Household size | | | | | | | |
| 1-2 | - | 0 (-32.5, 32.5) | - | 0 (-27, 27) | - | - | - |
| 3-4 | - | 9.4 (-25.1, 43.9) | - | 21.3 (-7.1, 49.7)* | - | - | - |
| >4 | - | 29.3 (-15.2, 73.9)* | - | 31.1 (-9.1, 71.2)* | - | - | - |
| Day type | | | | | | | |
| Weekday | 0 (-27.1, 27.1) | - | 0 (-48.4, 48.4) | 0 (-27, 27) | 0 (-8, 8) | - | - |
| Weekend | -25.6 (-47.6, -3.6)* | - | -54 (-84.7, -23.4)* | -32.1 (-52.2, -12.1)* | -9.3 (-17.3, -1.3)* | - | - |
| Travel outside the subdistrict | | | | | | | |
| Almost daily | 0 (-27.1, 27.1) | - | - | 0 (-27, 27) | 0 (-8, 8) | - | - |
| Not daily | -15.1 (-39.7, 9.6)* | - | - | -13 (-37.6, 11.6) | -3.5 (-11.8, 4.8) | - | - |
| Unknown | -29.6 (-60.8, 1.6) | - | - | -41.5 (-68.2, -14.8)* | -11.1 (-23.2, 1.1)* | - | - |
| Population density | | | | | | | |
| Low | - | - | 0 (-48.4, 48.4) | - | - | - | - |
| Moderate | - | - | 43.6 (-3.6, 90.9)* | - | - | - | - |
| High | - | - | 78.6 (24, 133.1)* | - | - | - | - |
| Typical day | | | | | | | |
| Yes | - | - | - | - | 0 (-8, 8) | - | - |
| No | - | - | - | - | 9.5 (-1.3, 20.2) * | - | - |
| Unknown | - | - | - | - | -0.7 (-15.3, 13.9) | - | - |

| | | | | | | | |
|---|---|---|---|---|---|---|---|
| Animal ownership | | | | | | | |
| Owner | 0 (-27.1, 27.1) | - | - | - | - | - | - |
| Not owner | -15.4 (-35.3, 4.5)* | - | - | - | - | - | - |
| Individual annual income ($) | | | | | | | |
| 1-8,000 | - | - | - | - | - | 0 (-46.2, 46.2) | 0 (-44.5, 44.5) |
| 8,001-11,000 | - | - | - | - | - | -24.5 (-57.6, 8.5) | -31.8 (-61.6, -2) |
| 11,001-15,000 | - | - | - | - | - | -42.9 (-76.8, -8.9)* | -42.5 (-75.7, -9.3)* |
| >15,000 | - | - | - | - | - | 21.3 (-28.7, 71.3) | 3.1 (-40.7, 47) |
| No income | - | - | - | - | - | -58.7 (-91.8, -25.7)* | -69.7 (-96, -43.4)* |
| Not available | - | - | - | - | - | -61.5 (-98.4, -24.6)* | -56.9 (-97.1, -16.7)* |
| Unknown | - | - | - | - | - | -79.3 (-108.9, -49.7)* | -74.9 (-106.3, -43.5)* |

*Indicates statistically significant difference from the first level of each factor in the univariate regression model (at the 5% level).

**Table S10a.** Original contact matrix of all reported contacts consisting of the average number of contact persons recorded per day per survey participant.

| | | Age of contact | | | | | | | | | | | | | | | | |
|---|---|---|---|---|---|---|---|---|---|---|---|---|---|---|---|---|---|---|
| | | **0-2** | **3-6** | **7-9** | **10-14** | **15-19** | **20-24** | **25-29** | **30-34** | **35-39** | **40-44** | **45-49** | **50-54** | **55-59** | **60-64** | **65-69** | **70-74** | **75+** |
| **Age of participant** | **0-2** | 0.64 | 0.33 | 0.00 | 0.06 | 0.03 | 0.03 | 0.94 | 1.03 | 0.72 | 0.08 | 0.03 | 0.19 | 0.50 | 0.72 | 0.58 | 0.14 | 0.17 |
| | **3-6** | 1.29 | 2.85 | 1.73 | 0.44 | 0.08 | 0.44 | 0.98 | 1.83 | 1.33 | 0.58 | 0.42 | 0.50 | 0.92 | 0.52 | 0.27 | 0.06 | 0.04 |
| | **7-9** | 0.04 | 2.04 | 9.00 | 3.38 | 0.21 | 0.21 | 0.58 | 1.08 | 1.58 | 0.88 | 0.46 | 0.12 | 0.46 | 0.54 | 0.92 | 0.33 | 0.12 |
| | **10-14** | 0.00 | 0.55 | 2.87 | 9.55 | 6.25 | 0.30 | 0.34 | 0.79 | 1.24 | 1.27 | 0.60 | 0.36 | 0.19 | 0.25 | 0.58 | 0.12 | 0.33 |
| | **15-19** | 0.00 | 0.00 | 0.05 | 6.70 | 7.35 | 1.77 | 1.52 | 1.90 | 3.50 | 3.52 | 2.30 | 1.30 | 1.27 | 0.30 | 0.25 | 0.10 | 0.62 |
| | **20-24** | 0.11 | 0.04 | 0.04 | 0.07 | 0.32 | 2.75 | 3.96 | 3.29 | 3.64 | 2.64 | 2.89 | 2.32 | 2.32 | 0.36 | 0.07 | 0.07 | 0.25 |
| | **25-29** | 0.05 | 0.15 | 0.00 | 0.05 | 0.17 | 1.93 | 3.07 | 2.34 | 2.51 | 1.88 | 1.98 | 1.44 | 2.17 | 0.51 | 0.20 | 0.07 | 0.07 |
| | **30-34** | 0.17 | 0.71 | 0.12 | 0.10 | 0.27 | 1.56 | 3.17 | 4.69 | 4.60 | 2.19 | 2.27 | 1.77 | 1.98 | 0.85 | 0.29 | 0.15 | 0.17 |
| | **35-39** | 0.02 | 0.16 | 0.09 | 0.56 | 0.14 | 1.21 | 1.84 | 2.65 | 3.09 | 1.70 | 1.84 | 1.33 | 1.33 | 0.72 | 0.42 | 0.28 | 0.14 |
| | **40-44** | 0.00 | 0.00 | 0.12 | 0.39 | 0.33 | 0.88 | 1.06 | 1.55 | 3.06 | 2.76 | 3.94 | 2.06 | 1.76 | 0.33 | 0.73 | 0.52 | 0.30 |
| | **45-49** | 0.00 | 0.07 | 0.13 | 0.38 | 0.31 | 0.89 | 1.22 | 1.64 | 2.27 | 2.15 | 3.22 | 2.07 | 1.76 | 0.87 | 0.76 | 0.80 | 1.02 |
| | **50-54** | 0.03 | 0.08 | 0.06 | 0.11 | 0.11 | 0.50 | 1.39 | 1.03 | 1.53 | 2.22 | 2.53 | 2.14 | 1.58 | 1.19 | 0.61 | 0.78 | 1.31 |
| | **55-59** | 0.02 | 0.08 | 0.04 | 0.16 | 0.04 | 0.49 | 1.14 | 1.14 | 1.69 | 1.63 | 1.73 | 1.45 | 2.22 | 1.43 | 0.75 | 1.29 | 1.22 |
| | **60-64** | 0.00 | 0.05 | 0.02 | 0.10 | 0.03 | 0.18 | 0.51 | 0.67 | 1.18 | 0.64 | 0.56 | 1.15 | 1.69 | 1.89 | 1.36 | 0.75 | 0.80 |
| | **65-69** | 0.02 | 0.12 | 0.02 | 0.06 | 0.02 | 0.10 | 0.27 | 0.37 | 0.98 | 0.88 | 0.88 | 0.78 | 1.51 | 2.10 | 3.65 | 1.20 | 1.41 |
| | **70-74** | 0.02 | 0.02 | 0.18 | 0.09 | 0.06 | 0.09 | 0.31 | 0.25 | 0.54 | 0.68 | 0.75 | 0.95 | 0.78 | 1.52 | 1.86 | 1.62 | 1.43 |
| | **75+** | 0.00 | 0.02 | 0.00 | 0.02 | 0.04 | 0.11 | 0.24 | 0.04 | 0.18 | 0.36 | 0.47 | 0.40 | 0.71 | 1.27 | 1.04 | 1.07 | 3.80 |

**Table S10b.** Original contact matrix of reported contacts derived from self-reporting, consisting of the average number of contact persons recorded per day per survey participant.

| | | Age of contact | | | | | | | | | | | | | | | | |
|---|---|---|---|---|---|---|---|---|---|---|---|---|---|---|---|---|---|---|
| | | **0-2** | **3-6** | **7-9** | **10-14** | **15-19** | **20-24** | **25-29** | **30-34** | **35-39** | **40-44** | **45-49** | **50-54** | **55-59** | **60-64** | **65-69** | **70-74** | **75+** |
| **Age of participant** | **0-2** | 0.79 | 0.41 | 0.00 | 0.07 | 0.03 | 0.03 | 0.66 | 1.07 | 0.55 | 0.10 | 0.03 | 0.14 | 0.45 | 0.83 | 0.55 | 0.17 | 0.21 |
| | **3-6** | 1.56 | 2.67 | 1.11 | 0.41 | 0.07 | 0.22 | 0.67 | 1.63 | 1.07 | 0.56 | 0.48 | 0.37 | 0.56 | 0.37 | 0.33 | 0.07 | 0.04 |
| | **7-9** | 0.09 | 2.64 | 9.55 | 4.27 | 0.36 | 0.18 | 0.82 | 1.18 | 1.27 | 1.36 | 0.45 | 0.27 | 0.27 | 0.45 | 0.45 | 0.18 | 0.09 |
| | **10-14** | 0.00 | 0.87 | 4.16 | 13.35 | 8.55 | 0.13 | 0.03 | 0.58 | 1.06 | 0.97 | 0.42 | 0.35 | 0.03 | 0.29 | 0.55 | 0.23 | 0.29 |
| | **15-19** | 0.00 | 0.00 | 0.10 | 6.85 | 8.60 | 1.40 | 1.50 | 1.50 | 3.05 | 3.35 | 1.65 | 1.45 | 0.95 | 0.50 | 0.20 | 0.15 | 0.90 |
| | **20-24** | 0.20 | 0.00 | 0.00 | 0.07 | 0.27 | 3.73 | 4.07 | 4.00 | 4.00 | 2.53 | 3.47 | 2.67 | 2.93 | 0.53 | 0.13 | 0.07 | 0.27 |
| | **25-29** | 0.11 | 0.16 | 0.00 | 0.11 | 0.16 | 1.74 | 2.47 | 1.58 | 2.53 | 1.74 | 1.37 | 1.37 | 2.42 | 0.58 | 0.16 | 0.11 | 0.05 |
| | **30-34** | 0.14 | 0.57 | 0.00 | 0.05 | 0.14 | 0.76 | 1.95 | 3.24 | 2.86 | 1.48 | 1.38 | 1.24 | 1.29 | 1.10 | 0.33 | 0.24 | 0.24 |
| | **35-39** | 0.05 | 0.14 | 0.14 | 0.43 | 0.14 | 1.05 | 2.00 | 2.71 | 3.38 | 1.52 | 1.90 | 1.48 | 1.52 | 0.67 | 0.57 | 0.38 | 0.24 |
| | **40-44** | 0.00 | 0.00 | 0.12 | 0.37 | 0.31 | 1.06 | 1.31 | 2.12 | 3.25 | 2.56 | 3.19 | 2.62 | 2.19 | 0.44 | 0.75 | 0.56 | 0.12 |
| | **45-49** | 0.00 | 0.00 | 0.16 | 0.32 | 0.37 | 0.84 | 1.16 | 1.47 | 2.00 | 2.32 | 2.95 | 2.32 | 1.58 | 0.37 | 0.47 | 0.42 | 0.58 |
| | **50-54** | 0.00 | 0.00 | 0.06 | 0.11 | 0.06 | 0.33 | 1.00 | 0.56 | 1.22 | 0.94 | 1.00 | 1.67 | 1.28 | 0.33 | 0.06 | 0.17 | 0.22 |
| | **55-59** | 0.00 | 0.00 | 0.00 | 0.08 | 0.04 | 0.35 | 1.08 | 0.62 | 1.19 | 1.27 | 1.35 | 1.35 | 1.73 | 1.31 | 0.54 | 2.19 | 1.81 |
| | **60-64** | 0.00 | 0.14 | 0.04 | 0.00 | 0.00 | 0.14 | 0.61 | 0.89 | 1.29 | 0.82 | 0.46 | 1.46 | 1.71 | 1.64 | 1.14 | 0.25 | 0.68 |
| | **65-69** | 0.00 | 0.24 | 0.07 | 0.07 | 0.03 | 0.17 | 0.31 | 0.52 | 1.14 | 1.00 | 0.66 | 1.17 | 1.59 | 2.38 | 2.38 | 1.31 | 1.21 |
| | **70-74** | 0.03 | 0.03 | 0.21 | 0.09 | 0.03 | 0.09 | 0.21 | 0.36 | 0.55 | 0.61 | 1.09 | 1.30 | 1.39 | 1.94 | 2.12 | 2.00 | 1.45 |
| | **75+** | 0.00 | 0.04 | 0.00 | 0.09 | 0.09 | 0.22 | 0.04 | 0.09 | 0.04 | 0.52 | 0.48 | 0.74 | 0.87 | 1.61 | 1.35 | 1.30 | 3.30 |

**Table S10c**. Original contact matrix of reported contacts derived from telephone interview, consisting of the average number of contact persons recorded per day per survey participant.

| | | **Age of contact** | | | | | | | | | | | | | | | | |
|---|---|---|---|---|---|---|---|---|---|---|---|---|---|---|---|---|---|---|
| | | **0-2** | **3-6** | **7-9** | **10-14** | **15-19** | **20-24** | **25-29** | **30-34** | **35-39** | **40-44** | **45-49** | **50-54** | **55-59** | **60-64** | **65-69** | **70-74** | **75+** |
| **Age of participant** | **0-2** | 0.00 | 0.00 | 0.00 | 0.00 | 0.00 | 0.00 | 2.14 | 0.86 | 1.43 | 0.00 | 0.00 | 0.43 | 0.71 | 0.29 | 0.71 | 0.00 | 0.00 |
| | **3-6** | 0.95 | 3.10 | 2.48 | 0.48 | 0.10 | 0.71 | 1.38 | 2.14 | 1.67 | 0.62 | 0.33 | 0.67 | 1.38 | 0.71 | 0.19 | 0.05 | 0.05 |
| | **7-9** | 0.00 | 1.69 | 8.46 | 2.69 | 0.08 | 0.23 | 0.31 | 1.00 | 1.77 | 0.54 | 0.46 | 0.00 | 0.62 | 0.62 | 1.31 | 0.38 | 0.15 |
| | **10-14** | 0.00 | 0.28 | 1.72 | 6.28 | 4.28 | 0.44 | 0.61 | 0.97 | 1.39 | 1.53 | 0.81 | 0.36 | 0.33 | 0.22 | 0.61 | 0.03 | 0.33 |
| | **15-19** | 0.00 | 0.00 | 0.00 | 6.55 | 6.10 | 2.15 | 1.55 | 2.30 | 3.95 | 3.70 | 2.95 | 1.15 | 1.60 | 0.10 | 0.30 | 0.05 | 0.35 |
| | **20-24** | 0.08 | 0.08 | 0.08 | 0.08 | 0.38 | 1.46 | 3.85 | 2.46 | 3.23 | 2.62 | 2.38 | 1.92 | 1.62 | 0.23 | 0.00 | 0.08 | 0.23 |
| | **25-29** | 0.00 | 0.14 | 0.00 | 0.00 | 0.18 | 2.05 | 3.50 | 3.05 | 2.68 | 2.00 | 2.41 | 1.50 | 1.95 | 0.45 | 0.23 | 0.05 | 0.09 |
| | **30-34** | 0.19 | 0.78 | 0.22 | 0.15 | 0.37 | 2.15 | 4.11 | 5.78 | 6.07 | 2.70 | 2.96 | 2.19 | 2.48 | 0.74 | 0.26 | 0.07 | 0.11 |
| | **35-39** | 0.00 | 0.18 | 0.05 | 0.59 | 0.14 | 1.45 | 1.77 | 2.50 | 2.82 | 1.86 | 1.68 | 1.23 | 1.14 | 0.73 | 0.32 | 0.18 | 0.09 |
| | **40-44** | 0.00 | 0.00 | 0.12 | 0.53 | 0.35 | 0.59 | 0.71 | 1.06 | 3.00 | 3.00 | 4.47 | 1.65 | 1.29 | 0.24 | 0.71 | 0.53 | 0.41 |
| | **45-49** | 0.00 | 0.08 | 0.11 | 0.42 | 0.33 | 0.86 | 1.19 | 1.72 | 2.39 | 2.06 | 3.53 | 1.97 | 1.81 | 1.11 | 0.92 | 1.03 | 1.22 |
| | **50-54** | 0.06 | 0.17 | 0.06 | 0.11 | 0.17 | 0.67 | 1.72 | 1.50 | 1.83 | 3.56 | 4.06 | 2.67 | 1.89 | 2.06 | 1.11 | 1.33 | 2.44 |
| | **55-59** | 0.04 | 0.16 | 0.08 | 0.24 | 0.04 | 0.68 | 1.20 | 1.56 | 2.16 | 2.04 | 2.24 | 1.52 | 2.68 | 1.48 | 0.96 | 0.40 | 0.68 |
| | **60-64** | 0.00 | 0.03 | 0.00 | 0.18 | 0.06 | 0.18 | 0.48 | 0.55 | 1.21 | 0.55 | 0.61 | 1.00 | 1.64 | 1.94 | 1.42 | 1.06 | 0.91 |
| | **65-69** | 0.05 | 0.00 | 0.00 | 0.05 | 0.00 | 0.05 | 0.23 | 0.23 | 0.68 | 0.86 | 1.32 | 0.27 | 1.68 | 1.59 | 4.95 | 1.00 | 1.59 |
| | **70-74** | 0.00 | 0.00 | 0.16 | 0.06 | 0.09 | 0.12 | 0.41 | 0.12 | 0.53 | 0.78 | 0.41 | 0.53 | 0.22 | 0.97 | 1.53 | 1.25 | 1.53 |
| | **75+** | 0.00 | 0.00 | 0.00 | 0.00 | 0.00 | 0.00 | 0.32 | 0.00 | 0.32 | 0.09 | 0.41 | 0.18 | 0.55 | 0.77 | 0.77 | 0.91 | 4.41 |

**Table S10d.** Original contact matrix (equal 5-year age bands) of all reported contacts consisting of the average number of contact persons recorded per day per survey participant.

| | | Age of contact | | | | | | | | | | | | | | | |
|---|---|---|---|---|---|---|---|---|---|---|---|---|---|---|---|---|---|
| | | **0-4** | **5-9** | **10-14** | **15-19** | **20-24** | **25-29** | **30-34** | **35-39** | **40-44** | **45-49** | **50-54** | **55-59** | **60-64** | **65-69** | **70-74** | **75+** |
| **Age of participant** | **0-4** | 2.10 | 0.11 | 0.06 | 0.06 | 0.22 | 1.06 | 1.43 | 0.92 | 0.37 | 0.16 | 0.44 | 0.79 | 0.57 | 0.38 | 0.11 | 0.11 |
| | **5-9** | 1.18 | 8.62 | 2.24 | 0.13 | 0.29 | 0.67 | 1.33 | 1.56 | 0.64 | 0.49 | 0.13 | 0.51 | 0.67 | 0.71 | 0.18 | 0.09 |
| | **10-14** | 0.00 | 3.39 | 9.61 | 6.27 | 0.30 | 0.34 | 0.79 | 1.24 | 1.24 | 0.60 | 0.36 | 0.19 | 0.25 | 0.58 | 0.12 | 0.31 |
| | **15-19** | 0.00 | 0.05 | 6.70 | 7.35 | 1.77 | 1.52 | 1.90 | 3.50 | 3.52 | 2.30 | 1.30 | 1.27 | 0.30 | 0.25 | 0.10 | 0.62 |
| | **20-24** | 0.14 | 0.04 | 0.07 | 0.32 | 2.64 | 3.96 | 3.32 | 3.68 | 2.64 | 3.00 | 2.25 | 2.29 | 0.36 | 0.07 | 0.11 | 0.25 |
| | **25-29** | 0.15 | 0.05 | 0.05 | 0.17 | 1.93 | 3.07 | 2.39 | 2.51 | 1.88 | 1.93 | 1.46 | 2.15 | 0.51 | 0.20 | 0.07 | 0.07 |
| | **30-34** | 0.50 | 0.48 | 0.10 | 0.27 | 1.58 | 3.19 | 4.62 | 4.65 | 2.15 | 2.29 | 1.75 | 1.98 | 0.88 | 0.29 | 0.17 | 0.17 |
| | **35-39** | 0.07 | 0.21 | 0.56 | 0.16 | 1.16 | 1.79 | 2.70 | 3.19 | 1.77 | 1.77 | 1.33 | 1.26 | 0.72 | 0.42 | 0.28 | 0.14 |
| | **40-44** | 0.00 | 0.12 | 0.39 | 0.30 | 0.79 | 1.06 | 1.55 | 3.03 | 2.70 | 4.06 | 2.00 | 1.79 | 0.36 | 0.85 | 0.52 | 0.27 |
| | **45-49** | 0.04 | 0.15 | 0.38 | 0.31 | 0.91 | 1.18 | 1.65 | 2.24 | 2.16 | 3.31 | 2.07 | 1.73 | 0.87 | 0.76 | 0.82 | 0.98 |
| | **50-54** | 0.11 | 0.06 | 0.11 | 0.11 | 0.50 | 1.36 | 1.03 | 1.53 | 2.25 | 2.53 | 2.17 | 1.58 | 1.22 | 0.58 | 0.75 | 1.31 |
| | **55-59** | 0.06 | 0.08 | 0.16 | 0.04 | 0.47 | 1.12 | 1.06 | 1.69 | 1.67 | 1.73 | 1.45 | 2.25 | 1.41 | 0.76 | 1.31 | 1.24 |
| | **60-64** | 0.03 | 0.05 | 0.13 | 0.03 | 0.16 | 0.52 | 0.72 | 1.18 | 0.67 | 0.54 | 1.23 | 1.64 | 1.82 | 1.26 | 0.77 | 0.80 |
| | **65-69** | 0.08 | 0.08 | 0.06 | 0.02 | 0.10 | 0.29 | 0.43 | 0.94 | 0.88 | 0.94 | 0.78 | 1.51 | 2.08 | 3.63 | 1.18 | 1.37 |
| | **70-74** | 0.02 | 0.22 | 0.08 | 0.06 | 0.09 | 0.31 | 0.25 | 0.52 | 0.69 | 0.77 | 0.95 | 0.78 | 1.46 | 1.86 | 1.63 | 1.46 |
| | **75+** | 0.00 | 0.02 | 0.02 | 0.07 | 0.11 | 0.18 | 0.07 | 0.24 | 0.33 | 0.42 | 0.40 | 0.64 | 1.20 | 1.16 | 1.09 | 3.82 |

**Table S10e.** Original contact matrix (equal 5-year age bands) of reported contacts derived from self-reporting, consisting of the average number of contact persons recorded per day per survey participant.

| | | **Age of contact** | | | | | | | | | | | | | | | |
|---|---|---|---|---|---|---|---|---|---|---|---|---|---|---|---|---|---|
| | | **0-4** | **5-9** | **10-14** | **15-19** | **20-24** | **25-29** | **30-34** | **35-39** | **40-44** | **45-49** | **50-54** | **55-59** | **60-64** | **65-69** | **70-74** | **75+** |
| **Age of participant** | **0-4** | 2.04 | 0.11 | 0.07 | 0.04 | 0.09 | 0.67 | 1.24 | 0.58 | 0.29 | 0.16 | 0.27 | 0.47 | 0.62 | 0.42 | 0.16 | 0.16 |
| | **5-9** | 1.59 | 8.14 | 2.59 | 0.23 | 0.23 | 0.77 | 1.45 | 1.55 | 0.91 | 0.55 | 0.23 | 0.45 | 0.50 | 0.55 | 0.09 | 0.05 |
| | **10-14** | 0.00 | 5.10 | 13.35 | 8.58 | 0.13 | 0.03 | 0.58 | 1.06 | 0.94 | 0.35 | 0.35 | 0.03 | 0.29 | 0.55 | 0.23 | 0.29 |
| | **15-19** | 0.00 | 0.10 | 6.85 | 8.60 | 1.40 | 1.50 | 1.50 | 3.05 | 3.35 | 1.65 | 1.45 | 0.95 | 0.50 | 0.20 | 0.15 | 0.90 |
| | **20-24** | 0.13 | 0.00 | 0.07 | 0.27 | 3.80 | 4.13 | 4.00 | 4.13 | 2.53 | 3.40 | 2.67 | 2.80 | 0.53 | 0.13 | 0.07 | 0.27 |
| | **25-29** | 0.21 | 0.05 | 0.11 | 0.16 | 1.79 | 2.53 | 1.58 | 2.42 | 1.68 | 1.42 | 1.42 | 2.37 | 0.58 | 0.16 | 0.11 | 0.05 |
| | **30-34** | 0.29 | 0.38 | 0.05 | 0.19 | 0.76 | 1.90 | 3.33 | 2.76 | 1.48 | 1.43 | 1.19 | 1.38 | 1.05 | 0.33 | 0.24 | 0.24 |
| | **35-39** | 0.14 | 0.29 | 0.48 | 0.14 | 0.95 | 1.76 | 2.76 | 3.62 | 1.52 | 1.90 | 1.43 | 1.33 | 0.71 | 0.52 | 0.48 | 0.29 |
| | **40-44** | 0.00 | 0.12 | 0.25 | 0.25 | 1.06 | 1.62 | 2.06 | 3.12 | 2.56 | 3.19 | 2.62 | 2.19 | 0.44 | 0.75 | 0.62 | 0.12 |
| | **45-49** | 0.00 | 0.16 | 0.32 | 0.26 | 0.89 | 1.16 | 1.53 | 2.00 | 2.37 | 2.95 | 2.21 | 1.58 | 0.37 | 0.47 | 0.42 | 0.63 |
| | **50-54** | 0.00 | 0.06 | 0.11 | 0.06 | 0.33 | 1.00 | 0.56 | 1.22 | 0.94 | 1.00 | 1.67 | 1.28 | 0.33 | 0.06 | 0.17 | 0.22 |
| | **55-59** | 0.00 | 0.00 | 0.08 | 0.04 | 0.35 | 1.00 | 0.62 | 1.15 | 1.31 | 1.23 | 1.35 | 1.77 | 1.42 | 0.58 | 2.23 | 1.77 |
| | **60-64** | 0.04 | 0.11 | 0.00 | 0.00 | 0.14 | 0.54 | 0.89 | 1.21 | 0.71 | 0.46 | 1.61 | 1.64 | 1.82 | 1.07 | 0.36 | 0.68 |
| | **65-69** | 0.10 | 0.17 | 0.07 | 0.03 | 0.14 | 0.31 | 0.52 | 1.28 | 0.97 | 0.59 | 1.17 | 1.41 | 2.55 | 2.38 | 1.28 | 1.28 |
| | **70-74** | 0.03 | 0.24 | 0.09 | 0.03 | 0.06 | 0.21 | 0.36 | 0.52 | 0.55 | 1.12 | 1.33 | 1.36 | 1.94 | 2.18 | 2.03 | 1.45 |
| | **75+** | 0.00 | 0.04 | 0.04 | 0.09 | 0.22 | 0.04 | 0.09 | 0.04 | 0.57 | 0.48 | 0.74 | 0.78 | 1.57 | 1.30 | 1.35 | 3.43 |

**Table S10f**. Original contact matrix (equal 5-year age bands) of reported contacts derived from telephone interview, consisting of the average number of contact persons recorded per day per survey participant.

| | | **Age of contact** | | | | | | | | | | | | | | | |
|---|---|---|---|---|---|---|---|---|---|---|---|---|---|---|---|---|---|
| | | **0-4** | **5-9** | **10-14** | **15-19** | **20-24** | **25-29** | **30-34** | **35-39** | **40-44** | **45-49** | **50-54** | **55-59** | **60-64** | **65-69** | **70-74** | **75+** |
| **Age of participant** | **0-4** | 2.22 | 0.11 | 0.06 | 0.11 | 0.56 | 2.06 | 1.89 | 1.78 | 0.56 | 0.17 | 0.89 | 1.61 | 0.44 | 0.28 | 0.00 | 0.00 |
| | **5-9** | 0.83 | 9.04 | 1.91 | 0.04 | 0.35 | 0.48 | 1.30 | 1.57 | 0.43 | 0.43 | 0.04 | 0.57 | 0.74 | 0.91 | 0.26 | 0.13 |
| | **10-14** | 0.00 | 2.00 | 6.31 | 4.28 | 0.44 | 0.61 | 0.97 | 1.39 | 1.50 | 0.81 | 0.36 | 0.33 | 0.22 | 0.61 | 0.03 | 0.33 |
| | **15-19** | 0.00 | 0.00 | 6.55 | 6.10 | 2.15 | 1.55 | 2.30 | 3.95 | 3.70 | 2.95 | 1.15 | 1.60 | 0.10 | 0.30 | 0.05 | 0.35 |
| | **20-24** | 0.15 | 0.08 | 0.08 | 0.38 | 1.46 | 3.92 | 2.46 | 3.23 | 2.62 | 2.38 | 1.85 | 1.62 | 0.23 | 0.00 | 0.08 | 0.23 |
| | **25-29** | 0.09 | 0.05 | 0.00 | 0.18 | 2.05 | 3.50 | 3.05 | 2.68 | 2.00 | 2.41 | 1.50 | 1.95 | 0.45 | 0.23 | 0.05 | 0.09 |
| | **30-34** | 0.67 | 0.52 | 0.15 | 0.37 | 2.15 | 4.11 | 5.78 | 6.07 | 2.70 | 2.96 | 2.19 | 2.48 | 0.74 | 0.26 | 0.07 | 0.11 |
| | **35-39** | 0.05 | 0.18 | 0.59 | 0.14 | 1.45 | 1.77 | 2.50 | 2.82 | 1.86 | 1.68 | 1.23 | 1.14 | 0.73 | 0.32 | 0.18 | 0.09 |
| | **40-44** | 0.00 | 0.12 | 0.53 | 0.35 | 0.59 | 0.71 | 1.06 | 3.00 | 3.00 | 4.47 | 1.65 | 1.29 | 0.24 | 0.71 | 0.53 | 0.41 |
| | **45-49** | 0.06 | 0.14 | 0.42 | 0.36 | 0.89 | 1.19 | 1.69 | 2.39 | 2.08 | 3.50 | 1.94 | 1.81 | 1.11 | 0.92 | 0.97 | 1.28 |
| | **50-54** | 0.22 | 0.06 | 0.11 | 0.17 | 0.72 | 1.72 | 1.50 | 1.83 | 3.56 | 4.06 | 2.67 | 1.89 | 2.06 | 1.11 | 1.33 | 2.39 |
| | **55-59** | 0.12 | 0.16 | 0.24 | 0.04 | 0.64 | 1.20 | 1.56 | 2.16 | 2.04 | 2.28 | 1.52 | 2.68 | 1.48 | 0.96 | 0.40 | 0.68 |
| | **60-64** | 0.03 | 0.00 | 0.18 | 0.06 | 0.18 | 0.48 | 0.55 | 1.21 | 0.55 | 0.64 | 0.97 | 1.67 | 1.94 | 1.39 | 1.06 | 0.91 |
| | **65-69** | 0.05 | 0.00 | 0.05 | 0.00 | 0.05 | 0.27 | 0.18 | 0.64 | 0.91 | 1.32 | 0.27 | 1.68 | 1.59 | 5.05 | 0.95 | 1.55 |
| | **70-74** | 0.00 | 0.16 | 0.06 | 0.09 | 0.12 | 0.44 | 0.12 | 0.53 | 0.78 | 0.44 | 0.53 | 0.22 | 0.97 | 1.53 | 1.22 | 1.50 |
| | **75+** | 0.00 | 0.00 | 0.00 | 0.00 | 0.00 | 0.32 | 0.00 | 0.32 | 0.09 | 0.41 | 0.18 | 0.55 | 0.77 | 0.77 | 0.91 | 4.41 |

**Table S11.** Human-animal contact matrix of all reported contacts, consisting of the average number of animal contacts per day per participant.

| | | Animal | | | | | | | | | |
|---|---|---|---|---|---|---|---|---|---|---|---|
| | | **Dog** | **Cat** | **Fish** | **Bird** | **Rabbit** | **Rat** | **Chicken** | **Pigeon** | **Sheep** | **Others** |
| **Age of participant** | **0-2** | 0.05 | 0 | 0 | 0 | 0 | 0 | 0 | 0 | 0 | 0 |
| | **3-6** | 0.08 | 0.04 | 0.22 | 0 | 0.02 | 0.06 | 0 | 0 | 0 | 0.08 |
| | **7-9** | 0 | 0.1 | 0 | 0 | 0 | 0 | 0 | 0 | 0 | 0.07 |
| | **10-14** | 0.1 | 0.14 | 0.2 | 0 | 0.01 | 0 | 0 | 0 | 0 | 0 |
| | **15-19** | 0.12 | 0.14 | 0.05 | 0 | 0 | 0 | 0 | 0 | 0 | 0 |
| | **20-24** | 0.22 | 0.22 | 0 | 0 | 0 | 0 | 0 | 0 | 0 | 0.05 |
| | **25-29** | 0.1 | 0.1 | 0 | 0 | 0 | 0 | 0 | 0 | 0 | 0 |
| | **30-34** | 0.48 | 0.09 | 0.3 | 0 | 0.01 | 0 | 0 | 0 | 0 | 0.01 |
| | **35-39** | 0.12 | 0.26 | 0 | 0 | 0 | 0 | 0 | 0 | 0 | 0.1 |
| | **40-44** | 0.08 | 0.16 | 0.86 | 0 | 0 | 0 | 0 | 0 | 0 | 0.02 |
| | **45-49** | 0.17 | 0.06 | 0.08 | 0 | 0 | 0 | 0 | 0 | 0 | 0.01 |
| | **50-54** | 0.1 | 0.08 | 0.04 | 0 | 0 | 0 | 0 | 0 | 0 | 0 |
| | **55-59** | 0.23 | 0.16 | 0.02 | 0.03 | 0 | 0 | 0 | 0 | 0 | 0.02 |
| | **60-64** | 0.25 | 0.09 | 0.01 | 0.1 | 0 | 0 | 0 | 0 | 0 | 0 |
| | **65-69** | 0.06 | 0.06 | 0.05 | 0 | 0 | 0 | 0.03 | 0.03 | 0 | 0 |
| | **70-74** | 0.07 | 0.06 | 0.07 | 0.01 | 0 | 0 | 0 | 0 | 0 | 0.09 |
| | **75+** | 0.12 | 0.04 | 0.02 | 0.02 | 0 | 0 | 0 | 0 | 0 | 0.02 |

**Table S12a**. Original contact matrix of reported contacts for participants owning or touching animals, consisting of the average number of contact persons recorded per day per survey participant.

| | | Age of contact | | | | | | | | | | | | | | | | |
|---|---|---|---|---|---|---|---|---|---|---|---|---|---|---|---|---|---|---|
| | | **0-2** | **3-6** | **7-9** | **10-14** | **15-19** | **20-24** | **25-29** | **30-34** | **35-39** | **40-44** | **45-49** | **50-54** | **55-59** | **60-64** | **65-69** | **70-74** | **75+** |
| **Age of participant** | **0-2** | 0.00 | 0.50 | 0.00 | 0.00 | 0.00 | 0.00 | 0.50 | 1.50 | 0.00 | 0.00 | 0.00 | 0.50 | 0.50 | 0.00 | 0.00 | 0.00 | 0.00 |
| | **3-6** | 3.10 | 3.80 | 0.20 | 0.20 | 0.10 | 0.40 | 0.90 | 1.70 | 1.70 | 0.90 | 0.80 | 0.40 | 0.90 | 0.50 | 0.50 | 0.10 | 0.00 |
| | **7-9** | 0.00 | 3.50 | 9.50 | 4.00 | 0.50 | 1.00 | 0.75 | 2.25 | 3.75 | 3.00 | 1.75 | 0.25 | 1.75 | 0.25 | 0.50 | 0.50 | 0.25 |
| | **10-14** | 0.00 | 0.75 | 3.00 | 10.56 | 5.81 | 0.50 | 0.25 | 0.81 | 1.25 | 1.94 | 0.38 | 0.50 | 0.25 | 0.31 | 0.75 | 0.12 | 0.62 |
| | **15-19** | 0.00 | 0.00 | 0.00 | 5.55 | 5.91 | 1.09 | 0.73 | 1.18 | 2.00 | 3.73 | 1.36 | 1.18 | 0.91 | 0.18 | 0.00 | 0.00 | 0.27 |
| | **20-24** | 0.00 | 0.08 | 0.08 | 0.08 | 0.33 | 1.75 | 3.67 | 2.42 | 3.42 | 2.75 | 2.08 | 1.67 | 1.83 | 0.25 | 0.08 | 0.08 | 0.17 |
| | **25-29** | 0.22 | 0.00 | 0.00 | 0.00 | 0.33 | 2.11 | 2.44 | 3.00 | 2.00 | 2.78 | 2.00 | 1.89 | 2.33 | 0.22 | 0.33 | 0.11 | 0.00 |
| | **30-34** | 0.50 | 0.36 | 0.00 | 0.14 | 0.29 | 2.14 | 3.14 | 5.07 | 6.07 | 3.14 | 3.07 | 3.29 | 2.29 | 0.93 | 0.57 | 0.07 | 0.43 |
| | **35-39** | 0.08 | 0.08 | 0.08 | 0.75 | 0.17 | 1.08 | 1.67 | 3.00 | 2.75 | 1.58 | 1.92 | 1.42 | 0.75 | 0.92 | 0.83 | 0.33 | 0.17 |
| | **40-44** | 0.00 | 0.00 | 0.00 | 0.42 | 0.25 | 1.25 | 2.08 | 1.83 | 3.92 | 3.83 | 3.92 | 3.75 | 3.08 | 0.17 | 0.58 | 0.83 | 0.17 |
| | **45-49** | 0.08 | 0.00 | 0.00 | 0.69 | 0.54 | 1.08 | 2.23 | 2.69 | 3.46 | 3.92 | 4.00 | 3.69 | 3.69 | 0.15 | 0.54 | 0.54 | 0.38 |
| | **50-54** | 0.00 | 0.09 | 0.00 | 0.00 | 0.09 | 0.55 | 1.00 | 0.82 | 1.36 | 2.64 | 2.73 | 2.55 | 1.82 | 0.82 | 0.36 | 0.73 | 1.27 |
| | **55-59** | 0.05 | 0.05 | 0.11 | 0.26 | 0.05 | 0.63 | 1.26 | 1.21 | 2.05 | 2.16 | 2.26 | 1.32 | 2.95 | 1.21 | 0.79 | 0.89 | 0.37 |
| | **60-64** | 0.00 | 0.10 | 0.05 | 0.05 | 0.00 | 0.20 | 0.45 | 0.90 | 1.45 | 0.40 | 0.75 | 1.45 | 2.15 | 2.05 | 1.60 | 1.10 | 0.85 |
| | **65-69** | 0.00 | 0.40 | 0.60 | 0.00 | 0.00 | 0.00 | 0.00 | 0.40 | 1.60 | 0.40 | 0.20 | 0.00 | 1.00 | 2.20 | 4.00 | 1.80 | 1.20 |
| | **70-74** | 0.00 | 0.00 | 0.07 | 0.00 | 0.13 | 0.13 | 0.40 | 0.33 | 0.73 | 0.73 | 1.73 | 1.53 | 1.53 | 1.20 | 1.87 | 1.47 | 1.60 |
| | **75+** | 0.00 | 0.00 | 0.00 | 0.22 | 0.00 | 0.11 | 0.44 | 0.00 | 0.33 | 0.67 | 0.89 | 1.11 | 2.11 | 1.22 | 1.11 | 3.00 | 5.89 |

**Table S12b**. Original contact matrix of reported contacts for participants not owning or touching animals, consisting of the average number of contact persons recorded per day per survey participant.

| | | Age of contact | | | | | | | | | | | | | | | | |
|---|---|---|---|---|---|---|---|---|---|---|---|---|---|---|---|---|---|---|
| | | **0-2** | **3-6** | **7-9** | **10-14** | **15-19** | **20-24** | **25-29** | **30-34** | **35-39** | **40-44** | **45-49** | **50-54** | **55-59** | **60-64** | **65-69** | **70-74** | **75+** |
| **Age of participant** | **0-2** | 0.64 | 0.31 | 0.00 | 0.06 | 0.03 | 0.03 | 0.92 | 0.97 | 0.81 | 0.08 | 0.03 | 0.17 | 0.47 | 0.81 | 0.67 | 0.14 | 0.17 |
| | **3-6** | 0.95 | 2.65 | 2.17 | 0.50 | 0.08 | 0.42 | 0.95 | 1.82 | 1.15 | 0.48 | 0.35 | 0.52 | 0.92 | 0.52 | 0.22 | 0.05 | 0.05 |
| | **7-9** | 0.04 | 1.92 | 8.88 | 3.36 | 0.12 | 0.04 | 0.40 | 0.72 | 1.24 | 0.36 | 0.16 | 0.08 | 0.24 | 0.52 | 0.92 | 0.20 | 0.08 |
| | **10-14** | 0.00 | 0.47 | 2.68 | 9.49 | 6.49 | 0.23 | 0.36 | 0.77 | 1.23 | 1.00 | 0.72 | 0.32 | 0.17 | 0.23 | 0.57 | 0.11 | 0.21 |
| | **15-19** | 0.00 | 0.00 | 0.06 | 7.16 | 7.50 | 1.84 | 1.66 | 2.00 | 3.69 | 3.19 | 2.53 | 1.25 | 1.31 | 0.31 | 0.31 | 0.19 | 0.69 |
| | **20-24** | 0.11 | 0.00 | 0.00 | 0.04 | 0.18 | 2.61 | 3.14 | 2.46 | 2.93 | 1.79 | 2.50 | 1.89 | 1.82 | 1.04 | 0.25 | 0.07 | 0.32 |
| | **25-29** | 0.04 | 0.21 | 0.12 | 0.08 | 0.21 | 1.69 | 3.13 | 2.73 | 2.71 | 1.79 | 2.02 | 1.33 | 2.17 | 1.29 | 0.67 | 0.13 | 0.27 |
| | **30-34** | 0.19 | 0.70 | 0.30 | 0.19 | 0.21 | 1.45 | 3.42 | 4.70 | 4.25 | 2.21 | 2.08 | 1.70 | 1.91 | 0.94 | 0.47 | 0.26 | 0.23 |
| | **35-39** | 0.09 | 0.12 | 0.25 | 0.36 | 0.20 | 1.05 | 2.25 | 2.38 | 3.66 | 1.82 | 1.59 | 1.16 | 1.20 | 0.62 | 0.25 | 0.16 | 0.11 |
| | **40-44** | 0.00 | 0.05 | 0.11 | 0.39 | 0.39 | 0.55 | 0.87 | 1.26 | 2.74 | 1.92 | 2.50 | 1.05 | 1.03 | 0.58 | 0.71 | 0.50 | 0.68 |
| | **45-49** | 0.00 | 0.05 | 0.12 | 0.32 | 0.25 | 0.85 | 1.17 | 1.53 | 2.07 | 2.03 | 3.20 | 2.07 | 1.64 | 0.88 | 0.73 | 0.75 | 1.10 |
| | **50-54** | 0.03 | 0.08 | 0.05 | 0.13 | 0.29 | 0.74 | 1.66 | 1.47 | 1.97 | 2.66 | 2.55 | 2.47 | 2.11 | 1.58 | 1.05 | 1.03 | 1.32 |
| | **55-59** | 0.02 | 0.12 | 0.02 | 0.07 | 0.02 | 0.47 | 1.02 | 1.19 | 1.44 | 1.30 | 1.63 | 1.74 | 2.40 | 1.19 | 0.56 | 1.30 | 1.37 |
| | **60-64** | 0.02 | 0.02 | 0.00 | 0.10 | 0.06 | 0.19 | 0.46 | 0.58 | 1.19 | 0.88 | 0.40 | 1.06 | 1.88 | 1.83 | 1.38 | 0.54 | 0.77 |
| | **65-69** | 0.02 | 0.10 | 0.03 | 0.05 | 0.05 | 0.10 | 0.33 | 0.43 | 0.93 | 1.02 | 0.93 | 0.93 | 1.64 | 1.79 | 3.59 | 0.84 | 1.14 |
| | **70-74** | 0.01 | 0.01 | 0.17 | 0.10 | 0.07 | 0.11 | 0.23 | 0.16 | 0.47 | 0.54 | 0.69 | 0.64 | 0.47 | 1.41 | 2.01 | 1.57 | 1.54 |
| | **75+** | 0.00 | 0.02 | 0.00 | 0.00 | 0.05 | 0.14 | 0.12 | 0.05 | 0.12 | 0.26 | 0.40 | 0.36 | 0.38 | 1.05 | 1.00 | 0.71 | 3.31 |

**Table S12c**. Original contact matrix (equal 5-year age bands) of reported contacts for participants owning or touching animals, consisting of the average number of contact persons recorded per day per survey participant.

| | | Age of contact | | | | | | | | | | | | | | | |
|---|---|---|---|---|---|---|---|---|---|---|---|---|---|---|---|---|---|
| | | **0-4** | **5-9** | **10-14** | **15-19** | **20-24** | **25-29** | **30-34** | **35-39** | **40-44** | **45-49** | **50-54** | **55-59** | **60-64** | **65-69** | **70-74** | **75+** |
| **Age of participant** | **0-4** | 4.14 | 1.00 | 0.29 | 0.00 | 0.29 | 0.86 | 2.00 | 0.86 | 1.00 | 0.43 | 0.43 | 0.57 | 0.29 | 0.14 | 0.14 | 0.00 |
| | **5-9** | 3.89 | 5.78 | 1.78 | 0.33 | 0.67 | 0.78 | 1.67 | 2.78 | 1.67 | 1.33 | 0.33 | 1.44 | 0.56 | 0.67 | 0.22 | 0.11 |
| | **10-14** | 0.00 | 3.81 | 10.62 | 5.69 | 0.50 | 0.25 | 0.81 | 1.25 | 1.94 | 0.38 | 0.50 | 0.25 | 0.31 | 0.75 | 0.12 | 0.62 |
| | **15-19** | 0.00 | 0.00 | 5.55 | 5.91 | 1.09 | 0.73 | 1.18 | 2.00 | 3.73 | 1.36 | 1.18 | 0.91 | 0.18 | 0.00 | 0.00 | 0.27 |
| | **20-24** | 0.08 | 0.08 | 0.08 | 0.33 | 1.83 | 3.67 | 2.42 | 3.33 | 2.75 | 2.08 | 1.67 | 1.83 | 0.25 | 0.08 | 0.08 | 0.17 |
| | **25-29** | 0.22 | 0.00 | 0.00 | 0.33 | 2.11 | 2.44 | 3.00 | 2.00 | 2.67 | 2.00 | 2.11 | 2.22 | 0.22 | 0.33 | 0.11 | 0.00 |
| | **30-34** | 0.64 | 0.29 | 0.14 | 0.29 | 2.07 | 3.29 | 5.14 | 6.21 | 3.07 | 3.00 | 3.14 | 2.21 | 0.93 | 0.57 | 0.07 | 0.43 |
| | **35-39** | 0.08 | 0.17 | 0.75 | 0.17 | 1.08 | 1.67 | 3.00 | 2.75 | 1.58 | 1.92 | 1.42 | 0.75 | 0.92 | 0.83 | 0.33 | 0.17 |
| | **40-44** | 0.00 | 0.00 | 0.42 | 0.25 | 1.25 | 2.08 | 1.83 | 3.92 | 3.83 | 3.92 | 3.75 | 3.08 | 0.17 | 0.58 | 0.83 | 0.17 |
| | **45-49** | 0.08 | 0.00 | 0.69 | 0.46 | 1.23 | 1.92 | 2.62 | 3.38 | 4.08 | 3.77 | 4.23 | 3.69 | 0.15 | 0.54 | 0.54 | 0.31 |
| | **50-54** | 0.09 | 0.00 | 0.00 | 0.09 | 0.55 | 1.00 | 0.82 | 1.36 | 2.64 | 2.73 | 2.55 | 1.82 | 0.82 | 0.36 | 0.73 | 1.27 |
| | **55-59** | 0.05 | 0.16 | 0.26 | 0.05 | 0.68 | 1.26 | 1.16 | 2.05 | 2.26 | 2.11 | 1.26 | 3.00 | 1.21 | 0.84 | 0.89 | 0.37 |
| | **60-64** | 0.05 | 0.15 | 0.05 | 0.00 | 0.25 | 0.50 | 0.90 | 1.40 | 0.40 | 0.80 | 1.30 | 2.15 | 1.95 | 1.60 | 1.15 | 0.90 |
| | **65-69** | 0.40 | 0.60 | 0.00 | 0.00 | 0.00 | 0.00 | 0.40 | 1.60 | 0.40 | 0.20 | 0.00 | 1.00 | 2.20 | 4.00 | 1.80 | 1.20 |
| | **70-74** | 0.00 | 0.07 | 0.00 | 0.13 | 0.13 | 0.40 | 0.33 | 0.73 | 0.73 | 1.73 | 1.47 | 1.53 | 1.20 | 1.87 | 1.47 | 1.67 |
| | **75+** | 0.00 | 0.00 | 0.22 | 0.00 | 0.22 | 0.44 | 0.00 | 0.56 | 0.56 | 0.78 | 0.89 | 1.89 | 1.33 | 1.00 | 3.11 | 6.11 |

**Table S12d**. Original contact matrix (equal 5-year age bands) of reported contacts for participants not owning or touching animals, consisting of the average number of contact persons recorded per day per survey participant.

| | | Age of contact | | | | | | | | | | | | | | | |
|---|---|---|---|---|---|---|---|---|---|---|---|---|---|---|---|---|---|
| | | **0-4** | **5-9** | **10-14** | **15-19** | **20-24** | **25-29** | **30-34** | **35-39** | **40-44** | **45-49** | **50-54** | **55-59** | **60-64** | **65-69** | **70-74** | **75+** |
| **Age of participant** | **0-4** | 1.75 | 0.00 | 0.05 | 0.07 | 0.20 | 1.03 | 1.31 | 0.93 | 0.27 | 0.15 | 0.42 | 0.78 | 0.64 | 0.46 | 0.10 | 0.12 |
| | **5-9** | 0.74 | 9.57 | 2.43 | 0.07 | 0.17 | 0.48 | 1.19 | 1.21 | 0.36 | 0.24 | 0.07 | 0.36 | 0.64 | 0.64 | 0.14 | 0.07 |
| | **10-14** | 0.00 | 3.17 | 9.49 | 6.53 | 0.23 | 0.36 | 0.77 | 1.21 | 0.98 | 0.72 | 0.32 | 0.17 | 0.23 | 0.55 | 0.11 | 0.21 |
| | **15-19** | 0.00 | 0.06 | 7.16 | 7.50 | 1.84 | 1.66 | 2.00 | 3.69 | 3.19 | 2.53 | 1.25 | 1.31 | 0.31 | 0.31 | 0.19 | 0.69 |
| | **20-24** | 0.11 | 0.00 | 0.04 | 0.18 | 2.71 | 2.93 | 2.54 | 2.93 | 1.79 | 2.46 | 1.89 | 1.86 | 1.07 | 0.25 | 0.07 | 0.32 |
| | **25-29** | 0.21 | 0.15 | 0.10 | 0.21 | 1.69 | 3.15 | 2.73 | 2.71 | 1.79 | 2.02 | 1.33 | 2.17 | 1.27 | 0.65 | 0.13 | 0.27 |
| | **30-34** | 0.57 | 0.62 | 0.19 | 0.21 | 1.45 | 3.42 | 4.74 | 4.25 | 2.19 | 2.08 | 1.72 | 1.91 | 0.94 | 0.47 | 0.23 | 0.23 |
| | **35-39** | 0.12 | 0.36 | 0.34 | 0.21 | 1.09 | 2.18 | 2.45 | 3.66 | 1.73 | 1.55 | 1.12 | 1.21 | 0.70 | 0.27 | 0.16 | 0.11 |
| | **40-44** | 0.05 | 0.11 | 0.39 | 0.45 | 0.50 | 0.87 | 1.18 | 2.71 | 1.89 | 2.50 | 1.11 | 1.00 | 0.61 | 0.74 | 0.53 | 0.71 |
| | **45-49** | 0.03 | 0.14 | 0.32 | 0.25 | 0.85 | 1.17 | 1.51 | 2.07 | 2.07 | 3.20 | 2.03 | 1.66 | 0.85 | 0.75 | 0.76 | 1.10 |
| | **50-54** | 0.08 | 0.08 | 0.13 | 0.29 | 0.74 | 1.66 | 1.47 | 1.97 | 2.66 | 2.53 | 2.47 | 2.13 | 1.58 | 1.05 | 1.03 | 1.32 |
| | **55-59** | 0.12 | 0.05 | 0.07 | 0.02 | 0.47 | 1.00 | 1.21 | 1.44 | 1.33 | 1.65 | 1.72 | 2.40 | 1.16 | 0.56 | 1.30 | 1.37 |
| | **60-64** | 0.04 | 0.00 | 0.10 | 0.06 | 0.19 | 0.48 | 0.52 | 1.17 | 0.94 | 0.44 | 1.04 | 1.88 | 1.88 | 1.35 | 0.54 | 0.73 |
| | **65-69** | 0.05 | 0.09 | 0.05 | 0.05 | 0.10 | 0.33 | 0.40 | 0.90 | 0.98 | 0.95 | 0.93 | 1.59 | 1.83 | 3.62 | 0.90 | 1.17 |
| | **70-74** | 0.01 | 0.20 | 0.10 | 0.07 | 0.11 | 0.23 | 0.17 | 0.49 | 0.57 | 0.64 | 0.60 | 0.47 | 1.43 | 2.03 | 1.54 | 1.56 |
| | **75+** | 0.00 | 0.02 | 0.00 | 0.05 | 0.14 | 0.12 | 0.05 | 0.12 | 0.26 | 0.40 | 0.38 | 0.38 | 0.98 | 1.00 | 0.71 | 3.36 |

**Table S13.** Evaluation of the performance of the two modes of data collection.

| | **Self-reporting** | **Telephone interview** |
|---|---|---|
| Proportion of not reporting on the same day assigned | 22% (85/386) | 17.2% (100/579) |
| Proportion of completing well and very well[a] | 89.6% (285/318) | 95.3% (529/555) |
| Proportion of not including all contacts[b] | 11.1% (43/386) | 10.9% (63/579) |
| Mean number of contacts left out[c] | 1.1 | 0.8 |

[a] corresponds to questions 18, Supplementary Text S1.

[b] corresponds to questions 17, Supplementary Text S1.

[c] corresponds to questions 17, Supplementary Text S1

**Table S14.** Main features of the previous social contact surveys.

| Study | Country or region | Year of study | Mode of data collection | Mode of questionnaire | Prospective or retrospective | Include group | Include animal | Mean number of reported social contacts |
|---|---|---|---|---|---|---|---|---|
| Beutals[1] | Belgium | 2003 | Self-reporting | Online | Both | No | No | 23.5 on weekdays and 19.5 on weekends |
| Mossong[2] | Eight European countries | 2006 | Self-reporting, face-to-face interview | Paper | Not specified | Some included | No | 7.95-19.77 |
| Horby[3] | Vietnam | 2007 | Interview | Paper | Retrospective | No | No | 7.7 |
| McCaw[4] | Australia | 2008 | Self-reporting | Paper and electronic | Prospective | No | No | 20.3-27.5 among 65 adults |
| Danon[5] | UK | 2009 | Self-reporting | Paper and online | Not specified | Yes | No | 26.97 (variance: 5194) |
| Kucharski [6] | Hong Kong | 2009 | Interview | Paper | Not specified | Yes | No | 18.0-18.6 |
| Read[7] | Guangdong | 2010 | Face-to-face interview | Paper | Retrospective | Yes | No | 18.56 |
| Fu[8] | Taiwan | 2010 | Interview | Paper | Retrospective | No | No | 12.54 |
| Johnston [9] | South Africa | 2010 | Self-reporting | Paper | Prospective (encouraged) | No | No | 15.8 |
| Dodd[10] | Zambia and South Africa | 2010/11 | Face-to-face interview | Paper | Retrospective | Yes | No | Close: 4.9 (CI: 4.6-5.2)<br>Casual: 10.4 (CI: 9.3, 11.6) |
| Kifle[11] | Belgium | 2010/11 | Self-reporting | Paper | Prospective | Yes | Yes | 13.5 |
| Ibuka[12] | Japan | 2011 | Self-reporting | Paper and online | Retrospective | No | No | 15.3 |
| Grijalva[13] | Peru | 2011 | Interview | Paper | Retrospective | No | No | 12 |
| Kiti[14] | Kenya | 2011 | Self-reporting | Paper | Prospective (encouraged) | No | No | 17.7 |
| Beraud[15] | France | 2012 | Self-reporting | Paper | Not specified | Yes | No | Individual: 8 (IQR:5-14)<br>Professional: 9 (IQR: 5-17) |
| Kwok[16] | Hong Kong | 2012/13 | Telephone interview | Not clear | Retrospective | Yes | No | 12.5 |
| Melegaro[17] | Zimbabwe | 2013 | Self-reporting | Paper | Prospective (encouraged) | No | No | 10.8 |
| Waroux[18] | Uganda | 2014 | Interview | Paper | Retrospective | Yes | No | 7.2 (range: 1-25) (close contact) |
| Leung[19] | Hong Kong | 2015/16 | Self-reporting | Paper and online | Prospective (encouraged) | No | No | 8.1 |
| Ajelli[20] | Russia | 2016 | Self-reporting | Paper | Not specified | No | No | 12.2 |
| Klepac[21] | UK | 2017 | Self-reporting | Online | Prospective (encouraged) | No | No | - |

## References


1 Beutels, P., Shkedy, Z., Aerts, M. & Van Damme, P. Social mixing patterns for transmission models of close contact infections: exploring self-evaluation and diary-based data collection through a web-based interface. *Epidemiol Infect* **134**, 1158-1166, doi:10.1017/S0950268806006418 (2006).

2 Mossong, J. *et al.* Social contacts and mixing patterns relevant to the spread of infectious diseases. *Plos Medicine* **5**, e74, doi:10.1371/journal.pmed.0050074 (2008).

3 Horby, P. *et al.* Social Contact Patterns in Vietnam and Implications for the Control of Infectious Diseases. *PLOS ONE* **6**, e16965, doi:10.1371/journal.pone.0016965 (2011).

4 McCaw, J. M. *et al.* Comparison of three methods for ascertainment of contact information relevant to respiratory pathogen transmission in encounter networks. *BMC Infect Dis* **10**, 166, doi:10.1186/1471-2334-10-166 (2010).

5 Danon, L., House, T. A., Read, J. M. & Keeling, M. J. Social encounter networks: collective properties and disease transmission. *Journal of the Royal Society, Interface* **9**, 2826-2833, doi:10.1098/rsif.2012.0357 (2012).

6 J. Kucharski, A. *et al.* The Contribution of Social Behaviour to the Transmission of Influenza A in a Human Population. *Plos Pathogens* **10**, doi:10.1371/journal.ppat.1004206 (2014).

7 Read, J. M. *et al.* Social mixing patterns in rural and urban areas of southern China. *Proceedings of the Royal Society B Biological Sciences* **281**, 20140268, doi:10.1098/rspb.2014.0268 (2014).

8 Fu, Y. C., Wang, D. W. & Chuang, J. H. Representative contact diaries for modeling the spread of infectious diseases in Taiwan. *PLoS One* **7**, e45113, doi:10.1371/journal.pone.0045113 (2012).

9 Johnstone-Robertson, S. P. *et al.* Social mixing patterns within a South African township community: implications for respiratory disease transmission and control. *Am J Epidemiol* **174**, 1246-1255, doi:10.1093/aje/kwr251 (2011).

10 Dodd, P. J. *et al.* Age- and Sex-Specific Social Contact Patterns and Incidence of Mycobacterium tuberculosis Infection. *Am J Epidemiol* **183**, 156-166, doi:10.1093/aje/kwv160 (2016).

11 Wasihun, Y. *et al.* Animal Ownership and Touching Enrich the Context of Social Contacts Relevant to the Spread of Human Infectious Diseases (vol 10, e0133461, 2015). *PLOS ONE* **11** (2016).

12 Ibuka, Y. *et al.* Social contacts, vaccination decisions and influenza in Japan. *Journal of epidemiology and community health* **70**, 162-167, doi:10.1136/jech-2015-205777 (2016).

13 Grijalva, C. G. *et al.* A household-based study of contact networks relevant for the spread of infectious diseases in the highlands of Peru. *PLoS One* **10**, e0118457, doi:10.1371/journal.pone.0118457 (2015).

14 Kiti, M. C. *et al.* Quantifying Age-Related Rates of Social Contact Using Diaries in a Rural Coastal Population of Kenya. *PLOS ONE* **9**, e104786, doi:10.1371/journal.pone.0104786 (2014).

15 Beraud, G. *et al.* The French Connection: The First Large Population-Based Contact

Survey in France Relevant for the Spread of Infectious Diseases. *PLoS One* **10**, e0133203, doi:10.1371/journal.pone.0133203 (2015).

16 Kwok, K. O., Cowling, B., Wei, V., Riley, S. & Read, J. M. Temporal variation of human encounters and the number of locations in which they occur: a longitudinal study of Hong Kong residents. *Journal of the Royal Society, Interface* **15**, doi:10.1098/rsif.2017.0838 (2018).

17 Melegaro, A. *et al.* Social Contact Structures and Time Use Patterns in the Manicaland Province of Zimbabwe. *PLOS ONE* **12**, e0170459, doi:10.1371/journal.pone.0170459 (2017).

18 le Polain de Waroux, O. *et al.* Characteristics of human encounters and social mixing patterns relevant to infectious diseases spread by close contact: a survey in Southwest Uganda. *BMC Infectious Diseases* **18**, 172, doi:10.1186/s12879-018-3073-1 (2018).

19 Leung, K., Jit, M., Lau, E. H. Y. & Wu, J. T. Social contact patterns relevant to the spread of respiratory infectious diseases in Hong Kong. *Sci Rep* **7**, 7974, doi:10.1038/s41598-017-08241-1 (2017).

20 Ajelli, M. & Litvinova, M. Estimating contact patterns relevant to the spread of infectious diseases in Russia. *J Theor Biol* **419**, 1-7, doi:10.1016/j.jtbi.2017.01.041 (2017).

21 Klepac, P., Kissler, S. & Gog, J. Contagion! The BBC Four Pandemic – The model behind the documentary. *Epidemics*, doi:10.1016/j.epidem.2018.03.003 (2018).
