# Supplementary material for: Patterns of human social contact and contact with animals in Shanghai, China
Source: Sci Rep. 2019 Oct 22;9:15141. doi: 10.1038/s41598-019-51609-8 (PMC6805924; doi:10.1038/s41598-019-51609-8)
Supplement: Supplementary file 3 — Supplementary text [file 41598_2019_51609_MOESM3_ESM.pdf]

Patterns of human social contact and contact with animals in Shanghai, China

Juanjuan Zhang[1], Petra Klepac[2], Jonathan M. Read[3], Alicia Rosello[2], Xiling Wang[1], Shengjie Lai[1,4,5], Meng Li[1], Yujian Song[1], Qingzhen Wei[1], Hao Jiang[1], Juan Yang[1], Henry Lynn[1], Stefan Flasche[2], Mark Jit[2,6,7], Hongjie Yu[1*]

[1]*School of Public Health, Fudan University, Key Laboratory of Public Health Safety, Ministry of Education, Shanghai, China*

[2]*Department of Infectious Disease Epidemiology, Faculty of Epidemiology and Public Health, London School of Hygiene and Tropical Medicine, London, UK.*

[3]*Centre for Health Informatics, Computation and Statistics, Lancaster Medical School, Lancaster University, Lancashire, UK.*

[4]*WorldPop, School of Geography and Environmental Science, University of Southampton, Southampton, UK*

[5]*Flowminder Foundation, Stockholm, Sweden*

[6]*Modelling and Economics Unit, Public Health England, London, UK*

[7]*School of Public Health, University of Hong Kong, Hong Kong, China*

*Corresponding author:

Professor Hongjie Yu

School of Public Health

Fudan University

Email: yhj@fudan.edu.cn

Supplementary Text S1: Sampling design and study population

Sample design

We first sampled three central urban districts, then sampled a total of ten subdistricts within those three central urban districts, and from each of those ten subdistricts we sampled four neighborhoods (the smallest administrative unit in China, with a mean population of 3800 people per neighborhood sampled), using multi-stage stratified probability proportional to population size (PPS). At the district level, three districts were sampled from the total seven central urban districts of Shanghai by PPS (Huangpu, Xuhui, and Changning). At the subdistrict level, ten subdistricts within those three central urban districts were sampled by PPS, where the number of subdistricts needed per district was determined by proportionate stratification (four from Xuhui, three from Huangpu, and three from Changning). At the household level, 25 households per neighborhood were selected with the help of neighborhood committee cadres. The selection of households was encouraged to be broadly representative of the population of the neighborhood in terms of geographical spread. One person per household was invited to participate in our study until we met our predefined target sizes by age and gender (described below, table A). A pilot survey was conducted between December 2017 and January 2018, with two of the selected neighborhoods as study sites.

Sample size allocation at each stage had to do with the sample size calculation, which was described below.

Sample size calculation

The sample size was calculated based on the key variable - “the number of daily contacts”. We assumed that intra-class correlation among individuals only existed at neighborhood level, not at subdistrict and not even at district. Comparing our complex sample with an unrestricted sample design, we accounted for clustering at neighborhood level, by multiplying the effective sample size by the design effect. Hence the sample size calculation was as follows:

$$n = \frac{{\mu_\alpha}^2\sigma^2}{\delta^2} * D^2$$

Where design effect (D^2) denoted the ratio of the variance of a statistic with a complex sample design to the variance of that statistic with a simple random sample or an unrestricted sample of the same size. The design effect can be approximated by

$$D^2 = 1 + (b-1)\bar{\rho}$$

where b is the number of sampled households per neighborhood, and $\bar{\rho}$ is the average intra-class correlation across neighborhoods, denoting a within-cluster measure of homogeneity. The optimum number of households to be selected for each neighborhood depended on the data-collection cost structure and the degree of homogeneity. Assuming a linear cost model for the overall cost related to the sampling of resident groups and households given by

$$C = aC_1 + abC_2$$

Where $C_1$and C_2 are respectively the cost of an additional neighborhood and the cost of an additional household; and *a* and *b* denote respectively the number of selected neighborhoods and households per resident group. Under this cost model, the optimum choice for *b* that minimizes the variance of the sample mean is approximately given by

$$b_{opt} = \sqrt{\frac{C_1}{C_2}\frac{(1-\rho)}{\rho}}$$

To sum up, the sample size calculation formula can be integrated as below:

$$n = \frac{{\mu_\alpha}^2\sigma^2}{\delta^2} * (1 + \left(\sqrt{\frac{C_1}{C_2}\frac{(1-\rho)}{\rho}} - 1\right) * \bar{\rho})$$

Assumptions for the sample size calculation:

- 95% confidence, alpha equals 0.05.
- Sigma equals 6.78, referring to a published social contact survey in Hong Kong[1], as Shanghai may have similar characteristics of high population density and connectivity to Hong Kong.
- Delta equals 0.6, a specified level of precision.

- C_1/C_2 equals 25, based on our budget.
- ρ equals 0.03, assuming equal ICC across clusters ($\bar{\rho} = \rho$). There was no published data about ICC to be used in our social contact study, so we estimated it according to our pilot study results.

Based on the above assumptions, a total sample size of **894** was required. Assuming that **90%** of the participants would eventually return a completed survey, we need recruit approximately **1,000** participants for the study.

In this case, as b equaled 25, 25 households were needed per neighborhood, and thus forty neighborhoods were needed in total. Four neighborhoods were sampled per subdistrict, thus a total of ten subdistricts were required for the three central districts. According to proportionate stratified PPS, we sampled four subdistricts in Xuhui, three in Changning and three for Huangpu.

Study population

Eight age groups were defined as 0-9, 10-19, 20-29, 30-39, 40-49, 50-59, 60-69, 70 years and above. Considering the final sample size of 1000, we aimed for each age group to contain 125 individuals. In addition, children and adolescents younger than 20 years old were oversampled because they were considered to be the main driver for transmission of many infectious respiratory diseases (as in [2]). Table A showed the age distribution of the population of Shanghai (central urban districts) in 2017 (in which the total population size is about 7.3 million) compared with that in our sample. Figure A described which age groups were oversampled.

Table A. Age distribution of Shanghai (central urban districts) population vs. our sample

**Age group**	**0-9**	**10-19**	**20-29**	**30-39**	**40-49**	**50-59**	**60-69**	**70+**
**Shanghai (%)**	5.2	5.6	19.7	13.6	12.3	19.8	11.0	12.8
**Sample (%)**	12.5	12.5	12.5	12.5	12.5	12.5	12.5	12.5

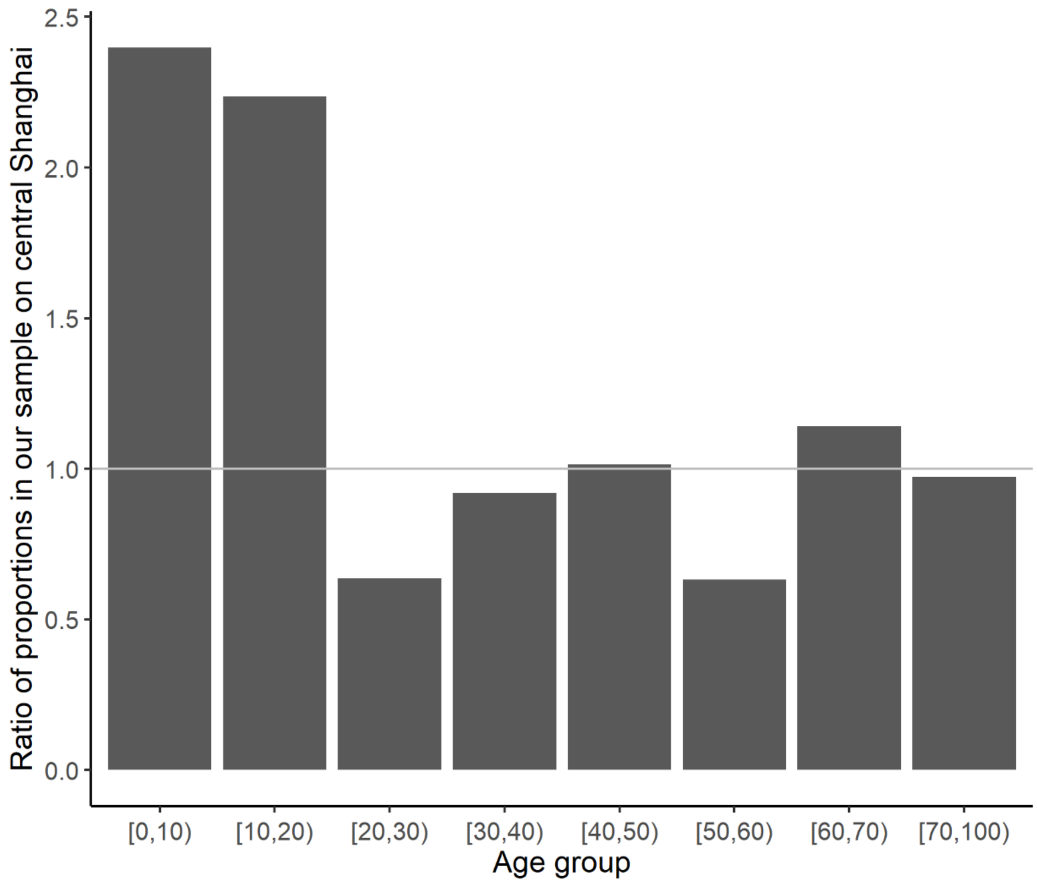

Figure A. The ratio of the proportions of age groups in our sample on the central urban population of Shanghai. The age groups with a ratio greater than one have been oversampled, otherwise, under sampled.

More practically, inclusion criteria for the study were 1) a Chinese person who had been living in Shanghai for longer than two weeks and did not intend to move from Shanghai in the following two weeks, 2) being able to move independently and understand clearly the content of our questionnaire, and 3) giving written individual informed consent (from a parent/guardian if participant was below 18 years of age).

Supplementary Text S2: Data collection

Recall bias. In order to explore recall bias at the end of H-H section, we asked questions to assess participants' memory such as "How well do you recall your contacts today?". There were five options for selection, including "very well", "well", "moderately well", "not well" and "poorly". Additionally, participants were required to estimate approximately how many people they might have accidentally forgotten to include (Supplementary Text S12; questions 17-18.). This information is important to evaluate the quality of data collection.

Tips on filling in the questionnaire. If the same person was encountered several times during the assigned day, participants were instructed to only record him/her once, and to record the total time participants spent with that person over the entire day. Reporting a group contact involved reporting the total (approximate) size of the group and the age range of the majority in the group, rather than reporting each of these contacts individually. Participants could report only one group contact per survey (see Supplementary Text S12; questions 13-15.).

Who completed the questionnaire depended on the participant's age, 1) parental-proxy completion for 0 to 10, 2) completion by participant for 11 to 17 subject to parental informed consent, and 3) completion by participant for 18 or above.

Supplementary Text S3: Estimating of the human-human contact duration, the number of contact settings, and the age of contacts

The duration of individual contact was reported in one of four categories (i.e. 0-4 mins, 5-14 mins, 15-59 mins, 1-4 hours, over 4 hours), so we defined exact duration as the midpoint of the corresponding category right censored at 4 hours. To estimate each group contact duration, we fitted a Gaussian kernel function to the observed individual contact duration crossed by different age groups of participants and contacts (the age group was divided into 0-35m, 3-6y, 7-19y, 20-39y, 40-59y, 60-75y, 76y and over, according to different stages of children, adults and the elderly). Then, drawing randomly from the model referring to the age group of the participant and of the contact, we repeated this process 200 times to permit estimation of uncertainty (Supplementary Fig. S5 B).

The number of contact settings was estimated as the total number of different social setting in which contact was reported, where the maximum was defined by the settings categorization: home, school, work, transport, other public places and others (i.e. 6).

For the age of individual contacts, participants were asked to give the age range of their contacts or the exact age only if known or easily estimable; therefore, the exact age was unavailable for a proportion of contacts. To solve the issue, the exact age of contacts given as a range was assumed to be the mid-point of the given range. In order to determine the age of contacts for which there was no age information (2% of the age of contacts were completely missing), the age was sampled from all the contacts of participants of the same age group.

For the age of group contacts, participants did not record the exact age of each

contact, so we modelled the distribution of individual contact age using Gaussian kernel function, stratified by different age groups of participants (0-35m, 3-6y, 7-19y, 20-39y, 40-59y, 60-75y, 76y and over) (rather than modelling the number of contacts made with each age group). Then we drew randomly from the model referring to the age group of participants and the age group of the group contacts selected in the questionnaire and repeated this process 200 times to estimate uncertainty (Supplementary Fig. S5 A).

Supplementary Text S4: Regression models

In the models of the H-H contacts, specifically, log(1+n_{ij}), log(1+k_{ij}), log(1+d_{ij}), and log(1+s_{ij}) were defined as the response variables with negative binomial distributions, where n_{ij} is the number of total contacts reported by participant j from district i, k_{ij} is the number of individual contacts, d_{ij} is the total duration of contacts, and s_{ij} is the number of contact settings; the probability of reporting group contact was assumed with quasibinomial distribution.

In the models of the H-A contacts, response variables were assumed with quasibinomial distributions. The number of social contacts, as well as all the covariates (demographic and socioeconomic, travel, temporal, and other survey-related factors) were included as possible predictors of animal contact, whereas only the number of social contacts and some covariates (demographic and socioeconomic, travel, and other survey-related factors) were included as possible predictors of animal ownership. Here we used original data instead of propensity score matched data, as we found no significant difference between two modes of data collection after the propensity score matching.

In order to select which explanatory variables to include in the final model, we first used univariate regression for candidate variable selection (alpha equals 0.1, generalized linear models were used with the same link functions as the multivariate models mentioned above), then used multivariate regressions, and finalized as bidirectional stepwise model selection based on Akaike's Information Criterion (AIC). Models were based on age-weighted methods. The sampling weights were calculated based on the age distribution of Shanghai (central urban districts) census in 2017. The distribution of household sizes was not considered because we could not obtain the census statistics of household size stratified by age.

Supplementary Text S5: Human-human contact matrix

We defined the original matrix $\boldsymbol{C}=(C_{ij})$ where C_{ij} was the number of encounters between participants of age group i and contacts of estimated age j. The total number of participants in each age class was N_i. The original contact matrix was normalized by these to give the mean contact matrix $\boldsymbol{M}=(M_{ij})$ where $M_{ij}= C_{ij} / N_i$. Conceivably, contacts should be symmetric: the total number of contacts made by members of one age group with those of another should be the same as vice versa. Mathematically, $M_{ij}N_i=M_{ji}N_j$, the resulting symmetric matrix had $S_{ij} = \frac{M_{ij}N_i+M_{ji}N_j}{2N_i}$. Finally, matrices were weighted based on age, where the sampling weights were calculated based on the age distribution of Shanghai (central urban districts) census in 2017, so our final contact matrix is $\boldsymbol{W}=(W_{ij})= S_{ij}\, n_i/n_j$, where n_i and n_j are respective proportions in age groups i and j of the whole population.

In order to assess the degree of age assortativity within the obtained human contact matrices, we calculated the q indices and the bootstrapped 95% confidence intervals[3], a measurement representing departures from proportionate mixing, ranging from zero (proportionate) to one (fully assortative). Compared to the Q index, the values of the minor eigenvalues $(\widehat{\lambda_2}\ldots\widehat{\lambda_M})$ relative to the dominant eigenvalue $(\widehat{\lambda_1})$ of the contact matrix provides a more natural description of the spread of infection between age groups. In particular, $q = \widehat{\lambda_2}/\widehat{\lambda_1}$ is an alternative measure of assortative mixing, effectively weighting mixing between similar classes.

Supplementary Text S6: Bivariate smoothing of human-human contact matrix

We used bivariate smoothing approach to estimate the m_{ij} elements of the social contact matrix. The basis was a tensor-product spline ensuring flexibility when modeling the average number of contacts as a function of the responder's and contact's age over 1-year band. The average number of contacts between person aged i with person aged j is modeled using a two-dimensional continuous function applied to the age of participants and respondents, via a generalized additive model (GAM), which was performed in R with the *gam* function from the *mgcv* package[4,5]. We used smooth of both age variables from tensor products, ensuring flexibility.

To allow for over dispersion in the number of contacts, we assumed that they are distributed according to a negative binomial distribution with mean m_{ij}, dispersion parameter k, and variance $m_{ij} + m_{ij}^2/k$. We used the basis dimension K=8 in order to be large enough to fit the data well, but small enough to keep the fitting procedure computationally efficient[4,5]. Then, we chose the thin plate regression splines in order to avoid the selection of knots, and a log link function for the GAM. Finally, we accounted for the reciprocal nature of data and predicted the expected number of contacts at the population level.

Supplementary Text S7: Hypothesis testing for mode of data collection

We tested the null hypothesis that telephone interviewed respondents would report more contacts by computing the difference between human contact matrices from self-reporting and telephone interviewed respondents. Confidence intervals were obtained through bootstrapping, with replacement, a total of 1000 iterations.

Supplementary Text S8: Some results for individual contacts and group contacts

Distribution of individual and group contacts

The distribution of the number of individual contacts was right-skewed with a median of 5 contacts. The number of individual contacts increased with participants' age except for people aged over 75 years. 30.8% respondents reported group contacts, of which the distribution of the number of group contacts was right-skewed with a median of 30. Participants age was significantly associated with the probability of reporting group contact, with a peak for 7-19 years (Supplementary Fig. S6, Supplementary Table S3).

Regression results of individual and group contacts

We found a significant incremental linear association between the number of individual contacts and participant age. We found no significant association between the number of individual contacts and modes of data collection, but household size above 4 was associated with a greater number of individual contacts. The probability of reporting group contacts was significantly associated nonlinearly with participant age, with a similar relationship of contacts and participant age to the number of total contacts. Telephone interview was associated with a significantly greater probability of reporting group contacts than self-reporting. Workdays and higher population density were associated with a significantly greater probability of reporting contacts (Supplementary Fig. S14 and Supplementary Table S9).

Contact matrix for individual and group contacts

For the individual contact matrix, we found stronger diagonals and clearer parallel secondary diagonals than that for total contacts, which was most pronounced in those aged above75 years. For group contacts, we found strong diagonals for participants aged below 20 years, more age-mixed patterns among 20-60 years, and dispersed pattern for those above 60-year-old. Compared to self-reporting, telephone interview

had a more similar pattern with the overall matrix (Supplementary Fig. S16).

Supplementary Text S9: More results for human-animal contact patterns

The distribution of the number of animal contacts was right-skewed with a median of one animal. Children aged 3-6 years, especially females, had the highest number of animal contacts (Fig. 2 B1-B2). The distribution of the animal contact duration was also right-skewed with a median of 0.62 hours. In those aged 20-74, animal contact duration was much longer in females than in males, but this was reversed in other age groups (Fig. 2 C1-C2). Most frequently, participants had contact with one kind of animal, and amongst the animal species encountered, dogs still dominated (45.1%) (Fig. 2 D1-D2). Most of the animal contacts occurred in the home setting (81.6%). Adults aged 60-75, especially females, more frequently had animal contact outside of the home (Fig. 2 E1-E3).

Supplementary Text S10: The evaluation of the quality of data collection

78% participants who chose self-reporting filled out the questionnaire on the day that was assigned to them, while 82.8% participants who chose telephone interview had completed the interview on the assigned day. The proportion of rating their own recall performance as "well" or "very well" was 89.6% and 95.3% respectively for self-reporting and telephone interview. For self-reporting, 11.1% participants reported not including all contacts, and the mean number of contacts that had been left out was 1.1. For telephone interview, 10.9% participants reported not including all contacts, and the mean number of contacts left out was 0.8. To sum up, telephone interview participants rated the quality of their responses across a number of dimensions higher than self-reporting participants, although both of the different modes of data collection had a good quality of data collection (Supplementary Table S13).

Supplementary Text S11: Initial phase of an Epidemic simulations

We explored the age-specific incidence of infection during the initial phase of an epidemic of an emerging infectious disease agent that spreads in a completely susceptible population. We focused on the generic features of epidemic spread along the transmission route that was specified by physical and nonphysical contacts.

It is worth noting that the age distribution of cases during the early phase of the epidemic depends neither on the choice of the duration of the infectivity period, nor on the considered value of the basic reproductive number R_0. This means that, although the attack rate, the timing, and the severity associated with an infection are generally disease-specific, the impact of mixing patterns on the age distribution of cases within a susceptible population are mainly driven by the type of contacts relevant for infection transmission and the socio-demographic structure of the considered population. Here we built a Susceptible-Infected-Recovered (SIR) model based on the age distribution of the population of Shanghai central urban areas, assuming an age-independent transmission rate per contact if 0.1, and the duration of infectiousness as 14 days.

As the POLYMOD study suggested, in practice, the distribution of new cases is stable after five generations; that is, the distribution no longer depends on the precise age of the initial case. Hence, the incidence of new infections per age band at step 5 was obtained by dividing the expected number of new cases per age class by the number of individuals in each age class. To facilitate comparison among countries, we normalized the distribution of incidence over age classes such that the age-specific incidences summed to one.

Estimates obtained using the contact data from this study were compared with those of Hong Kong[1], POLYMOD[2], France[6], and Peru[7], using a similar approach to compute the mixing matrix. Additionally, estimates from assumed random mixing were also included. The contact matrices from different studies are displayed in Supplementary Fig. S23, and the observed relative incidence was showed in Supplementary Fig. S24.

Supplementary Text S12: Sensitivity analysis

We did notice different results using data collected by different interviewers, but we don't think it is entirely due to having different interviewers. The reason is that we did not randomly assign interviewers to call the people, but non-randomly assigned one interviewer to a sampled neighborhood including 25 participants. Thus, the different results derived from different interviewers might be confounded by neighborhood-level factors, such as age structure and population density of the neighborhood.

None the less, we did perform a sensitivity analysis using the data derived from telephone interview with four interviewers involved (i.e. "Interviewer A", "Interviewer B", "Interviewer C", "Interviewer D") (see below table A, table B, and figure A). Negative binomial regression models built to compare the number of contacts recorded by different interviewers showed a significant difference between Interviewer D and Interviewer C. We then removed the data recorded by Interviewer D and re-calculated means and 95% confidence intervals of three key measures (i.e. number of contacts, contact duration and number of contact settings) as results of sensitivity analysis. We found all of the confidence intervals in sensitivity analysis covered the means of our primary results, which suggests different interviewers made little influence on the primary estimates here.

Table A. Mean and median number of contacts recorded by different telephone interviewers

Interviewer	N (%)	Mean contacts	Median contact (IQR)
**Overall**	579	19.3	10 (25.5)
**Interviewer A**	105 (18.1)	17.9	10 (22)
**Interviewer B**	247 (42.7)	19.7	10 (29)
**Interviewer C**	157 (27.1)	21.7	14 (23)
**Interviewer D**	70 (12.1)	14.0	5 (21)

Table B. Comparison of primary results and sensitivity analysis results

	N	Mean contacts (95% CI)	Mean contact duration (95% CI)	Mean contact settings (95% CI)
**Primary**	579	19.3 (17.6, 20.9)	34.9 (31.7, 38.1)	2.0 (2.0, 2.1)
**Sensitive**	509	20.0 (18.2, 21.8)	35.9 (32.4, 39.3)	2.1 (2.0, 2.1)

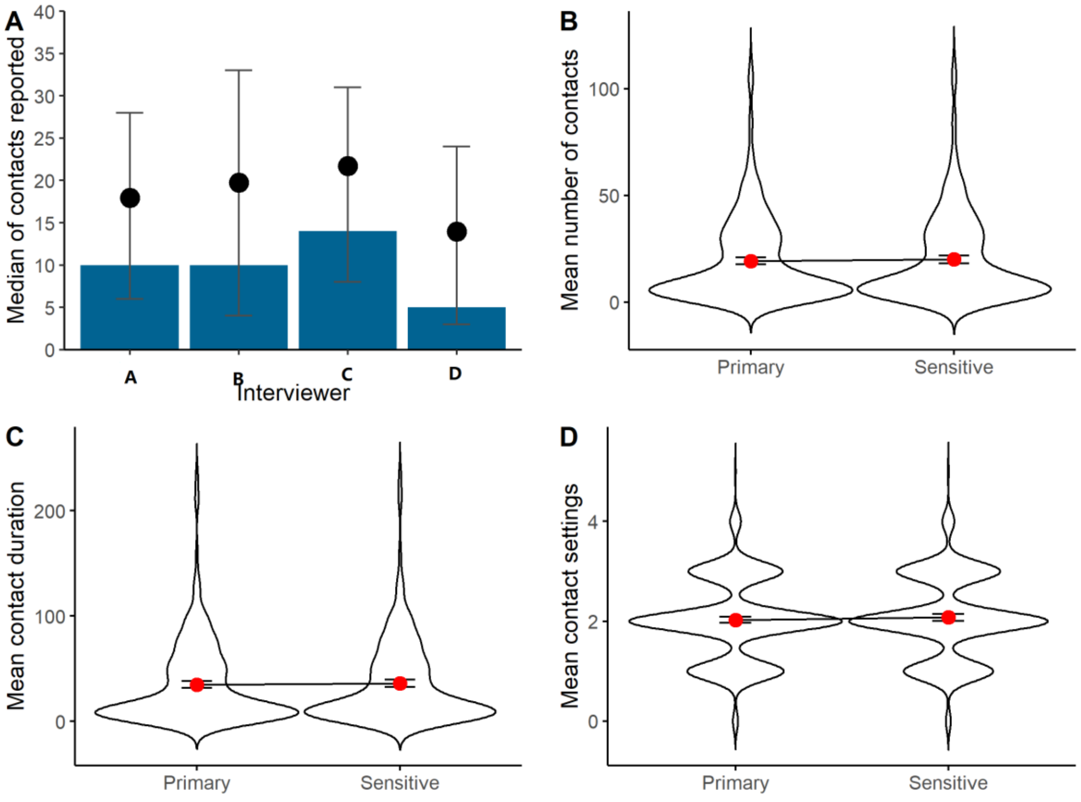

Figure A. Comparison of primary and sensitivity analysis results considering heterogeneity in results from different interviewers. (A) Distribution of number of contacts recorded by different telephone interviewers. The error bars correspond to 25% and 75% quantiles, and the solid points correspond to the means. (B-D) Violin plots of mean contacts, mean contact duration, and mean contact settings in primary and sensitive results. Red solid points and error bars correspond to means and 95% CI.

References

1 Leung, K., Jit, M., Lau, E. H. Y. & Wu, J. T. Social contact patterns relevant to the spread of respiratory infectious diseases in Hong Kong. *Sci Rep* **7**, 7974, doi:10.1038/s41598-017-08241-1 (2017).

2 Mossong, J. *et al.* Social contacts and mixing patterns relevant to the spread of infectious diseases. *Plos Medicine* **5**, e74, doi:10.1371/journal.pmed.0050074 (2008).

3 Keeling, M. J. & Rohani, P. in *Modeling infectious diseases in humans and animals* Ch. 3, 69 (Princeton University Press, 2011).

4 Goeyvaerts, N. *et al. Estimating infectious disease parameters from data on social contacts and serological status*. Vol. 59 (2010).

5 Wood, S. N. *Generalized Additive Models, an Introduction with R*. (CRC Press, 2006).

6 Beraud, G. *et al.* The French Connection: The First Large Population-Based Contact Survey in France Relevant for the Spread of Infectious Diseases. *PLoS One* **10**, e0133203, doi:10.1371/journal.pone.0133203 (2015).

7 Grijalva, C. G. *et al.* A household-based study of contact networks relevant for the spread of infectious diseases in the highlands of Peru. *PLoS One* **10**, e0118457, doi:10.1371/journal.pone.0118457 (2015).

Supplementary Text S13

INTRODUCTION

Participant ID:

Assigned date: _____ _____ (DD) / _____ _____ (MM)/ _____ __________ _____ (YYYY)

Thank you for participating in our study "Social contact and travel survey in Shanghai City".

The questionnaire consists of 3 sections:

- Your demographic data, general household and travel information
- Human-to-human contacts you have made during the assigned day
- Human-to-animal contacts you have made during the assigned day

I give permission to the investigators to contact me within 12 months for potential follow-up for this study. Please provide your information below.

Signature: ____

Phone: ____

Address: ____

PART 1: DEMOGRAPHIC DATA
(Please tick the appropriate option☑)

1. **Please provide your age OR age range:**

   Age: ________ Years

   Age range (Please select your age range below):

   ☐ 0-4 ☐ 5-9 ☐ 10-14 ☐ 15-19 ☐ 20-24 ☐ 25-29
   ☐ 30-34 ☐ 35-39 ☐ 40-44 ☐ 45-49 ☐ 50-54 ☐ 55-59
   ☐ 60-64 ☐ 65-69 ☐ 70-74 ☐ >=75 ☐ Unwilling to answer

2. **Sex:**

   ☐ Male ☐ Female ☐ Others ☐ Unwilling to answer

3. **Please select your working or school status:**

   ☐ Full-time ☐ Part-time ☐ No job/pre-school ☐ Unwilling to answer

4. **Please select your working role:**

   ☐ Pre-school ☐ Students ☐ Retired ☐ Unemployed
   ☐ Service workers ☐ Healthcare workers ☐ Skilled workers ☐ Professionals
   ☐ Home makers ☐ Businessman selling live poultry or meat ☐ Others: ________
   ☐ Don't know ☐ Unwilling to answer

5. **Highest level of education attained (completed):**

   ☐ None ☐ Primary school ☐ Middle school ☐ High school
   ☐ College and above ☐ Others: _________ ☐ Don't know ☐ Unwilling to answer

6. **Please select to provide your individual OR household monthly income (RMB):**

   ☐ Individual income ☐ Household income

   Please provide the range of monthly income:

   ☐ <1,000 ☐ 1,001-2,000 ☐ 2,001-4,000 ☐ 4,001-6,000 ☐ 6,001-8,000
   ☐ 8,001-10,000 ☐ 10,001-15,000 ☐ 15,001-20,000 ☐ 20,001-30,000 ☐ >30,000
   ☐ None ☐ Don't know ☐ Unwilling to answer

7. **How long have you lived here? (year)**

   ☐ < 1 ☐ 1-5 ☐ 5-10 ☐ >10 ☐ Don't know ☐ Unwilling to answer

8. **How many people do you generally live with (sleep in the place more than 4 nights per week)?**

   Number of persons: ☐ persons

   Type: ☐ Household ☐ Shared apartment
   ☐ Student residential hall ☐ Other :

9. What are the age, sex and relationship to you for people who you reported in Question 8? Please list the 10 most intimate people if there is not enough space.

Persons who live with you

1 **Age:** ______ years or ______ - ______ years **Sex:** ☐ Male ☐ Female
Relationship: ☐ Spouse/partner ☐ Parent ☐ Child ☐ Sibling ☐ Grandparent ☐ Grandchild
☐ Classmate ☐ Colleague ☐ Boy/girl friend ☐ Flat-share ☐ Other:_____ ☐ Unwilling to answer

2 **Age:** ______ years or ______ - ______ years **Sex:** ☐ Male ☐ Female
Relationship: ☐ Spouse/partner ☐ Parent ☐ Child ☐ Sibling ☐ Grandparent ☐ Grandchild
☐ Classmate ☐ Colleague ☐ Boy/girl friend ☐ Flat-share ☐ Other:_____ ☐ Unwilling to answer

3 **Age:** ______ years or ______ - ______ years **Sex:** ☐ Male ☐ Female
Relationship: ☐ Spouse/partner ☐ Parent ☐ Child ☐ Sibling ☐ Grandparent ☐ Grandchild
☐ Classmate ☐ Colleague ☐ Boy/girl friend ☐ Flat-share ☐ Other:_____ ☐ Unwilling to answer

4 **Age:** ______ years or ______ - ______ years **Sex:** ☐ Male ☐ Female
Relationship: ☐ Spouse/partner ☐ Parent ☐ Child ☐ Sibling ☐ Grandparent ☐ Grandchild
☐ Classmate ☐ Colleague ☐ Boy/girl friend ☐ Flat-share ☐ Other:_____ ☐ Unwilling to answer

5 **Age:** ______ years or ______ - ______ years **Sex:** ☐ Male ☐ Female
Relationship: ☐ Spouse/partner ☐ Parent ☐ Child ☐ Sibling ☐ Grandparent ☐ Grandchild
☐ Classmate ☐ Colleague ☐ Boy/girl friend ☐ Flat-share ☐ Other:_____ ☐ Unwilling to answer

6 **Age:** ______ years or ______ - ______ years **Sex:** ☐ Male ☐ Female
Relationship: ☐ Spouse/partner ☐ Parent ☐ Child ☐ Sibling ☐ Grandparent ☐ Grandchild
☐ Classmate ☐ Colleague ☐ Boy/girl friend ☐ Flat-share ☐ Other:_____ ☐ Unwilling to answer

7 **Age:** ______ years or ______ - ______ years **Sex:** ☐ Male ☐ Female
Relationship: ☐ Spouse/partner ☐ Parent ☐ Child ☐ Sibling ☐ Grandparent ☐ Grandchild
☐ Classmate ☐ Colleague ☐ Boy/girl friend ☐ Flat-share ☐ Other:_____ ☐ Unwilling to answer

8 **Age:** ______ years or ______ - ______ years **Sex:** ☐ Male ☐ Female
Relationship: ☐ Spouse/partner ☐ Parent ☐ Child ☐ Sibling ☐ Grandparent ☐ Grandchild
☐ Classmate ☐ Colleague ☐ Boy/girl friend ☐ Flat-share ☐ Other:_____ ☐ Unwilling to answer

9 **Age:** ______ years or ______ - ______ years **Sex:** ☐ Male ☐ Female
Relationship: ☐ Spouse/partner ☐ Parent ☐ Child ☐ Sibling ☐ Grandparent ☐ Grandchild
☐ Classmate ☐ Colleague ☐ Boy/girl friend ☐ Flat-share ☐ Other:_____ ☐ Unwilling to answer

10 **Age:** ______ years or ______ - ______ years **Sex:** ☐ Male ☐ Female
Relationship: ☐ Spouse/partner ☐ Parent ☐ Child ☐ Sibling ☐ Grandparent ☐ Grandchild
☐ Classmate ☐ Colleague ☐ Boy/girl friend ☐ Flat-share ☐ Other:_____ ☐ Unwilling to answer

10. Where and how often do you usually have a travel?

**Where**	**How often (fill in the No. on the right)**	
☐ Outside Village	____	1. Most days of the week
☐ Outside Town	____	2. At least once a week but not most days
☐ Outside District/County	____	3. At least once a month but not each week
☐ Outside City/Province	____	4. Less than monthly, but not never
☐ Abroad	____	5. Never
☐ Don't know		6. Don't know

11. Where and how often do you and your housemate usually have a travel together (at least two persons)?

**Where**	**How often (fill in the No. on the right)**	
☐ Outside Village	____	1. Most days of the week
☐ Outside Town	____	2. At least once a week but not most days
☐ Outside District/County	____	3. At least once a month but not each week
☐ Outside City/Province	____	4. Less than monthly, but not never
☐ Abroad	____	5. Never
☐ Don't know		6. Don't know

12. How many people do you usually have contact with every day? (have a face-to-face conversation)

☐ < 5 ☐ 6-10 ☐ 11-20 ☐ 21-40 ☐ >40 ☐ Don't know

PART 2: SOCIAL CONTACT DIARY

(parental-proxy questionnaires for 0 to 10 year olds, self-reported or under supervision of parental-proxy for 11 and above**)**

INSTRUCTIONS

1) A contact is defined as:
   - EITHER **skin-to-skin contact** (physical contact, such as a handshake, hug, kiss)
   - OR **face-to-face conversation** with three or more words exchanged (non-physical contact). Ignore conversations made over phones or on computers.
2) Record in the contact diary **every person** you have contact with on your assigned day, regardless of whether the contact was long or short, and whether you know the person or not.
3) If you contact the same person several times during the assigned day, only record him/her once, and record the total time you spent with that person over the entire day. If you have both physical and non-physical contact with the same person, just record physical contact. So each person you meet during the day and have contact with should have one line in the diary.

The actual date you fill in the questionnaire:

_____ (DD) / _____ (MM)

13. Do you have a chance (e.g. students, waiters, doctors, or participating in an event with many contacts with people) through which you have a large number of contacts (more than 20)? Called "group contacts".

☐ Yes ☐ No (if no, please skip to Q16)

14. If yes, please make an estimate of the average number of "group contacts" on your assigned day?
_______ Persons.

Please note that you need not to record every "group contact" in the Q4, but only record the general (non-group) contacts, such as your family or friends.

15. Please tick in which of these age categories these group contacts mostly occur (multiple options possible):

☐ 0-5 y ☐ 6-11 y ☐ 12-18 y ☐ 19-60 y ☐ Over 60 y ☐ Don't know

16. Provide contact details by filling in the table (up to 40 contacts).
Please fill in time order.

#	When	Contact demographics	Contact details	How often do you have contact with this person?
1	☐ 5:00-8:00<br>☐ 8:00-12:00<br>☐ 12:00-18:00<br>☐ 18:00-21:00<br>☐ 21:00-5:00	**Age:** ____ or _____ – ____ years<br>**Sex:** ☐ Male ☐ Female<br>**Member of :**<br>☐ Home ☐ Student hall<br>☐ Friends ☐ Shared apartment<br>☐ Work ☐ Others	**Contact type:** ☐ Skin-to-skin ☐ Non-physical<br>**Total time:**<br>☐ <5 mins<br>☐ 5-14 mins<br>☐ 15-59 mins<br>☐ 1-4 hrs<br>☐ > 4 hrs<br>**Where:**<br>☐ Home<br>☐ Work<br>☐ School<br>☐ Transport<br>☐ Other public place<br>☐ Others	☐ Daily or almost daily<br>☐ Once or twice a week<br>☐ Once or twice a month<br>☐ Less than once a month<br>☐ Never met before

Before submitting the contact diary, please make sure you have not left out any contacts.

17. Have you included all contacts?

☐ Yes
☐ No, approximately how many people you have left out: ____________________________________

18. How well do you recall your contacts?

☐ Very well ☐ Well ☐ Moderate well ☐ Not well ☐ Poorly

PART 3: HUMAN-ANIMAL CONTACT DIARY

INSTRUCTIONS

1) Record in the contact diary **every animal** you have touched on your assigned day, regardless of whether the contact is long or short, and whether the animal belongs to you or not.
2) **Animal ownership** is defined as having at least one live animal in the household in which you are spending the majority of his/her time (i.e. living together in house).
3) **Animal touching** is defined as physical touching at least one living animal on the assigned day, irrespective of whether that animal belongs to the household.

19. Do you own some animals?

☐ Yes ☐ No (if no, please skip to Q9)

20. Please provide the details on the animals you own.

Animal:	How many:
☐ Cat	____________
☐ Rat	____________
☐ Dog	____________
☐ Fish	____________
☐ Rabbit	____________
☐ Bird	____________
☐ Pigeon	____________

- [ ] Chicken ____________
- [ ] Duck ____________
- [ ] Goose ____________
- [ ] Pig ____________
- [ ] Cow ____________
- [ ] Sheep ____________
- [ ] Horse ____________
- [ ] Monkey ____________
- [ ] Others:___ ____________

21. How often do you usually touch these live animals below (if all of them is "never" please skip this question)?

Animal: **How often (fill in the No. on the right):**

- [ ] Cat ____________
- [ ] Rat ____________
- [ ] Dog ____________
- [ ] Fish ____________
- [ ] Rabbit ____________
- [ ] Bird ____________
- [ ] Pigeon ____________
- [ ] Chicken ____________
- [ ] Duck ____________
- [ ] Goose ____________
- [ ] Pig ____________
- [ ] Cow ____________
- [ ] Sheep ____________
- [ ] Horse ____________
- [ ] Monkey ____________
- [ ] Others:___ ____________

1. Daily or almost daily
2. Once or twice a week
3. Once or twice a month
4. Less than once a month

22. Have you touched any live animal on your assigned day?

☐ Yes ☐ No (If no, please skip Q13)

23. Please provide animal touching details by filling in the table (up to 10 animal species):

#	Category of animal	Contact details	How often do you have contact with this animal?
1	☐Cat ☐Pigeon ☐Cow<br>☐Rat ☐Chicken ☐Sheep<br>☐Dog ☐Duck ☐Horse<br>☐Fish ☐Goose ☐Monkey<br>☐Rabbit ☐Pig ☐Others<br>☐Bird	**How many:** ______<br>**Total time:**<br>☐ <5 mins<br>☐ 5-14 mins ☐ 1-4 hrs<br>☐ 15-59 mins ☐ > 4 hrs<br>**Where:**<br>☐ Home<br>☐ Work ☐ Transport<br>☐ Park ☐ Others	☐Daily or almost daily<br>☐Once or twice a week<br>☐Once or twice a month<br>☐Less than once a month<br>☐Never contacted before

24. How well do you recall your animal touching?

☐ Very well ☐ Well ☐ Moderate well ☐ Not well ☐ Poorly

25. How would you describe your health today?

☹Bad 0—1—2—3—4—5😐—6—7—8—9—10 Good☺

Bad reasons: ____

26. Today's weather:

☐ Sunny day ☐ Cloudy day ☐ Rainy day ☐ Snowy day ☐ Don't know

27. Is today a regular day (have a common life pattern at least four days of a week) or irregular for you?

☐ Regular day ☐ Irregular day

Do you have any problem or suggestion regarding the questionnaire?

____

____

---END---

Good job and thank you for your cooperation again. ☺☺☺☺☺
